# Effects of facial expression and gaze interaction on brain dynamics during a working memory task in preschool children

**DOI:** 10.1371/journal.pone.0266713

**Published:** 2022-04-28

**Authors:** Koji Kashihara, Yoshitaka Matsuda

**Affiliations:** 1 Graduate School of Technology, Industrial, and Social Sciences, Tokushima University, Tokushima, Japan; 2 College of Information Science and Engineering, Ritsumeikan University, Kusatsu, Shiga, Japan; 3 Center for Baby Science, Doshisha University, Kizugawa, Kyoto, Japan; Daegu University, REPUBLIC OF KOREA

## Abstract

Executive functioning in preschool children is important for building social relationships during the early stages of development. We investigated the brain dynamics of preschool children during an attention-shifting task involving congruent and incongruent gaze directions in emotional facial expressions (neutral, angry, and happy faces). Ignoring distracting stimuli (gaze direction and expression), participants (17 preschool children and 17 young adults) were required to detect and memorize the location (left or right) of a target symbol as a simple working memory task (i.e., no general priming paradigm in which a target appears after a cue stimulus). For the preschool children, the frontal late positive response and the central and parietal P3 responses increased for angry faces. In addition, a parietal midline *α* (Pm*α*) power to change attention levels decreased mainly during the encoding of a target for angry faces, possibly causing an association of no congruency effect on reaction times (i.e., no faster response in the congruent than incongruent gaze condition). For the adults, parietal P3 response and frontal midline *θ* (Fm*θ*) power increased mainly during the encoding period for incongruent gaze shifts in happy faces. The Pm*α* power for happy faces decreased for incongruent gaze during the encoding period and increased for congruent gaze during the first retention period. These results suggest that adults can quickly shift attention to a target in happy faces, sufficiently allocating attentional resources to ignore incongruent gazes and detect a target, which can attenuate a congruency effect on reaction times. By contrast, possibly because of underdeveloped brain activity, preschool children did not show the happy face superiority effect and they may be more responsive to angry faces. These observations imply a crucial key point to build better relationships between developing preschoolers and their parents and educators, incorporating nonverbal communication into social and emotional learning.

## 1. Introduction

In preschool children, executive functioning during the early stages of development is crucial for building social relationships and predicting future academic success [1–3]. In particular, executive functions related to working memory abilities, such as paying attention to a task or stimuli and inhibiting attention to irrelevant activities, may play a key role in developing academic skills and fostering educational achievement throughout early childhood and adolescence [4,5]. Working memory is associated with an advanced brain system that involves temporary memory retention, mental manipulation of information, and various cognitive functions necessary for daily living (e.g., attention, inhibitory control, planning, and reasoning) [6]. These working memory abilities change with age, from early childhood to young adulthood [7,8]. For example, human prefrontal working memory systems develop at around 4 years of age and considerably improve from 5 to 7 years of age [9]. Brain structures such as gray and white matter volumes greatly change in preschool ages [10], and the prefrontal brain system (e.g., pruning of synaptic connections) rapidly develops [11]. It is therefore important to assess the development of executive functions in preschool children and focus on the neural dynamics of cognitive functions such as attention and working memory.

One crucial executive function that children must develop is the emotional control of social recognition, which involves sympathy, empathy, and nonverbal communication (such as recognizing facial expressions and gaze). In particular, facial expressions are critical nonverbal communication tools that allow people understand others’ feelings. In children, emotional control and dysregulation can be measured by examining attention performance in tasks with emotional distracters. For example, children aged 3 to 9 years can resist temptation and shift attention away from an attractive but prohibited item [12], and negative emotions can be controlled during adolescence [13]. In contrast, increasing attention toward distracting negative emotional events may exhaust the resources for voluntarily controlling emotion [14,15]. The maturation of attention systems interconnected with the limbic and frontal motivational systems supports self-regulation and emotional control [16,17], and increased attention and neural activity can affect the allocation of cognitive resources to control emotion [18,19]. Understanding the neural correlates of emotional control is a crucial component of assessing healthy development in childhood [20], yet the neural dynamics of facial expression and gaze interaction remain debatable in preschoolers.

In behavioral studies, congruency effects observed in priming tasks can be defined as shorter reaction times in congruent tasks or longer reaction times in incongruent tasks [21]. This effect can be found by calculating the invalid reaction time minus the valid reaction time, and it can be tested even in emotional regulation tasks. The threat hypothesis states that the human cognitive system prioritizes processing angry faces to detect threats quickly [22]. In the negativity hypothesis, other people’s distressing emotional experiences attract attention regardless of whether danger exists, and the emotionality hypothesis proposes that positive facial expressions can capture attention as effective as negative ones. This effect, known as the happy face superiority effect, occurs in paradigms examining choice-reaction times and visual search for faces [23]. Adolphs [24] suggested that this advantage occurs because happy expressions have few overlapping features with other expressions.

The effects of facial expression and gaze interaction as social emotion activities have been investigated in adults, and these studies have reported mixed results. Investigators have found that emotional faces have significant effects on facilitating or decelerating gaze direction cues [25,26], and other research has shown that emotional faces affect only gaze congruency effects [27]. Specially, in a study on adults, angry expressions held attention for a long time, causing delayed saccade latencies [25]. In a gaze cueing paradigm with facial expressions (i.e., anger, fear, joy, neutral, and surprise), gaze orienting (congruency) effects decreased linearly during the developmental stage between the ages of 7 and 13 years [28]. Moreover, in children aged 6 to 7 years, angry faces can lead them to pay attention to inverted gaze direction to prevent an emotionally negative place [29], which may be similar to the inhibition of return phenomenon observed in oculomotor responses to negative stimuli [30,31]. However, the effects of facial expression and gaze interaction are ambiguous, especially during early childhood, and it is necessary to consider which task types and target ages bring about different unstable responses. In addition, stimulus onset asynchrony (SOA), which is the time between the onset of the cue stimulus and the onset of the reaction signal, in gaze cueing tasks can affect behavioral responses [25,27]. In particular, many cognitive tasks designed to study social situations must be completed while targets and other information are presented simultaneously (i.e., SOA = 0 ms), such comparing a priming paradigm with a task that involves ignoring irrelevant obstacles or facilitating an activity based on attracting attention or generating emotions.

Kahneman [32] claimed that attention level is influenced by the total amount of information-processing resources in the human brain. For instance, the capacity for allocating attentional resources was distributed during a dual task, causing insufficient attention [33]. Event-related potentials (ERPs) on an electroencephalogram (EEG) can estimate several stages of human brain information processing. In general, ERPs reflect brain activation in early perceptual stages (N1 and P2 components) as well as higher cognitive stages (N2 and P3 components). For adults, the frontal and parietal N1 responses are sensitive to allocated attention [34], and the P2 component in the parieto-occipital regions corresponds to working memory encoding [35]. The N1 and P2 components appear not to be adequately assessed as the executive functions of visuo-spatial working memory tasks in early childhood compared to auditory ERPs (e.g., [36]). However, some reports have described the features of early ERPs in preschool children. For example, increased N1 and decreased P2 amplitudes in the frontal area were observed in a conflict-related task (i.e., a directional Stroop task) for preschoolers aged 4 years [37], suggesting that typical neural processing is linked with early attention to the task. On the other hand, the effect of flankers on the frontal N1 component was not significantly observed in this age group [38]. In addition, the P2 amplitude decreases with age (from 6‒8 to 13‒16 years of age), meaning that fewer attentional resources are allocated to successful performance in working memory tasks [39]. Given these findings, it appears that task- and age-related changes in early ERPs could occur between preschool and primary school ages [40].

The frontal N2 component is closely involved with attention focusing, inhibitory control, and conflict detection [41]. The P3 amplitude, originating from the temporal-parietal junction and lateral prefrontal cortex [42], increases during sustained attention and inhibitory control, although it attenuates with increasing task demands [43,44]. In general, P3a is activated by novel stimuli [45], and P3b reflects the attentional resource allocation toward the updating of working memory contents [46]. During early childhood, the N2 and P3 components seem to become the primary indices of ERPs during working memory tasks for attention and inhibitory control. Fronto-parietal NoGo-N2 effects were detected in children aged from 5 to 7 years [47]. A greater N2 amplitude at the central site was observed for incongruent versus congruent flankers, but only in children older than 6 years of age [48], which suggests the boundary age to acquire such an ability. The parietal P3 component, as well as the frontal and central N2 components, changed during a Stroop task in preschoolers aged 4 years [37], indicating later ERP components associated with conflict especially during incongruent trials. These results also reflect collaboration among the central, parietal, and prefrontal (specifically the anterior cingulate cortex [ACC], mainly in the N2 component [48]) cortices. Therefore, the N2 and P3 effects may be useful as simple biomarkers for prefrontal cortex development [48] and academic achievement [49], even in early childhood. However, the neural dynamics remain controversial for early and late ERPs during working memory tasks (visual attention focusing) and inducing social emotions (facial expression and gaze interaction), especially in preschoolers.

Some investigators have tested ERPs for facial expressions in preschoolers. For example, Batty and Taylor [50] investigated ERPs in children aged 4 to 15 years during an implicit processing task with emotional faces. They found occipital P1 changes in the younger children, although this effect disappeared with age, and early emotional processing differed between the young children and adolescents. For neutral and happy faces, a later frontal slow wave was more positive across age groups, suggesting that the neural processing related to perceiving emotional faces develops unstably throughout childhood, compared to the stable adult pattern. Todd et al. [51] assessed ERPs to facial expressions and personal familiarity (i.e., mothers’ and strangers’ happy and angry faces) during a Go-NoGo task for children aged 4 to 6 years and found that early perceptual components were larger in strangers’ faces. Angry mothers’ faces triggered a large mid-latency frontocentral negativity, implying that an angry parent can facilitate attentional monitoring and recognition memory. In addition, angry faces activated a right-lateralized late positive component, suggesting extended processing of negative social stimuli. The right-lateralized mid-latency negativity was greater in the NoGo trials than in the Go trials and was greater for angry faces than for happy faces, implying the presence of increased effortful attention control. These results indicate that children aged 4 to 6 years can develop overlapped or differentiated neuronal networks for speedy and detailed socio-emotional information processing. Furthermore, Dennis et al. [20] evaluated ERPs associated with emotional control in children aged 5‒9 years. In this attention task, they found greater P1 at occipital leads and Nc amplitudes at central leads in response to fearful and sad faces, reflecting more effective emotional control. Although some studies have increased our knowledge of emotional neural processing and we know that young children can perceive emotions in others and process facial expressions according to internal states, the brain neural dynamics of working memory and the neurodevelopment of expression recognition [20,37] still remain unknown, especially for developing preschool children.

It is important for developing children to acquire nonverbal communication skills so that they can shift attention correctly according to gaze direction and recognize emotional facial expressions [28,29]. Preschool children have the ability to recognize emotional expressions and pay attention to gaze [50], although this brain activity continues to develop with age. Regarding behavioral studies on the effects of facial expression and gaze interaction in healthy adults, the results seem to be roughly divided into two interpretations. A clear special cueing effect has been shown for gaze direction but not for facial expressions [27], suggesting that gaze direction processing for attention shifts is independent of facial expressions (i.e., no interaction). Conversely, stronger attention-orienting of gaze direction has been shown in response to fearful or angry faces compared with happy or neutral faces, depending on the participants’ anxiety levels [52]. Some studies have evaluated the functional effects of social communication on ERPs in early childhood, and one found that cognitive emotions in children aged 4 years changed the N2 response [51,53]. Klucharev and Sams [54] reported that the interactive ERP processing between gaze and facial expressions for young adults could be independent during the early stages and integrate later (> 300 ms) around the fusiform gyrus or the superior temporal gyrus. The combination of a working memory load induced by an N-back task and emotional pictures to induce affective valence changed the frontal *θ* and parietal *α* powers in adults [55]. It is, however, still unclear how the brain dynamics of preschoolers are modulated during visuo-spatial working memory tasks with emotional stimuli. Further studies are needed to solve the controversial problem about the effects of ‘facial expression and gaze interaction’ on brain neural activities during working memory tasks and facial emotion perception [20,50,51].

Although ERP data provide knowledge about neural dynamics, they might not sufficiently characterize signals from a time-course analysis because of overlapping waves consisting of a combination of positive and negative waves [56,57]. Although a frequency analysis is efficient for elucidating the neural mechanisms under time-invariant conditions, the momentary information on ERPs may disappear in the frequency analysis. By contrast, a time-frequency analysis can characterize the specific EEG dynamics. The frontal midline theta (Fm*θ*) power [58] after a wavelet-based analysis can particularly predict the specific brain activity associated with attention/concentration, and it can be observed as the oscillation signal between 4 and 8 Hz at the frontal midline area on the scalp [59,60]. The induced responses after a wavelet transform can identify the temporal features of attention levels during working memory tasks, and the Fm*θ* power generally increases with the amount of working memory load during the retention periods of targets [61].

Working memory tasks modulate parietal midline *α* (Pm*α*) power as well as the Fm*θ* power [55], and Pm*α* power changes depend on task quality and conditions (e.g., encoding, operation, retention, retrieval, etc.). The *θ* power in EEG is well correlated with *α* power during cognitive tasks [62]. The extent of the increase in the frontal *θ* power depends on the task difficulty, and in return, the parietal *α* power decreases [63]. By contrast, the frontal and parietal *α* powers increase during the retention period of simple working memory tasks [64]. The ACC and dorsolateral prefrontal cortex (DLPFC) activities during a working memory task could improve throughout the developmental stages of childhood [65] and may generate such brain rhythms. However, less is known about the *θ* and *α* rhythms in preschool children [66]. Furthermore, the relationships between aging and each frequency based on the induced EEG responses (i.e., Fm*θ* and Pm*α*) remain unknown in the cognitive processes of preschoolers.

This study was aimed at clarifying the brain dynamics of normally developing preschoolers during a visuo-spatial working memory task related to nonverbal communication skills (i.e., facial expression and gaze interaction). For the interference (or facilitatory) stimuli in the working memory task, we used emotional faces (neutral, angry, and happy) with congruent and incongruent gaze directions toward a target to change the extent of attentional resource allocation. In addition to typical ERPs, we quantitatively measured the induced Fm*θ* and Pm*α* powers to visualize the attention/concentration level during the encoding and temporal memory of a target place. To estimate the response time and accuracy, we performed an EEG study followed by a behavioral study. We hypothesized that we would find positive effects of facial expression and gaze interaction on neural dynamics during an attention task, even in preschoolers. We expected that developing children would show different responses, compared to the results of adults showing increased Fm*θ* power and decreased Pm*α* power (e.g., [55,61]), as well as distributed attention and modulated ERPs associated with working memory and emotional regulation (e.g., [20,50,51]). We also predicted that emotional faces and gaze would be associated with the congruency effects in preschoolers as well as adults. However, the responses to incongruent stimuli would be complicated especially during the developmental stage of brain activity (e.g., emotion and working memory processes including those interactions) in preschoolers [20,37,50]. Moreover, if a target with facial expressions and gaze simultaneously appeared in an easy task (i.e., no usual priming paradigm), irregular responses would be observed in brain and behavioral activities in preschoolers (e.g., angry face disadvantage, happy face advantage, the effects of perceptual load, and the ability to ignore obstacles) compared to young adults, who are in a mature stage of development.

## 2. Materials and methods

To clarify brain activity during a working memory task requiring visuospatial attention-shifting, we performed an EEG study followed by a behavioral study. The time spent recognizing a target during the EEG study could be predicted from the reaction time in the additional behavioral study (i.e., classifying the encoding period from the retention period). Because pressing an answer button (i.e., electromyography) can affect an EEG recording, the behavioral study was performed separately to estimate the reaction time and accuracy for each task. To roughly estimate the necessary sample size, prior to the experiments, we assumed an effect size of 0.8 (i.e., large effect) and *p* < .05 in a paired *t*-test with a power (1 - *β*) of 0.8, where *β* is the chance of making a type II error (i.e., false negative rate). Given the limited sample size, our motivation for this study was to abstract the experimental conditions that clearly showed a large effect size rather than define statistical difference based on a large sample size. Although a small effect size indicates limited practical applications, a large effect size reveals that a research finding has practical significance [67]. Therefore, to detect experimental conditions with large effect sizes, as shown in previous reports on children [68,69], we devised the experimental plan and set Cohen’s *d* as 0.8. By using the G*Power program [70], this power analysis produced a result of around 15 as the total sample size, which became a rough indication of the number of experimental participants.

### 2.1 EEG study

#### 2.1.1 Participants

The participants of the EEG study were 17 preschool children [12 females and 5 males including two left-handed children; mean age (± SD) of 66.7 ± 5.0 months] and 17 right-handed young adults of Doshisha University [12 females and 5 males; mean age (± SD) of 20.8 ± 1.0 years]. All participants had normal or corrected-to-normal vision and no history of serious medical problems. The child participants attended kindergarten or nursery school and lived close to the Center for Baby Science in Kizugawadai, Kizugawa, Kyoto, Japan. This study was approved by the research ethics committee of Doshisha University (identification number: 17018–1). Written informed consent was obtained from the parents (i.e., legal guardians) of the children and the young adult participants after they were provided with a sufficient description of the experiment.

#### 2.1.2 Visual stimuli

**Fig 1:**
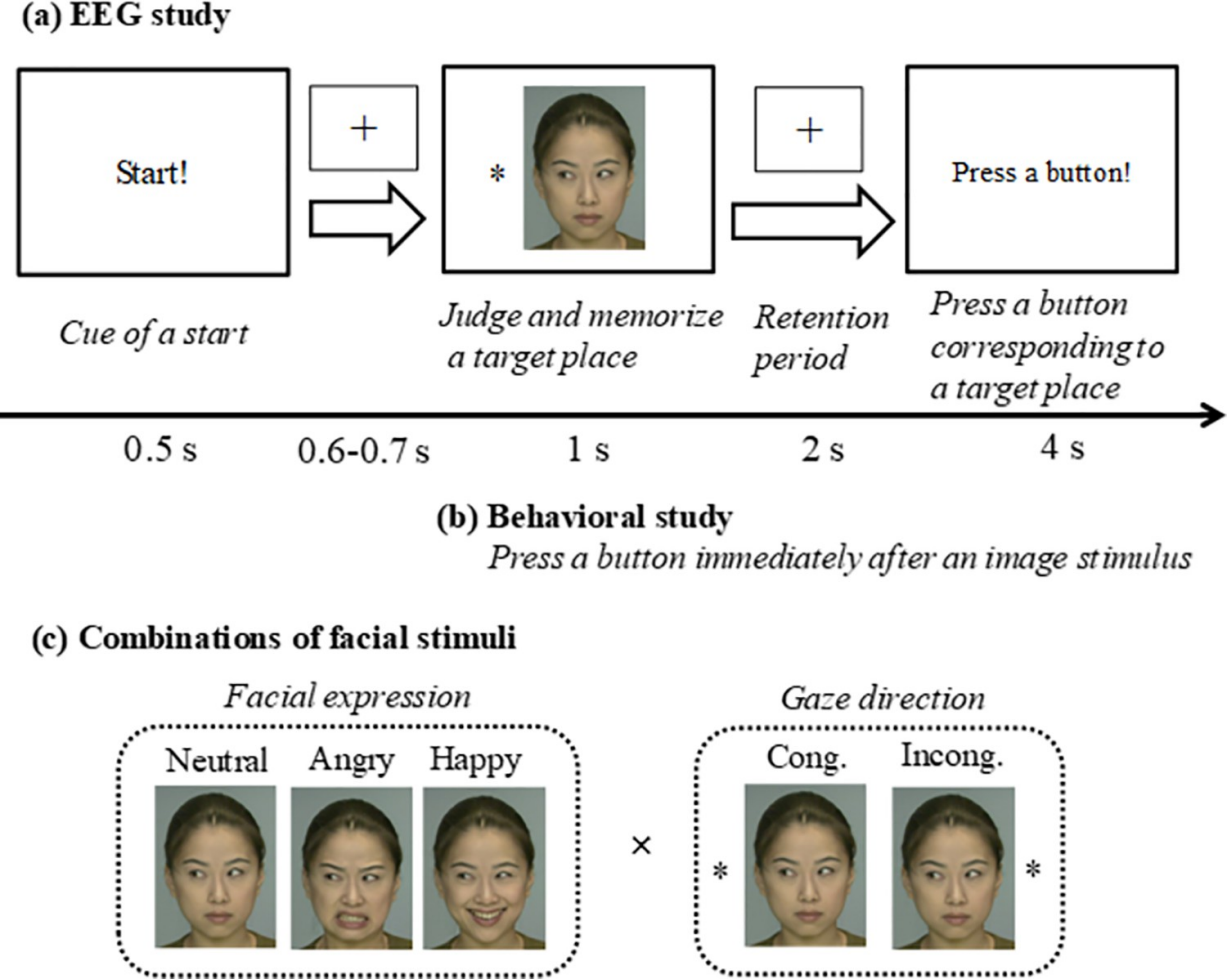
Experimental procedures for (a) EEG and (b) behavioral studies. One of the (c) multiple facial images was presented at each trial: a neutral, angry, and happy face with either a congruent (50%) or incongruent (50%) gaze direction toward a target (left or right). Reprinted from the ATR Facial Expression Image Database DB99 under a CC BY license, with permission from ATR-Promotions Inc., original copyright (2006).

We selected the images of emotional faces (i.e., neutral, angry, and happy) with a lateral gaze direction (i.e., left and right) featuring two females from the ATR Facial Expression Image Database (DB99) (ATR-Promotions, Kyoto, Japan). The target stimulus was the symbol of an asterisk (*), which appeared on the left or right side of the presented face (see Fig 1). A sound-paired, animated attention getter (Tobii Technology) was displayed before each trial, to prompt the participants to look at the center of the screen. All the images were converted into color bitmap images (640 × 640 pixels, a resolution of 1024 × 768 pixels, and constant luminance). The visual stimuli were presented on a 19-inch monitor positioned at the same height as the participants’ eyes. The distance from the visual stimuli was set at 120 cm (i.e., a visual angle less than 5°).

#### 2.1.3 EEG recording

EEG signals were recorded at a sampling rate of 500 Hz, using biological amplifiers with an analog band-pass filter between 1 and 35 Hz (BIOPAC MP150 with EEG100C). EEG electrodes were placed at the Fz, Cz, and Pz sites in accordance with the international 10–20 system in a sealed room, and electrode impedances were kept below 20 kΩ. The EEG waveforms in the midline frontal, central, and parietal lobes were of particular interest because they strongly reflect neuronal activity for working memory and attention (e.g., frontal *θ* and parietal *α* frequencies [46,61,64] as well as ERPs [48]). Electrooculography was recorded from the right eye to discard eye movement and blink artifacts with an independent component analysis. The earth electrode was placed on the left earlobe, and the right earlobe was the reference point for the EEG recording. To ensure safety and reduce anxiety in child participants, a parent was present in the sealed room but did not get in the way of the experiment.

#### 2.1.4 Experimental procedures

The sensors for EEG recording were attached to the participants. The participants were asked to practice the experiment (at least 10 trials for children and 5 trials for adults) during an acclimation period. We then performed the EEG experiment to evaluate attention-shifting abilities during the working memory task. Fig 1 shows the experimental procedure for a single trial of EEG recording. A ‘Start!’ stimulus of 500 ms was followed by a jittering period (the presentation of ‘+’ as a fixation cross) between 600 and 700 ms. A face [i.e., neutral, angry, or happy face with a left or right gaze congruent (50%) or incongruent (50%) with the target direction] was randomly presented onscreen for 1 s, followed by a memory retention period of 2 s (‘+’ as a fixation cross). The participants were then asked to press a left or right button using the thumbs of both hands, corresponding to the laterally presented target (‘*’) direction. The time limit to answer was set to 4 s. An attention getter was used to inform the participant to start the next trial (a resting interval of 4 s). One block consisted of 24 trials (4 trials under each stimulus condition). The next block was started after the participants finished a rest period of a few minutes. In total, each participant performed five blocks. The number of trials and blocks was based on preliminary experiments and data analyses, considering the relatively slow EEG waves such as *θ* and *α* bands. At the end of the experiment, the experimenter verbally asked the children to judge the emotional expressions of the randomly presented faces used for this experiment. The experimenter told the children whether the face in the picture was smiling, angry, or neither (i.e., neutral). The children answered ‘yes’ or ‘no’, and the experimenter manually recorded their responses.

#### 2.1.5 The children’s behavior questionnaire for attention

For the child participants, the parents completed the attention-focusing and attention-shifting subscales of the Children’s Behavior Questionnaire (CBQ) [71]. The attention-focusing subscale measures the ability to concentrate on a given task and consists of 9 items (item numbers: 16, 38R, 47R, 125, 144, 160, 171R, 186, and 195R, with ‘R’ indicating reversed scoring). The attention-shifting subscale assesses attention-shifting ability and consists of 5 items (6R, 29, 95R, 180, and 184R). The parents rated how true each item was for their child by using a 7-point scale from 1 (never) to 7 (always). For the CBQ ratings, we calculated the grand average (± SD) across all items and participants (*n* = 17, 4.5 ± 0.5). The results show typical development in the preschoolers regarding attention level [72,73]. Table 1 summarizes the average values of the attention-focusing and attention-shifting subscale items (S1 File). We used these data to confirm and specify the children’s attention levels from the viewpoint of their mothers. Their attention levels were average and the values did not reveal abnormal attention levels compared with other reports [72,73].

**Table 1:** The CBQ rating items and scores. Seven scales (S1 Fig).

Category	Item No.	Contents of Questions (My child:)	Average ± S.D.
The attention-focusing subscale	16	When picking up toys or other jobs, usually keeps at the task until it’s done.	4.9 ± 1.5
	38R	When practicing an activity, has a hard time keeping her/his mind on it.	4.8 ± 1.4
	47R	Will move from one task to another without completing any of them.	4.6 ± 1.2
	125	When drawing or coloring in a book, shows strong concentration.	4.8 ± 1.5
	144	When building or putting something together, becomes very involved in what s/he is doing, and works for long periods.	3.9 ± 2.0
	160	Has difficulty leaving a project s/he has begun.	3.9 ± 1.2
	171R	Is easily distracted when listening to a story	6.1 ± 0.8
	186	Sometimes becomes absorbed in a picture book and looks at it for a long time.	5.2 ± 0.8
	195R	Has a hard time concentrating on an activity when there are distracting noises.	4.1 ± 1.4
The attention-shifting subscale	6R	Is hard to get her/his attention when s/he is concentrating on something.	4.1 ± 1.3
	29	Can easily shift from one activity to another.	4.0 ± 1.5
	95R	Has a lot of trouble stopping an activity when called to do something else.	3.2 ± 1.5
	180	Has an easy time leaving play to come to dinner.	4.9 ± 1.5
	184R	Sometimes doesn’t seem to hear me when I talk to her/him.	4.2 ± 1.1
Total items for attention	All	‒	4.5 ± 0.5

‘R’ of the item numbers: a reversed score.

### 2.2 Behavioral study

This part of the study included 11 children (7 females, 4 males) aged 68.5 ± 4.5 months (mean ± SD) and 13 young adults (10 females, 3 males) aged 20.7 ± 1.0 years. The adults performed this behavioral study after the EEG study. The children performed it on another day, considering their fatigue.

The reaction time for selecting a correct target direction was assessed by changing the facial expressions and gaze direction. The experimental procedure was the same as in the EEG study, except the participants were required to respond quickly to a target stimulus immediately after an image appeared (i.e., no memory retention period) by pressing the left or right button corresponding to the target location. After participants had enough practice, they performed the main experiment: two blocks for children and one block for adults, with 24 trials (4 trials under each condition) per block. This behavioral study included fewer trials than the EEG study, requiring sufficient noise reduction. The rest period between each block was a few minutes.

### 2.3 Data analysis and statistics

#### 2.3.1 EEG study

*Preprocessing*. The EEG signals for all the conditions were segmented into epochs ranging from 500 ms before stimulus onset to 3 s after stimulus onset (i.e., 1 s for the image presentation and 2 s for the memory retention of a target direction). Baseline correction was applied to the segmented waveform by subtracting the mean of the 500-ms pre-stimulus interval from the data after stimulus onset. Trials in which ocular activity or movement artifacts had amplitudes greater than ±80 *μ*V were excluded from later data analysis. For the ERP and wavelet analyses, the amount of valid data of all trial data were as follows: means ± SEM = 74.3 ± 1.0% in children and 98.9 ± 0.2% in adults (*p* < .001, effect size *r* = .819 according to the Mann-Whitney U test).

*Event-related potentials*. The ERPs (N1, P2, N2, and P3) at the Fz, Cz, and Pz regions were determined in accordance with the features of the averaged waveforms for this study and previous studies (e.g., [47] for children and [41] for adults). For the Fz site, we defined the minimum value between 100 and 200 ms in children (90 and 150 ms in adults) as the N1 component, the maximum value between 200 and 350 ms in children (150 and 200 ms in adults) as the P2 component, the minimum value between 300 and 500 ms in children (240 and 360 ms in adults) as the N2 component, and the mean value between 600 and 800 ms in children (500 and 600 ms in adults) as the late positive response. For the Cz and Pz sites, we focused on the significant changes in ERPs [i.e., P3, the average between 500 and 600 ms in children (500 and 600 ms at Cz; 300 and 400 ms at Pz in adults)]. After the trials were averaged for each experimental condition (six conditions: neutral, angry, and happy faces with a congruent or incongruent gaze direction toward a target), we calculated the grand average across all participants in children and adults.

*Time-frequency analysis*. A wavelet analysis can detect the appearance of Fm*θ* and Pm*α* waves from the raw EEG signals with external noise and visualize the attention level during tasks. The change in attention levels during the tasks was quantified by the time-frequency analysis of EEG signals at Fz and Pz. The analyzing period during the working memory task was divided into three parts: (i) the encoding and decision period to remove distractors (20–800 ms after the stimulus onset), (ii) the first retention period of a target (1–2 s), and (iii) the latter retention period of a target (2–3 s). For adult participants, the focused EEG activity was in the *θ* (4–8 Hz) and *α* (8–13 Hz) frequency bands. For children, the *θ* and *α* frequency bands were set at 4–6 Hz and 8–11 Hz, respectively, because of their lower frequency ranges for brain modulation compared to adults.

The signals were convoluted by a complex Morlet wavelet [60]:

$$w(t,f_{0})=\exp(\frac{-t^{2}}{2\sigma_{t}^{2}})\cdot \exp(2\pi f_{0}it)/\sqrt{\sigma_{t}\sqrt{\pi }},$$ (1)

where the SD of the time domain (*σ*_*t*_) is inversely proportional to the standard deviation of the frequency domain [*σ*_*f*_ = (2*πσ*_*t*_)^-1^]. The *f*_*0*_/*σ*_*f*_ determining the effective number of oscillation cycles comprised in the wavelet was set at 6, with *f*_*0*_ ranging from 4 to 13 Hz (i.e., *θ* and *α* bands) in increments of 0.1 Hz. After subtracting a linear trend, the continuous wavelet transform was computed as the convolution of a complex wavelet with a time series *u*(*t*):

$$\tilde{u}(t,f_{0})=w(t,f_{0})*u(t).$$ (2)

The squared norm of the wavelet transform was calculated in a frequency band at around *f*_*0*_.

Because an ‘*evoked*’ response in ERP data shows similar dynamics (i.e., latency and phase) in every trial, it can be extracted by averaging the evoked potentials. By contrast, an ‘*induced*’ response appears with a jitter in the latency among trials, and it will cancel out when averaging the evoked potentials [74]. For this study, to assess the induced EEG response, the wavelet-transform data for each trial were averaged for each task. After the baseline correction was applied to this analysis (i.e., subtraction of the prestimulus between 0 and -200 ms at every frequency), we computed the change ratio from the baseline (%). Because the analysis time windows in slower waves contained the artificial data for interpolation to complete the wavelet analysis, the data for the period from -200 to -500 ms were removed after the wavelet transform in the prestimulus data between 0 and -500 ms. It was confirmed that the baseline data reflected accurate frequency ranges by using test signals.

#### 2.3.2 Behavioral study

We evaluated the accuracies and reaction times for the working memory task for all participants. For the reaction times, outlying latencies that were greater than 200 ms in both groups, less than 3 s in children, or less than 1 s in adults were removed from later analysis. The means ± SEM in the amount of valid data for all trials were 98.5 ± 0.6% and 99.4 ± 0.5% (*p* = .055^†^ as marginally significant and *r* = .160 according to the Mann-Whitney U test) for the children and adults, respectively. The average values for the accuracies and reaction times were then computed across all participants in each group.

#### 2.3.4 Statistics

All data were expressed as mean values (± SEM), and we calculated the grand averages across all participants. A two-way analysis of variance (ANOVA) was applied to examine ERPs, wavelet-based data, and reaction times for Face (three levels: neutral, angry, and happy) × Gaze (two levels: congruent and incongruent) with a within-subject design, followed by the Holm post hoc test for multiple comparisons. For this study, the adjusted *p* values ($\hat{p}$) are shown if the levels were more than three. For the wavelet analysis, we also performed a nonparametric analysis (Steel’s test), to compare the frequency power value for each period and experimental condition to the normalized baseline value.

When comparing children with adults, we used the following non-parametric tests. The Mann-Whitney U test was used for the comparison between the two groups (children and adults). The effect size was calculated as $r=|Z/\sqrt{N}|$, where *Z* is the standardized value and *N* is the total sample number. For each evaluation index, non-parametric multiple comparisons (i.e., Steel-Dwass test) were performed between all the conditions for children and adults after confirming the significance by the Kruskal-Wallis test.

Statistical significance was mainly assigned to differences of *p* < .05. However, if an ANOVA or the Kruskal-Wallis test resulted even in a marginally significant result (*p* < .1), the post analysis was continued to confirm significant differences among experimental conditions or between groups for details: the post hoc comparisons after the simple main effects of interaction and multiple comparisons of *p* < .1. In the text, figures, and tables, we refer to cases of *p* < .05 as statistically significant and .05 ≤ *p* < .1 as marginally significant (marked as ‘^†^’). The actual *p* values for all statistical analyses are shown in the figures and tables.

## 3. Experimental results

### 3.1 EEG study

Task accuracy in the EEG study was confirmed by pressing the left or right button corresponding to the target location on the display after the memory retention period (means ± SEM of effective data numbers: 92.3 ± 1.29% for children and 99.4 ± .13% for adults, resulting in *p* < .001 and *r* = .567 according to the Mann-Whitney U test). Task accuracy was high under all conditions for both groups (96.3 ± .53% for children and 99.8 ± .10% for adults, resulting in *p* < .001 and *r* = .455 according to the Mann-Whitney U test), implying that both groups participated actively during the experiment.

#### 3.1.1 ERPs

**Fig 2:**
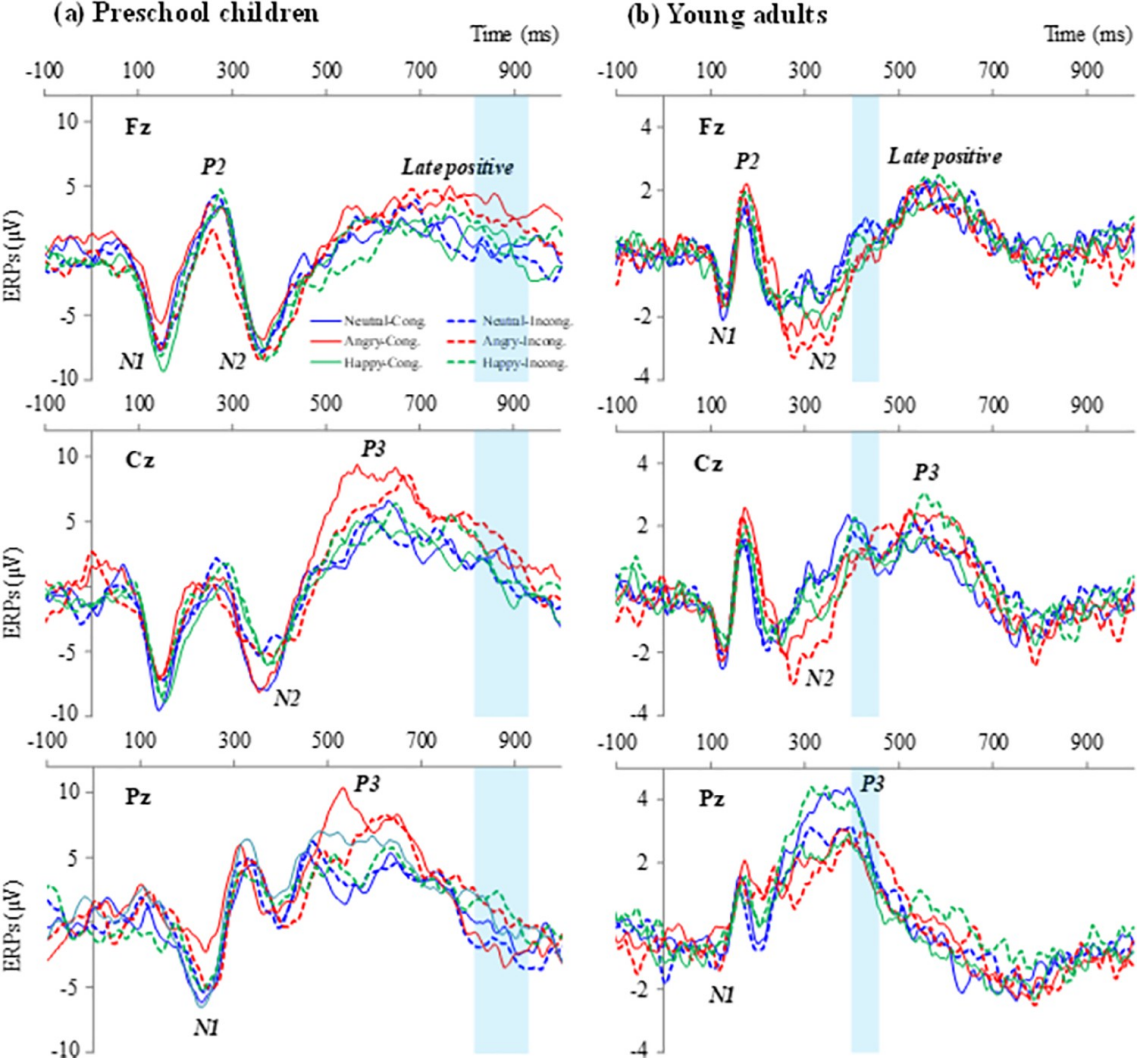
The grand averages of ERPs (N1, P2, N2, and P3) under all conditions at Fz (*upper*), Cz (*middle*), and Pz (*lower*) sites: (a) preschool children and (b) young adults. The *blue* background represents the range between the minimum and maximum values of mean reaction times for each group in the behavioral study, estimating the encoding and decision times for the EEG study.

Fig 2(A) shows the ERPs (N1, P2, N2, and P3) under all conditions at Fz, Cz, and Pz for the preschool children. Overall, the frontal N1, P2, and N2 deflections were clearly observed during the early stages of visual processing to perceive and encode a target by allocating attention resources and ignoring distractors. These deflections were followed by the late positive response as a higher cognitive process. Table 2 summarizes the statistical results for the ERPs in children (S2 File). The ANOVA showed main effects of Face (*p* = .040) for the late positive amplitude at Fz and for the P3 amplitudes at Cz (*p* = .005) and Pz (*p* = .014). The multiple comparisons showed that the P3 amplitudes were significantly greater for angry faces than for neutral ($\hat{p}$ = .010) or happy ($\hat{p}$ = .018) faces at Cz and significantly greater for angry faces than for neutral faces ($\hat{p}$ = .026) at Pz. Detailed information for the tests performed after the ANOVA is shown in Supplementary S1 Table.

**Table 2:** ERPs (*μ*V) during the working memory task for preschool children.

Items	*F* [*F*_*(2*,*32)*_ *in Face*, *F*_*(1*,*16)*_ *in Gaze*, *and F*_*(2*,*32)*_ *in Int*.]		*p*	*η*_*p*_^2^ (partial *η*^2^)	Neutral		Angry		Happy	
					Cong.	Incong.	Cong.	Incong.	Cong.	Incong.
N1 at Fz	*Face*	1.431 (.011)	.254 (.989)	.082 (< .001)	-9.0±1.5 (143.6±5.1)	-10.1±1.5 (154.7±6.3)	-7.7±1.3 (145.6±6.3)	-9.9±1.7 (151.1±6.1)	-11.3±1.9 (149.6±5.1)	-10.3±1.8 (148.0±5.3)
	*Gaze*	.340 (1.066)	.568 (.317)	.021 (.063)						
	*Int*.	.720 (.995)	.494 (.381)	.043 (.059)						
P2 at Fz	*Face*	.688 (.853)	.510 (.436)	.041 (.051)	6.5±1.2 (258.4±8.7)	7.2±0.9 (265.6±8.9)	6.6±1.5 (252.5±9.1)	4.8±1.2 (256.5±8.3)	6.3±1.3 (262.1±7.5)	7.3±1.5 (266.4±6.7)
	*Gaze*	.007 (1.483)	.936 (.241)	< .001 (.085)						
	*Int*.	.989 (.035)	.383 (.965)	.058 (.002)						
N2 at Fz	*Face*	.075 (.227)	.928 (.798)	.005 (.014)	-10.4±1.6 (396.0±12.7)	-11.0±1.5 (363.9±6.9)	-10.4±1.3 (385.5±15.0)	-11.8±1.5 (366.0±10.8)	-9.9±1.4 (367.8±7.5)	-11.4±1.3 (382.4±7.3)
	*Gaze*	1.425 (2.366)	.250 (.144)	.082 (.129)						
	*Int*.	.094 (3.273)	.911 (.051^†^)	.006 (.170)						
Late at Fz	*Face*	3.566	.040*	.182	1.8±1.1	1.9±0.8	3.7±1.3	3.8±0.9	1.4±1.1	2.0±1.3
	*Gaze*	.246	.626	.015						
	*Int*.	.053	.949	.003						
P3 at Cz	*Face*	6.271	.005**	.282	2.6±1.3	3.3±0.9	8.0±1.1	5.0±1.0	3.7±1.1	3.4±1.3
	*Gaze*	1.837	.194	.103						
	*Int*.	2.120	.137	.117						
P3 at Pz	*Face*	4.856	.014*	.233	2.3± 1.7	3.3±1.4	8.7±1.4	5.9±2.0	6.4±2.0	3.9±1.0
	*Gaze*	2.372	.143	.129						
	*Int*.	1.481	.243	.085						

**, *p* < .01

*, *p* < .05

†, *p* < .1.

(), latencies.

Fig 2(B) shows the ERPs under all conditions at Fz, Cz, and Pz in the young adults. Table 3 summarizes the statistical results of the ERPs in the young adults (S3 File). For the Fz site, the ANOVA revealed an interaction between the two factors in N1 (*p* = .098^†^) and a main effect of Face in the N2 deflection (*p* = .008). The multiple comparisons showed that the N2 amplitude for angry faces was significantly larger than that for neutral faces ($\hat{p}$ = .041). For the parietal P3 amplitude, the ANOVA indicated a main effect of Face (*p* = .040) and an interaction between the two factors (*p* = .001). There were simple main effects of Face under congruent (*p* = .027) and incongruent (*p* = .003) conditions and of Gaze for neutral (congruent > incongruent, *p* = .039) and happy (congruent < incongruent, *p* = .019) faces. The multiple comparisons clarified that the parietal P3 amplitude was smaller for happy faces than for neutral faces ($\hat{p}$ = .058^†^) under the congruent condition, and it was larger for happy faces than for angry faces ($\hat{p}$ = .007) under the incongruent condition. Supplementary S2 Table shows detailed information for the tests performed after the ANOVA.

**Table 3:** ERPs (*μ*V) during the working memory task for young adults.

Items	*F* [*F*_*(2*,*32)*_ *in Face*, *F*_*(1*,*16)*_ *in Gaze*, *and F*_*(2*,*32)*_ *in Int*.]		*p*	*η*_*p*_^2^ (partial *η*^2^)	Neutral		Angry		Happy	
					Cong.	Incong.	Cong.	Incong.	Cong.	Incong.
N1 at Fz	*Face*	.203 (.034)	.818 (.967)	.013 (.002)	-3.0±0.6 (123.4±4.2)	-2.3±0.6 (123.4±4.2)	-2.5±0.5 (127.3±3.9)	-3.0±0.5 (121.3±4.6)	-2.5±0.3 (128.0±4.2)	-2.5±0.5 (120.9±4.3)
	*Gaze*	.037 (2.219)	.851 (.156)	.002 (.122)						
	*Int*.	2.500 (.684)	.098^†^ (.512)	.135 (.041)						
P2 at Fz	*Face*	1.161 (1.304)	.326 (.286)	.068 (.075)	2.6±0.7 (172.6±3.6)	2.8±0.6 (175.9±3.6)	3.2±0.7 (178.8±3.2)	3.0±0.8 (172.2±4.1)	2.1±0.7 (179.6±4.2)	2.8±0.6 (175.6±3.3)
	*Gaze*	.892 (1.359)	.359 (.261)	.053 (.078)						
	*Int*.	.886 (2.760)	.422 (.078^†^)	.053 (.147)						
N2 at Fz	*Face*	5.588 (.790)	.008** (.463)	.259 (.047)	-3.8±0.7 (279.2±14.5)	-3.3±0.8 (280.8±10.8)	-4.3±0.8 (288.8±8.7)	-4.8±0.9 (294.8±9.8)	-3.9±0.8 (271.1±11.8)	-4.2±0.8 (280.9±13.1)
	*Gaze*	.240 (.794)	.631 (.386)	.015 (.047)						
	*Int*.	.620 (.105)	.544 (.901)	.037 (.007)						
Late at Fz	*Face*	.045	.956	.003	1.8±0.5	1.8±0.6	1.9±0.5	1.5±0.6	1.4±0.6	2.0±0.6
	*Gaze*	.029	.866	.002						
	*Int*.	1.135	.334	.066						
P3 at Cz	*Face*	1.756	.189	.099	1.3±0.5	1.8±0.6	2.3±0.5	1.8±0.5	1.4±0.6	2.4±0.6
	*Gaze*	1.249	.280	.072						
	*Int*.	2.314	.115	.126						
P3 at Pz	*Face*	3.560	.040*	.182	4.0±0.8	2.9±0.6	2.5±0.4	2.0±0.3	2.4±0.6	4.0±0.6
	*Gaze*	.006	.940	< .001						
	*Int*.	8.572	.001**	.349						

**, *p* < .01

*, *p* < .05

†, *p* < .1.

(), latencies.

#### 3.1.2 Fmθ and Pmα responses

**Fig 3:**
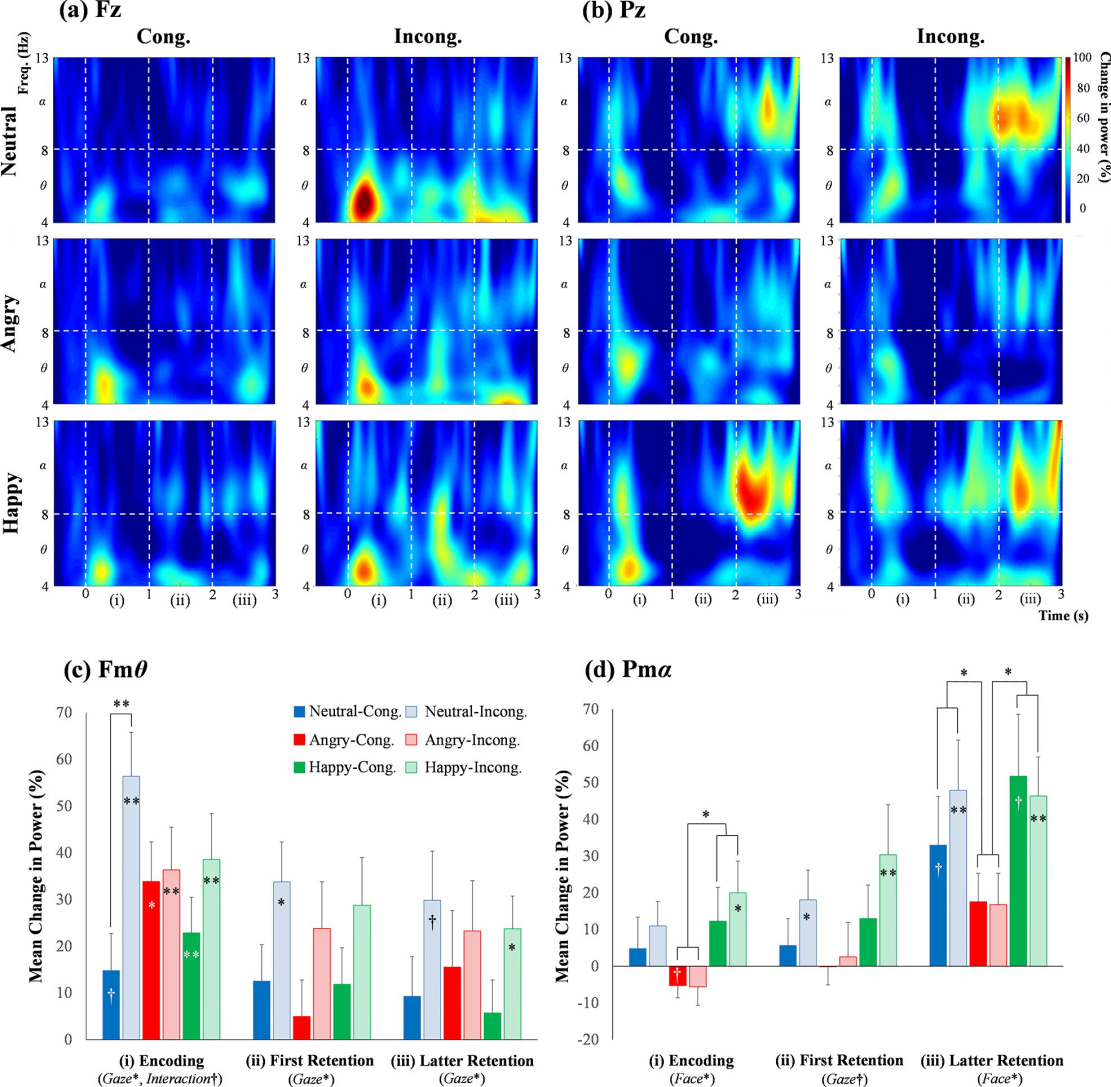
Time-course changes in the induced *θ* and *α* powers at (a) Fz and (b) Pz sites during the working memory task for preschool children: (i) encoding and decision period, (ii) first retention period, and (iii) latter retention period. The average data with statistics for the (c) Fm*θ* and (d) Pm*α* powers in each period. The symbols in squares (**, *p* < .01; *, *p* < .05; ^†^, *p* < .1), nonparametric tests versus baseline values; those in brackets, the ANOVA results.

Fig 3 (*upper section*) shows the time-course change in the induced *θ* and *α* powers at (a) Fz and (b) Pz sites during the working memory task for the children. Compared with the baseline value (Steel’s test), the Fm*θ* powers were significantly emphasized during the encoding period under the incongruent gaze conditions for all facial expressions. The Pm*α* powers under the incongruent gaze direction of happy faces were significantly increased during all periods. All *p* values from the Steel’s tests are shown in Supplementary S3 Table.

Fig 3 (*lower section*) represents the mean values (± SEM) and significance of the (c) Fm*θ* and (d) Pm*α* powers in each period for the children (S4 File). For the Fm*θ*, the ANOVA (Table 4) revealed main effects of Gaze during the encoding (*p* = .012), first retention (*p* = .013), and latter retention (*p* = .040) periods; an interaction between the two factors during the encoding period (*p* = .074^†^), followed by a simple main effect between the gaze conditions for neutral faces (*p* = .004). For the Pm*α*, the ANOVA found main effects of Face during the encoding (*p* = .046) and latter retention (*p* = .018) periods; a main effect of gaze direction during the first retention period (*p* = .072^†^). The multiple comparisons showed that Pm*α* was significantly attenuated for angry faces than for happy faces during the encoding ($\hat{p}$ = .019) and latter retention periods ($\hat{p}$ = .017). The Pm*α* was also more decreased in angry faces than in neutral faces during the latter retention period ($\hat{p}$ = .045). Supplementary S4 Table provides detailed information on the tests performed after the ANOVA.

**Table 4:** Statistics for the induced *θ* and *α* powers at Fz and Pz sites during the working memory task for preschoolers.

Items	*F* [*F*_*(2*,*32)*_ *in Face*, *F*_*(1*,*16)*_ *in Gaze*, *and F*_*(2*,*32)*_ *in Int*.]		*P*	*η*_*p*_^2^ (partial *η*^2^)	Neutral		Angry		Happy	
					Cong.	Incong.	Cong.	Incong.	Cong.	Incong.
(a) Encoding in Fm*θ*	*Face*	.291	.749	.018	14.8±7.9	56.4±9.4	33.8±8.5	36.3±9.2	22.8±7.7	38.6±9.8
	*Gaze*	8.077	.012*	.336						
	*Int*.	2.834	.074^†^	.151						
First retention in Fm*θ*	*Face*	.681	.513	.041	12.5±7.8	33.8±8.5	4.9±7.8	23.8±10.0	11.8±7.8	28.8±10.3
	*Gaze*	7.909	.013*	.331						
	*Int*.	.030	.970	.002						
Latter retention in Fm*θ*	*Face*	.236	.791	.015	9.3±8.5	29.8±10.5	15.5±12.1	23.3±10.8	5.7±7.1	23.7±7.0
	*Gaze*	5.025	.040*	.239						
	*Int*.	.193	.825	.012						
(b) Encoding in Pm*α*	*Face*	3.387	.046*	.175	4.8±8.6	11.0±6.6	-5.3±5.3	-5.6±5.9	12.2±9.2	20.0±8.6
	*Gaze*	.884	.361	.052						
	*Int*.	.248	.782	.015						
First retention in Pm*α*	*Face*	1.672	.204	.095	5.7±7.3	18.1±8.1	-0.1±5.1	2.5±10.6	13.0±9.1	30.3±13.6
	*Gaze*	3.715	.072^†^	.188						
	*Int*.	.458	.637	.028						
Latter retention in Pm*α*	*Face*	4.576	.018*	.222	33.0±13.3	47.9±13.7	17.5±8.9	16.7±11.1	51.7±16.9	46.3±10.6
	*Gaze*	.150	.704	.009						
	*Int*.	.480	.623	.029						

*, *p* < .05

†, *p* < .1.

**Fig 4:**
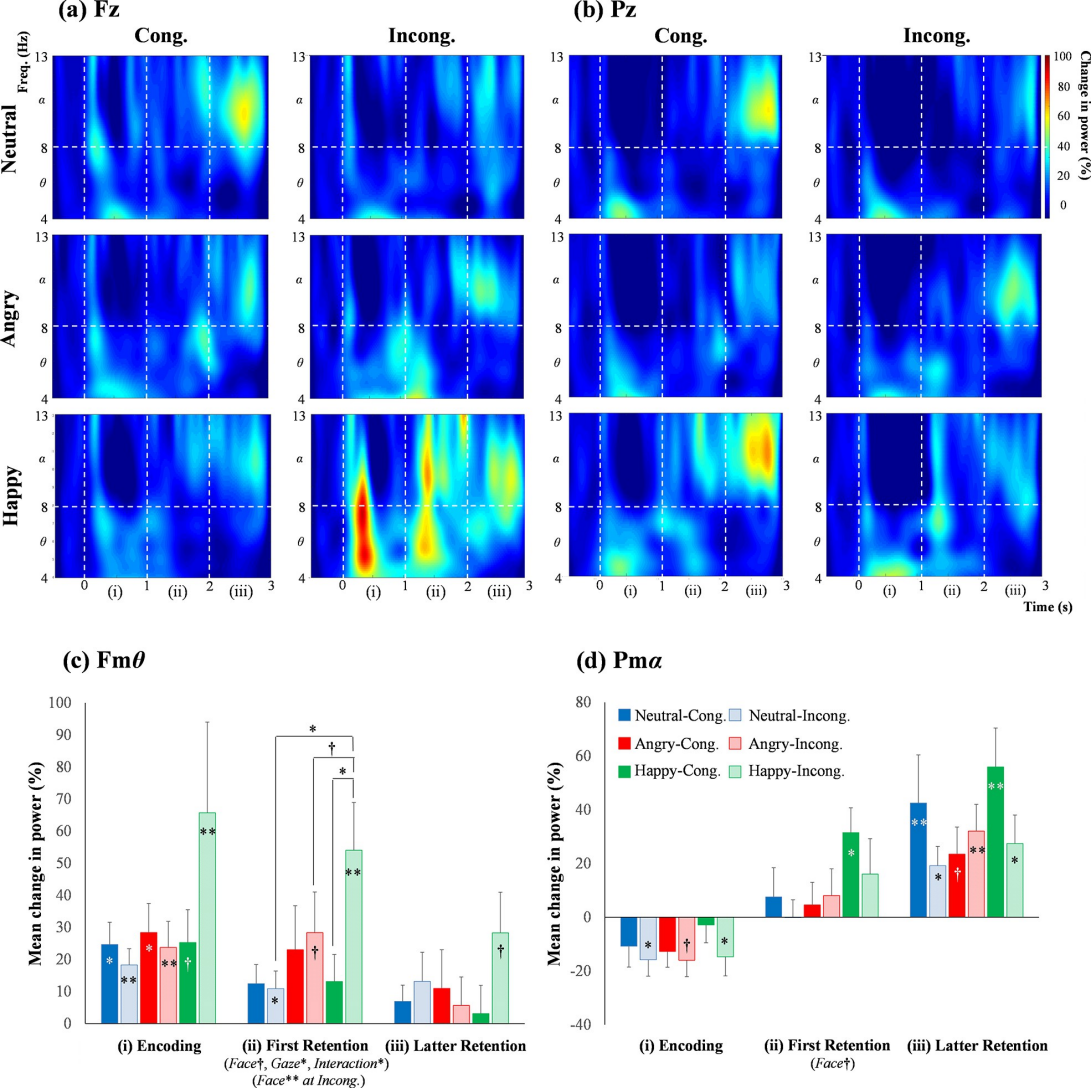
The time-course change in the induced *θ* and *α* powers at (a) Fz and (b) Pz sites during the working memory task for young adults. The average data with statistics for the (c) Fm*θ* and (d) Pm*α* powers in each period. The symbols are the same as those in Fig 3.

Fig 4 (*upper section*) shows the time-course change in the induced *θ* and *α* powers at (a) Fz and (b) Pz during the working memory task for young adults. Compared with the baseline value, the Fm*θ* powers were significantly activated during the encoding period under most conditions. Supplementary S5 Table shows all *p* values for the Steel’s tests. As a distinctive result, the Fm*θ* power increased under the incongruent gaze condition for happy faces, especially during the encoding and first retention periods. The Pm*α* powers were relatively low during the encoding period but increased linearly with the time course, resulting in the emphasized power during the latter memory retention period.

Fig 4 (*lower section*) indicates the mean values (± SEM) and significance of the (c) Fm*θ* and (d) Pm*α* powers in each period for young adults (S5 File). For the Fm*θ* during the first retention period, the ANOVA (Table 5) revealed main effects of Face (*p* = .064^†^) and Gaze (*p* = .025) and an interaction between the two factors (*p* = .026). In the multiple comparisons for Face, the Fm*θ* power was higher in happy faces than for neutral faces ($\hat{p}$ = .025). Simple main effects were found for the Gaze condition (i.e., a congruency effect) in happy faces (*p* = .015) and for Face under the incongruent gaze condition (*p* = .003). The multiple comparisons showed a greater value for happy faces than for neutral ($\hat{p}$ = .008) or angry ($\hat{p}$ = .076^†^) faces under the incongruent condition. For the Pm*α* during the first retention period, there was a tendency for a main effect of Face (*p* = .086^†^), as a result of the ANOVA. Detailed information on the tests performed after the ANOVA is indicated in Supplementary S6 Table.

**Table 5:** Statistics for the induced *θ* and *α* powers at Fz and Pz sites during the working memory task for young adults.

Items	*F* [*F*_*(2*,*32)*_ *in Face*, *F*_*(1*,*16)*_ *in Gaze*, *and F*_*(2*,*32)*_ *in Int*.]		*p*	*η*_*p*_^2^ (partial *η*^2^)	Neutral		Angry		Happy	
					Cong.	Incong.	Cong.	Incong.	Cong.	Incong.
(a) Encoding in Fm*θ*	*Face*	2.267	.120	.124	24.7±6.9	18.3±5.1	28.4±9.1	23.7±8.1	25.3±10.2	65.7±28.3
	*Gaze*	.974	.338	.057						
	*Int*.	2.047	.146	.113						
First retention in Fm*θ*	*Face*	3.001	.064^†^	.158	12.4±6.0	10.9±5.5	23.0±13.8	28.3±12.7	13.1±8.4	54.1±14.9
	*Gaze*	6.108	.025*	.276						
	*Int*.	4.098	.026*	.204						
Latter retention in Fm*θ*	*Face*	.299	.743	.018	6.9±5.1	13.1±9.1	11.0±12.1	5.7±8.8	3.1±8.9	28.3±12.7
	*Gaze*	2.240	.154	.123						
	*Int*.	1.557	.226	.089						
(b) Encoding in Pm*α*	*Face*	.593	.559	.036	-10.7±8.2	-15.8±6.8	-12.7±6.1	-16.0±5.7	-2.8±6.8	-14.7±7.5
	*Gaze*	1.462	.244	.084						
	*Int*.	.501	.611	.030						
First retention in Pm*α*	*Face*	2.646	.086^†^	.142	7.6±10.8	-0.1±6.6	4.6±8.4	8.1±10.0	31.5±9.2	16.0±13.2
	*Gaze*	.771	.393	.046						
	*Int*.	.794	.461	.047						
Latter retention in Pm*α*	*Face*	1.121	.339	.066	42.5±17.9	19.2±7.2	23.5±10.1	32.1±10.0	56.0±14.4	27.4±10.6
	*Gaze*	1.968	.180	.110						
	*Int*.	1.946	.159	.108						

*, *p* < .05

†, *p* < .1.

#### 3.1.3 Recognition of facial expressions

\At the end of the EEG experiment, all participants were asked to respond verbally to identify the emotional category (i.e., neutral, angry, and happy) of the faces presented during the experiment to confirm whether they could accurately recognize the emotions. The accuracy was almost 100%: three errors in children (two cases mistaking a neutral face for an angry face and one case mistaking a happy face for a neutral face) and three errors in adults (three cases mistaking a neutral face for an angry face).

### 3.2 Behavioral study

#### 3.2.1 Reaction times

**Fig 5:**
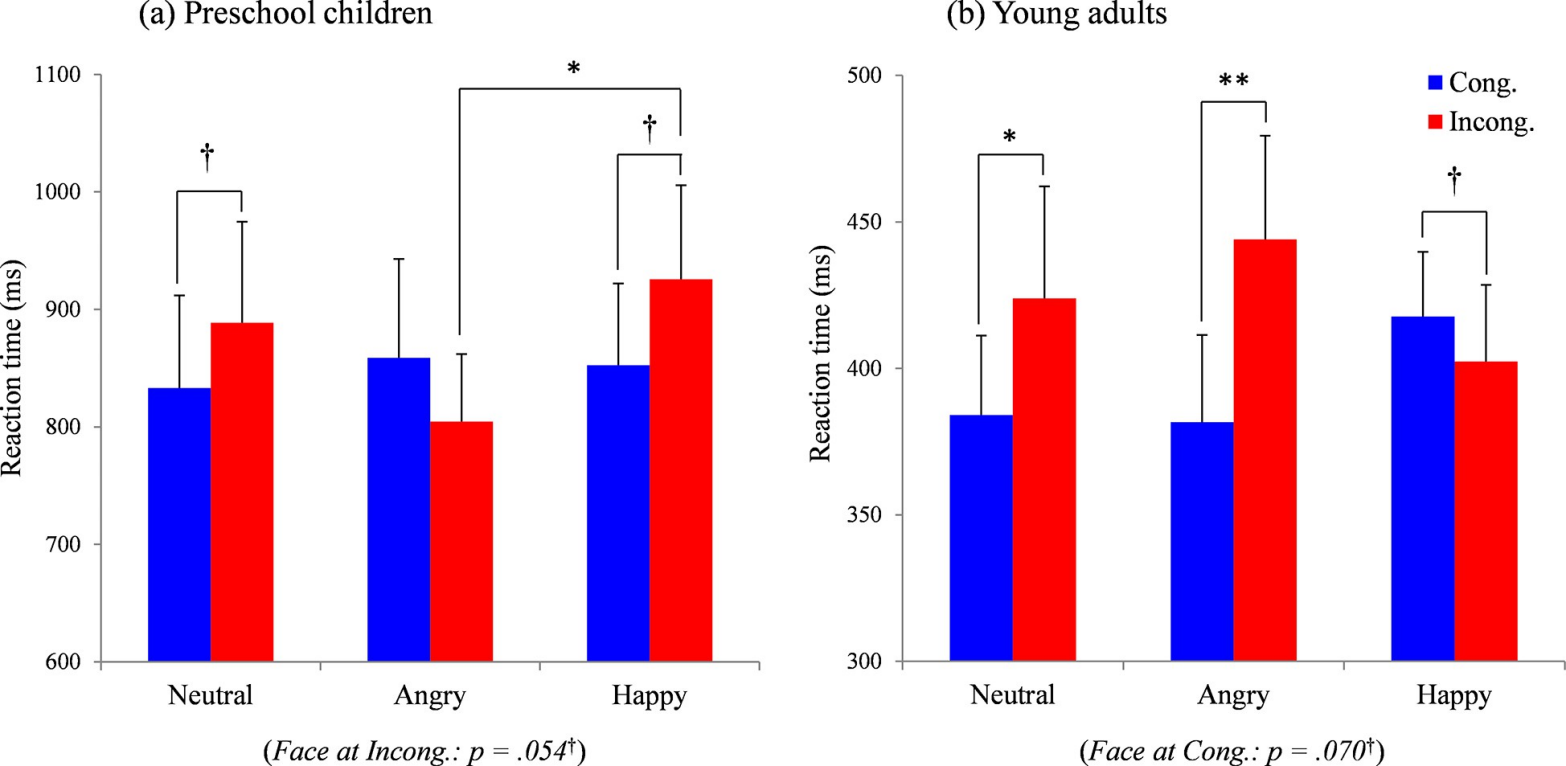
Reaction times under all experimental conditions for (a) preschoolers and (b) young adults in the behavioral study. **, *p* < .01; *, *p* < .05; ^†^, *p* < .1.

Fig 5 indicates the reaction times of the (a) preschool children and (b) young adults under all conditions of the behavioral study (S6 File). For the preschool children, the ANOVA [Table 6(A)] showed an interaction between the two factors (*p* = .032). For neutral and happy faces [Fig 5(A)], the reaction times had a tendency toward being more delayed under the incongruent condition than the congruent condition (simple main effects of Gaze, *p* = .057^†^ for neutral faces and *p* = .076^†^ for happy faces). However, these congruency effects (i.e., incongruent minus congruent reaction times) for gaze direction did not appear for angry faces. For the Face factor under the incongruent gaze, the simple main effect was marginally significant (*p* = .054^†^), followed by a significant difference between angry and happy faces ($\hat{p}$ = .038). Supplementary S7 Table presents detailed information for the tests performed after the ANOVA.

**Table 6:** Reaction times (in ms) and statistics under all experimental conditions for (a) preschoolers and (b) young adults.

Items	*F* (a) [*F*_*(2*,*20)*_ *in Face*, *F*_*(1*,*10)*_ *in Gaze*, *and F*_*(2*,*20)*_ *in Int*.] (b) [*F*_*(2*,*24)*_ *in Face*, *F*_*(1*,*12)*_ *in Gaze*, *and F*_*(2*,*24)*_ *in Int*.]		*P*	*η*_*p*_^2^ (partial *η*^2^)	Neutral		Angry		Happy	
					Cong.	Incong.	Cong.	Incong.	Cong.	Incong.
(a) Preschool children	*Face*	.867	.436	.080	833.1±78.8	888.6±86.1	858.9±83.9	804.5±57.4	852.5±69.7	925.6±80.2
	*Gaze*	1.161	.307	.104						
	*Int*.	4.124	.032*	.292						
(b) Young adults	*Face*	.160	.853	.013	384.1±27.1	423.9±38.2	381.6±29.8	444.1±35.4	417.7±22.0	402.4±26.2
	*Gaze*	9.548	.009**	.443						
	*Int*.	10.476	< .001**	.466						

**, *p* < .01

*, *p* < .05.

For the young adults, the ANOVA showed a main effect of Gaze (*p* = .009) and an interaction between the two factors (*p* < .001) [Table 6(B)]. The congruency effects (i.e., a shorter reaction times in the congruent condition or longer reaction times in the incongruent condition) were significant under the congruent and incongruent conditions for neutral (*p* = .035) and angry (*p* = .001) faces [Fig 5(B)]. By contrast, the reaction times for happy faces demonstrated a tendency toward being faster in the incongruent condition than in the congruent condition (*p* = .072^†^). The simple main effect was marginally significant for Face under the congruent condition (*p* = .070^†^), followed by no significant differences between facial expressions. Supplementary S8 Table presents detailed information on the tests performed after the ANOVA.

#### 3.2.2 Accuracy

Regardless of the presented images (three types of facial expressions and two gaze directions), the average values of the correct answer for all tasks were almost 100%, meaning the tasks were easy, even for the preschool children: 98.1 ± 0.6% for children and 100% for adults (i.e., no mistakes), resulting in *p* < .001 and *r* = .296 according to the Mann-Whitney U test. For the children, the percent correct scores were 98.9 ± 1.1% and 98.9 ± 1.1% in the congruent and incongruent gaze directions of neutral faces, 98.9 ± 1.1% and 96.6 ± 1.8% in those of angry faces, and 96.4 ± 1.8% and 98.9 ± 1.1% in those of happy faces.

### 3.3 Children versus young adults

Overall, the preschool group had large central and parietal P3 responses on trials with emotional faces. The young adult group had a large frontal N2 response to angry faces, and their parietal P3 responses to happy faces were smaller on congruent trials and greater on incongruent trials. For the preschoolers, the Pm*α* power to change attention levels was inhibited for angry faces during the encoding and latter retention periods of a target. For the young adults, Fm*θ* power increased and Pm*α* power attenuated, especially during the encoding period. During the first retention period, Fm*θ* power increased under the incongruent gaze for happy faces, and Pm*α* power increased under the congruent gaze for happy faces. In the behavioral study, no congruency effects or reversed gaze cueing effects were observed for angry faces in the preschool group or for happy faces in the adult group.

The primary purpose of this study was to use a within-subjects design to investigate the effect of experimental conditions on the features of and differences in the brain activity of developing preschool children. A within-subjects design was also used to analyze data in young adult participants to investigate the brain dynamics that occur when developing children reach mature levels. It is generally difficult to compare evaluation indices of children to those of adults in a simple and direct manner because brain mechanisms, such as the location and latency for brain activities, seem to differ across ages [38,75,76]. However, we compared the differences between the children and adults to further understand the results of our EEG and behavioral studies. The main interest of our analysis was the difference between the two groups under the same experimental conditions for facial expressions and gaze direction. The evaluation indices and experimental conditions that differed significantly between the two groups corresponded to the immature brain activity of developing children.

Table 7 summarizes the statistical significance between the children and adults for the ERPs, wavelet analysis, and reaction times for each condition (S2−S6 File). Based on the characteristic differences between the ERPs of children and adults, we added the N1 at Pz (minimum value between 150 and 280 ms for children, 90 and 150 ms for adults) and the N2 at Cz (minimum value between 300 and 500 ms for children, 200 and 360 ms for adults) to the definition of ERPs. Overall, the ERPs of the children were similar to those of the adults. However, we observed a sharp response for the adults and a slower response for the children. For the central and parietal sites, remarkable differences appeared between the children and adults: N2 modulation at Cz for the adults and a greater N1 response at Pz for the children. The central and parietal P3 responses significantly increased under the congruent gaze condition for angry faces in the children than in the adults.

**Table 7:** Preschool children versus young adults in evaluation indices.

Items	*Χ* ^*2*^ ^(df = 11)^	*P*	*ε* ^2^	Neutral		Angry		Happy	
				Cong.	Incong.	Cong.	Incong.	Cong.	Incong.
N1 at Fz	82.4	< .001**	.406	.086^†^ (-6.0)	.015* (-7.8)	.115 (-5.2)	.024* (-6.9)	.002** (-8.8)	.005* (-7.8)
P2 at Fz	33.8	< .001**	.166	.559 (+3.9)	.024* (+4.4)	.794 (+3.4)	.984 (+1.8)	.411 (+4.2)	.774 (+4.5)
N2 at Fz	77.2	< .001**	.380	.030* (-6.6)	.010* (-7.7)	.042* (-6.1)	.021* (-7.0)	.047* (-6.0)	.013* (-7.2)
Late at Fz	9.4	.583	.046	1.000 (0.0)	1.000 (+0.1)	.999 (+1.8)	.459 (+2.3)	1.000 (0.0)	1.000 (0.0)
N2 at Cz	80.5	< .001**	.397	.004** (-7.2)	.019* (-5.8)	.002** (-7.2)	.071^†^ (-6.1)	.047* (-5.8)	.086^†^ (-5.5)
P3 at Cz	36.4	< .001**	.179	0.958 (+1.3)	1.000 (+1.5)	.013* (+5.7)	.224 (+3.2)	.814 (+2.3)	1.000 (+1.0)
N1 at Pz	73.2	< .001**	.360	.027* (-7.4)	.027* (-6.7)	.058^†^ (-6.3)	.010* (-6.1)	.071^†^ (-7.7)	.042* (-6.4)
P3 at Pz	28.7	.003**	.141	1.000 (-1.7)	1.000 (+0.4)	.017* (+6.2)	.242 (+3.9)	.774 (+4.0)	1.000 (-0.1)
Fm*θ*	(1) Enc. 15.4	.163	.076	1.000 (-9.9)	.192 (+38.1)	1.000 (+5.4)	.999 (+12.6)	1.000 (-2.5)	1.000 (-27.1)
	(2) Ret.1 15.1	.178	.074	1.000 (+0.1)	.814 (+22.9)	1.000 (-18.1)	1.000 (-4.5)	1.000 (-1.3)	.992 (-25.3)
	(3) Ret.2 10.5	.486	.052	1.000 (+2.4)	.987 (+16.7)	1.000 (+4.5)	.997 (+17.6)	1.000 (+2.6)	1.000 (-4.6)
Pm*α*	(1) Enc. 27.9	.003**	.138	.987 (+15.5)	.192 (+26.8)	1.000 (+7.4)	.996 (+10.4)	.997 (+15.0)	.115 (+34.7)
	(2) Ret.1 16.5	.122	.082	1.000 (-1.9)	.999 (+18.2)	1.000 (-4.7)	1.000 (-5.6)	.950 (-18.5)	.990 (+14.3)
	(3) Ret.2 12.3	.340	.061	1.000 (-9.5)	.980 (+28.7)	1.000 (-6.0)	.909 (-15.4)	1.000 (-4.3)	.994 (+18.9)
Reaction time	98.6	< .001**	.689	.005** (+449.0)	.007** (+464.7)	.007** (+477.3)	.010* (+360.4)	.004** (+434.8)	.003** (+523.2)

(), difference: Children minus adults.

**, *p* < .01

*, *p* < .05

†, *p* < .1.

Enc., encoding; Ret.1, first retention; Ret. 2, latter retention.

For the wavelet-based data, the Kruskal-Wallis test clarified that there was a significant main effect (*p* = .003) between all conditions for the two groups in the Pm*α* powers during the encoding period, followed by no differences between the two groups under the experimental conditions (see Supplementary S9 Table for details). For the reaction times, the Kruskal-Wallis test showed a significant main effect (*p* < .001), followed by the multiple comparisons (i.e., the Steel-Dwass test): the reaction times of all cases under the same experimental conditions were significantly delayed for the children than for the adults (see S9 Table for details).

## 4. Discussion

This study investigated the brain dynamics of attention-shifting in preschoolers under facial expression and gaze interaction conditions. Participants were required to detect and memorize the location of a target as a simple working memory task while ignoring or facilitating gaze direction and emotional expressions. We hypothesized that developing children would present different brain activities compared to adults, such as changing ERPs and time-frequency powers related to working memory as well as distributing attention. We also expected that the effects of facial expression and gaze interaction would be evident in the congruency effects in both preschoolers and young adults, although the responses to incongruent stimuli are generally complicated in developing preschoolers. Regarding the simultaneous presentation of a target and obstacles or attractive information for this study (i.e., gaze direction and facial expressions in SOA = 0 ms, no priming tasks), we predicted that preschoolers would exhibit irregular brain and behavioral responses compared to the more mature young adults.

Before discussing the results, we would like to note that some experimental limitations exist for this study. We performed the EEG study as a priority and then carried out the behavioral study separately to estimate reaction times and accuracy during EEG recordings. We performed the EEG first and the behavioral experiment second, although the adult participants completed both tasks on the same day. The order of the experiments may explain a bias of experimental design in the form of repeated monotonous work and task-induced fatigue effects [77], in addition to the familiarity effect and the transfer effect. Because all participants completed the experiments after sufficient practice, the transfer effect between the required tasks would have been minimized. It is also considered that there were no significant effects because the task was too easy (i.e., simple responses by pressing a left or right button corresponding to the target location). Furthermore, the fewer number of EEG trials for the children might have affected the signal noise reduction (e.g., the amplitudes and variances of ERPs and the frequency powers) compared to that for adults. To suppress the differences between the groups, this study was based on a within-subjects design, and we applied nonparametric tests to compare the children with adults. Future studies should develop optimal methods for comparing different age groups and sufficiently consider the appropriate values of the effect size and sample size based on our results.

### 4.1 ERPs

The maturation of cognitive abilities generally results in reduced amplitudes, shorter latencies, and different topographic areas of ERPs [38,75] and these differences are presumably caused by various factors, such as brain size, skull thickening, and synaptic density [76]. Early ERPs, such as N1 and P2, can achieve a mature state in childhood [78], although the amplitudes tend to be greater with a delayed latency in children than adults [38,75]. The parietal N1 deflection found in this study was significant for children, but not adults (Fig 2 and Table 7). This result suggests that the attention for initially perceiving faces is greater, requiring more effort in early childhood than in a mature stage because the parietal N1 deflection reflects a general characteristic of the spatial focusing of attention [34].

The central and parietal P3 responses in preschoolers are suggestive of brain activity associated with perceiving angry faces because of the counterbalanced difference of reaction times (i.e., no congruency effects) in the behavioral study. Regarding the reaction times for preschoolers, the Gaze condition for angry faces was not statistically significant (i.e., *p* = .237 for the simple main effect after the interaction of ANOVA, see details in Supplementary S7 Table). Accordingly, this result may be due to reversed cueing effects between the congruent and incongruent gaze conditions for angry faces in preschoolers. In addition, the tasks for this study may not strictly correspond to general priming tasks (i.e., a cue indicator before presenting a target) because of the simultaneous target presentation. The central and parietal P3 responses commonly resulted in statistically significant differences between children and adults only for the congruent gaze condition for angry faces (Table 7). These results indicate that undeveloped (e.g., anger control including gaze information, compared to adults) or developing (e.g., high sensitivity such as feeling potential risk and paying attention to the congruent gaze direction for angry faces) brain activity might still exist in children to allow them to judge the congruent gaze for angry faces. This feature for children could disappear in young adults as they mature, and it could also cause the delayed reaction time in the congruent gaze for angry faces, linked also to the lack of congruency effects for the behavioral task. Because the characteristics of the EEG and behavioral responses were complicated, especially for the preschoolers, further investigations should be done to clarify the brain mechanisms and functions we were not able to investigate.

The young adult participants might have recognized the angry faces instantaneously or covertly, owing to the greater change of frontal N2 deflection (Table 3) generated primarily from the prefrontal cortex and ACC [41]. Nevertheless, it may not have affected the reaction times (e.g., no faster or delayed responses between facial expressions, as shown in Fig 5). Gaze and facial expression processing could be independent during the early stages and integrate later (around 300 ms), possibly at the fusiform gyrus or superior temporal gyrus [54]. The simple process of reacting to a target (i.e., no priming paradigm) might have been completed in parallel with facial detection because of the sufficient working memory capacity of adults, whereas the gaze cueing tasks with higher loads showed a clear difference, even between neutral and angry faces [79]. Regardless of the specific effects of angry faces on early brain activity (the activated prefrontal cortex and ACC activities based on the observed frontal N2 deflection), the reaction time results showed similar congruency effects for angry and neutral faces (Fig 5). It is difficult to compare neutral and angry faces directly for the same condition in this study because most previous studies have been based on priming paradigms (i.e., no simultaneous appearance of a target with an emotional face and gaze). However, more difficult tasks might trigger significant differences between neutral and angry faces with gaze [79] because of exceeding the use of early brain activity, resulting in higher sensitivity to angry faces. On the other hand, the overall N2 amplitudes at the frontal and central sites were greater in children than in adults (Table 7), suggesting that the N2 deflection gradually decreases throughout developmental stages, but this effect can be modulated by detecting angry faces at a mature stage without a specific influence on reaction times.

The modulation in later ERPs (such as P3) associated with higher cognitive processes continue to mature until young adulthood [78]. For the preschool children, rather than early ERPs, the frontal late positive response and the central and parietal P3 responses were greater for angry faces than for the other faces [Fig 2(A) and Table 2], presumably reflecting an efficient shifting of attention caused by negative emotional stimuli and the updating of working memory during the task. For the adults, the parietal P3 response was greater for the incongruent gaze of happy faces, the amplitude of which was almost consistent with the congruent gaze of neutral faces [Fig 2(B) and Table 3]. This result suggests that adequate attention was allocated to detecting a target while ignoring the incongruent gaze, resulting in an easier attention shift for happy faces, and previous reports support this finding [23,80–82]. For example, the parietal P3 response for adults was more increased for positive faces than for negative faces [80]. Reaction time and accuracy were also improved for happy faces, which is known as the happy face superiority effect [23,81]. Interestingly, such effects in adults might cause a greater P3 response during the selection of a target while ignoring the incongruent gaze for happy faces, and the P3 responses found in this study were observed close in time to the reactions (i.e., pressing a button). In contrast to the fact that earlier brain processing can affect faster detection of angry faces than happy faces, Calvo and Nummenmaa [83] found shorter first fixations and dwell times for happy faces than for negative faces, implying the involvement of later brain processing as a happy face advantage. On the basis of this evidence, we can speculate that the greater P3 response in this study might be partly related to later attentional processing for happy faces (perhaps as evidence of a cognitive process rather than perception), although its direct physiological mechanisms remain unknown.

### 4.2 Time-frequency responses

#### 4.2.1 Fmθ power

The Fm*θ* power for the preschoolers was significantly increased in the incongruent gaze condition compared with the congruent gaze condition during the encoding and retention periods and in neutral faces compared with emotional faces (Fig 3). This result suggests that the preschool children might have needed strong attention/concentration in the incongruent gaze condition for neutral faces. The ability to avoid irrelevant distractors grows rapidly in developing children [84], and in this study it was well activated, even in the preschool children aged around 5 years. In mature stages, people process emotional facial recognition and gaze direction in parallel [54]. However, it may be difficult for developing preschool children to quickly recognize gaze direction in combination with emotional facial expressions. This may explain the similarity of Fm*θ* power among emotional faces, implying that Pm*α* power is required to pay attention to them.

For the young adults, during the encoding and first retention periods, the Fm*θ* power for happy faces (especially in the incongruent condition) was greater than that for neutral and angry faces (Fig 4). This result means that the adults paid greater attention to happy faces under the incongruent gaze, probably inducing faster reaction times [i.e., no congruency effect in Fig 5(B)]. Usually, the human cognitive system strongly prioritizes happy faces (i.e., the happy face superiority effect). In fact, happy faces can facilitate a quick and accurate response in behavioral studies, compared to other emotional faces [23]. The modulated Fm*θ* power for this study could be one of the unknown physiological mechanisms for ignoring the incongruent gaze effectively for happy faces, as well as recognizing it quickly. Previous reports also indicated that Fm*θ* power increases under positive conditions [85,86], suggesting that emotional processing reflects attentional functions. However, in the preschool children, the happy face superiority effect was not significant in the behavioral and EEG (Fm*θ* and Pm*α*) indices (Table 7), and it is possible that undeveloped brain abilities are related to superior recognition performance for happy faces.

#### 4.2.2 Pmα power

For the preschool children, Pm*α* power, rather than an increase of Fm*θ* power, significantly decreased during the encoding period for angry faces (Fig 3), showing that the brain function was modulated by emotional stimuli [55]. Because subjective reports of emotional arousal are directly correlated with reduced *α* power mainly at the parietal site [87], the inhibited Pm*α* power for angry faces in this task implies a high arousal stimulation for the preschool children. In contrast, happy faces may cause low arousal in preschool children, compared to angry faces. Increased emotional arousal might have also affected the faster reaction times, even in the incongruent gaze for angry faces (i.e., no congruency effect in Fig 5). In addition to experiencing negative feelings caused by the angry faces, the preschool children may have easily detected targets while ignoring distractors, shifting their attention to the opposite side away from the averted gaze direction of an angry face [88]. Moreover, Pm*α* power was activated during retention periods that rely on working memory loads [64], but in this study, the Pm*α* power for angry faces was significantly inhibited, even during the latter retention period (Fig 3), suggesting that the preschool children dwelled on negative emotions for a longer period.

For the young adults, Pm*α* power increased linearly throughout the encoding and retention periods for each condition (Fig 4). This result supports previous findings regarding activated Pm*α* power depending on working memory loads during a retention period [64]. Interestingly, the Pm*α* power during the encoding period might have been inhibited specifically in the incongruent gaze direction of happy faces (Fig 4). It is suggested that Pm*α* inhibition is associated with switching operations, encoding, and decision-making [55,63] rather than simple memory maintenance. Adults may be able to smoothly ignore the incongruent gaze direction of happy faces and shift their attention to a target on the opposite side, using the effect of *α* inhibition. By contrast, *α* inhibition in the preschool children was observed for angry faces (Fig 3), meaning an increased attention/concentration level was required to complete the task. This could imply the possibility of paying attention to their parents’ angry faces in everyday life.

#### 4.2.3 Interaction of Fmθ and Pmα

We observed effects of facial expression and gaze interaction, which significantly changed the Fm*θ* and Pm*α* powers for children and adults (Figs 3 and 4). During the selection of a target, the neuronal dynamics involve emotional facial processing as well as eye-gaze attention [55], which may be activated through hybrid (or parallel and complicatedly distributed) brain pathways around the frontal and parietal regions. In particular, the source of Fm*θ* power can be identified as the medial prefrontal cortices with ACC [58], and attention control for monitoring performance in incongruent trials could activate the ACC [89]. Happy as well as angry faces can enhance neural networks within the amygdala and ACC [90,91], and emotional face processing can positively elicit the amygdala-ACC connectivity in an anxiety group [92]. Therefore, the ACC may be a key component connecting emotion processing with attentional control for working memory tasks.

Moreover, increased Fm*θ* power is correlated with inhibited Pm*α* power during encoding [63] and working memory operations [93]. In contrast, the frontal and parietal *α* powers simultaneously increase during memory retention for a target [64]. These interactive signs were recognized in the adults in this study, especially under the incongruent gaze for happy faces. Although brain activities in the ACC and DLPFC can improve during early childhood [65], the interactive mechanism generating *θ* and *α* rhythms in preschoolers remains unknown [66]. Further investigation could reveal the relationships between aging and the induced Fm*θ* and Pm*α* responses associated with the cognitive processes of developing children.

### 4.3 Behavioral study

Pecchinenda and Petrucci found gaze cueing effects for happy faces in 6‒7-year-old children, but not for neutral or angry faces in the form of faster and more accurate responses to validly cued targets (Table 2 and Fig 2 in [29]). In our behavioral study, gaze congruency effects (reaction times) were observed for happy and neutral faces in the preschoolers. These results indicate that younger children experience more delayed responses to the incongruent gaze than the congruent gaze for happy faces, although this effect may be small. Given that young children, and even infants, pay attention to happy faces and tend to look away from threat-related angry faces, it seems that young children prefer happy faces to angry ones [94–96]. Therefore, it may be easier to develop brain patterns that process happy faces more quickly and appropriately than angry faces. In this study, either reversed or no congruency effects existed for happy faces and gaze in the young adults, which may indicate a happy face effect. Because the brain and behavioral results greatly depend on the age of developing children, task difficulty, and task type, including different SOAs (e.g., [25,27,28]), the age of the preschoolers in our study might have introduced variance from individual differences and growth rates.

The preschoolers in this study demonstrated modulations of brain and behavioral responses for angry faces and gaze. The longer response latencies in validly cued trials with angry faces than in invalidly cued trials (i.e., the opposite of a gaze congruency effect or no effect) suggest that the young children required more time to disengage their attention than the adults from the gaze cue location (i.e., the center on a screen) for angry faces. Orienting to gaze cues (i.e., response to the target) requires recognizing gaze direction, disengaging attention from the cued location, and redirecting it [97]. In fact, our results are supported by the findings of Pecchinenda and Petrucci [29] that show a lack of gaze cueing effect for angry faces in young children. However, those results may be modulated by the duration of SOAs, as well as the age differences in emotion enhanced gaze-cueing effects. The only difference between our results and this previous study [29] (i.e., gaze congruency effects and no effects in neutral faces) seems to be induced by slightly different ages of children and the duration of SOAs.

We speculate that developing children might be more sensitive to angry faces than those in more mature stages. Although our results did not measure a questionnaire survey to categorize anxiety, a previous study [26] found that congruency effects disappeared with high anxiety. Our experiments were performed after the children became familiar with the experimenters and experimental circumstances, and the parents were sitting near them during the experiment. However, the children might have felt a little anxious, which could have possibly induced more sensitive brain responses to negative emotional stimuli. We consider it possible that such anxiety affected the results, especially in the attention-shifting from the angry face condition to a target. It is easy for a subject to recognize angry faces with direct gazes, and it is difficult to shift attention to a target. However, angry faces with averted gazes become ambiguous, as shown by a previous study [88]. Therefore, in our study, the preschool children observing an averted gaze could have easily switched their attention to a target, quickly ignoring the incongruent gaze in an angry face, a result that falls in line with the early selection theory of attention. The preschoolers for this study might have also felt negative emotions (induced valence arousal) in the congruent gaze for angry faces, inducing a delayed response. Conversely, angry faces with the incongruent gaze might easily facilitate release from a negative feeling to the gaze direction, allowing the participant to focus on the opposite side with a target. It is possible that the interaction between those characteristics’ effects could result in no congruency effects for angry faces. By contrast, the low anxiety and mature developmental stage of the young adults would not have reflected congruency effects for angry faces.

Compared to young adults, preschoolers present different type of sensitivity in brain processing and behavioral responses to angry faces, and this difference might be caused by an anatomically undeveloped connection between the amygdala and prefrontal cortex [98]. Interestingly, amygdala sensitivity increases in response to angry expressions in children between the ages of 3 and 8 years [99]. This anatomical difference in young children can induce complicated responses, even in congruent and incongruent tasks. Furthermore, because preschoolers tend to be sensitive to angry faces, they may pay excessive attention to a target direction. This condition might easily cause them to perceive the location of an angry face’s gaze as dangerous, which can produce a quick inverted response toward the gaze’s opposite direction to inhibit negative information, such as danger or threat (i.e., the inhibition of return [31]). Conversely, a delayed response can be induced in congruent gaze conditions for angry faces. Using oculomotor recording in young children to detect the inhibition of return [30] could clarify the differences between orienting and disengaging in the gaze-cueing effect with emotional faces.

### 4.4 Perspectives

The working memory task in this study was easy for both the children and adults, although there were some differences in brain activity. More difficult working memory tasks and distractors could create more significant differences between the conditions and groups. In addition, brain and behavioral responses during working memory tasks depend on the length of stimulus presentation [25], the strategy to solve tasks efficiently (e.g., focusing only on a target, ignoring face and gaze direction), and the gender of presented faces [100]. In our study, the stimuli presentation time was longer (1 s) than in previous studies [25,27]. Furthermore, all the stimuli (i.e., target, facial expression, and gaze direction) were presented at the same time, meaning the study employed overt stimuli, not covert or priming ones. In this case, the developing preschoolers might not have been able to detect facial and gazing cues simultaneously, compared to priming stimuli to engage covert attention before target appearance. In addition, the extra stimuli (e.g., angry faces and gaze) as distractors may not have been able to influence reaction time, even under the congruent gaze, because of the proximity to a target that simply comes into view, as would a covert attention condition that implies target location. Conversely, it might be easy for preschoolers to ignore the incongruent gaze in angry faces. If the stimulus presentation length is shorter, the presented stimuli are primed to induce covert attention, and if moving facial expressions and gaze direction are used [25,28,101], the brain and behavioral activities may be more enhanced.

When a target stimulus and a facial expression with a congruent or incongruent gaze are presented simultaneously, participants are required to judge and memorize a target location quickly while ignoring or removing irrelevant distractors. Working memory capacity clearly differs between children and adults, and it is generally lower in preschool children [38]. In addition, a high load of frontal ‘cognitive’ control processes (e.g., memorizing numbers) increases distractor processing (i.e., late selection theory of attention) [102]. In contrast, a high perceptual load can allow task-irrelevant distractors to be ignored during a character search task (i.e., early selection theory of attention), even under the incongruent conditions [103]. It may, therefore, be impossible for developing children to perceive all the information (emotional faces and gaze) instantaneously and simultaneously compared to adults, who have sufficient working memory capacity. In our study, the preschool children might have been able to ignore incongruent gaze shifts (conversely, a delayed response in the congruent condition) for angry faces because of the high amount of perceptual information, immediately after stimuli presentation. Here, the question remains why such an effect was significant in angry faces. In general, angry faces can quickly stimulate amygdala activity under unconscious and covert conditions [91]. On the other hand, detecting a target might use another recognition process, and the task-irrelevant information may be rejected in the early response at a sensory level. Furthermore, negative emotional arousal can modulate the prefrontal cortex and pain network/pain matrix activity [104], and angry face perception can trigger empathy for pain‑related dorsolateral prefrontal activity [105]. Thus, for the preschoolers in this study, specific brain mechanisms of emotional pain and distress might have been associated with a lack of congruency effects between gaze conditions in angry faces.

We should also consider age-related changes and specific brain processing related to early childhood. For example, 6-year-old children successfully inhibited a distractor (i.e., a bias away from a distractor), but 3-year-old children showed a bias toward a distractor [84]. Accordingly, preschool children, even those around 5 years of age, may have the ability to ignore incongruent gaze shifts in angry faces upon first perception. Moreover, greater gaze-cueing effects were found in 9 to 10-year-old children for angry faces than for neutral faces, but for 6–7-year-old children, this result occurred only for happy faces [29], which supports the results of our behavioral study (i.e., no congruency effect for angry faces). This evidence means that emotion-enhanced gaze-cueing effects exist in older children.

Preschoolers in our study were not able to shift attention from obstacles while inducing negative arousal (i.e., angry faces and an inverted gaze cue). From a social-cognitive perspective, it is crucial for children to infer others’ anger and gaze so that they can control emotions (e.g., anger management), build and preserve good human relations in domestic, school, and social circumstances, and lead fulfilling social lives. However, developing children may not yet have the brain ability to process angry faces inducing negative arousal and attention shift, which is supported by our results and previous research [29]. In addition to the young adults in our study, 9–10-year-old children have been shown to demonstrate a gaze congruency effect for angry faces [29], and amygdala sensitivity to angry expressions can increase between the ages of 3 and 8 years [99]. Therefore, preschoolers and younger elementary students seem to be at a boundary or age for developing such processing abilities. The different responses between preschoolers and adults imply a necessary key part of growth in developing brain activity. Based on our results, developing children may not be able to precisely sense their parents’ or educators’ gazes and angry faces.

## 5. Conclusions

We investigated preschool children’s attention abilities during a working memory task with facial expressions and gaze, resulting in the modulation of brain dynamics and reaction times with those of young adults. For the preschool children, angry faces activated later ERPs at midline regions and mainly inhibited Pm*α* power, rather than Fm*θ* power, reflecting emotional attention levels. Such brain activities may not induce congruency effects for angry faces because of the delayed and faster reaction times under the congruent and incongruent gazes, respectively. For the young adults, the Fm*θ* and Pm*α* powers as well as the parietal P3 amplitude changed in the incongruent gaze for happy faces, resulting in a tendency for shortened reaction times (i.e., no congruency effect). These results suggest that adults at a mature stage can sufficiently allocate and shift their attention to a target for happy faces while ignoring distractors, and this may be facilitated by the happy face superiority effect. In contrast, developing preschool children have not yet developed such abilities. By quantifying and visualizing the characteristics of brain dynamics in preschool children, we may be able to discover a learning delay and design a learning and training method for increasing attention/concentration levels.

## Supporting information

S1 Fig (TIF)

S1 Table ERPs for preschoolers. (a) Latency of N2 at Fz: Simple main effect test after the interaction of ANOVA. (b) Late response at Fz: Multiple comparisons between Face conditions. (c) P3 at Cz: Multiple comparisons between Face conditions. (d) P3 at Pz: Multiple comparisons between Face conditions.
(PPTX)

S2 Table ERPs for young adults. (a) N1 at Fz: Simple main effect test after the interaction of ANOVA. (b) Latency of P2 at Fz: Simple main effect test after the interaction of ANOVA. (c) Latency of P2 at Fz: Multiple comparisons between Face conditions at Cong. (d) N2 at Fz: Multiple comparisons between Face conditions. (e) P3 at Pz: Multiple comparisons between Face conditions. (f) P3 at Pz: Simple main effect test after the interaction of ANOVA. (g) P3 at Pz: Multiple comparisons between Face conditions at Cong. (h) P3 at Pz: Multiple comparisons between Face conditions at Incong.
(PPTX)

S3 Table Steel’s test results for preschoolers. (PPTX)

S4 Table Wavelet analysis for preschoolers. (a) The encoding period for Fm*θ* power: Simple main effect test after the interaction of ANOVA. (b) The encoding period for Pm*α* power: Multiple comparisons of Face conditions. (c) The latter retention period for Pm*α* power: Multiple comparisons between Face conditions.
(PPTX)

S5 Table Steel’s test results for young adults. (PPTX)

S6 Table Wavelet analysis for young adults. (a) The first retention period for Fm*θ* power: Multiple comparisons between Face conditions. (b) The first retention period for Fm*θ* power: Simple main effect test after the interaction of ANOVA. (c) The first retention period for Fm*θ* power: Multiple comparisons between Face conditions at Incong. (d) The first retention period for Pm*α* power: Multiple comparisons between Face conditions.
(PPTX)

S7 Table Reaction times for children. (a) Simple main effect test after the interaction of ANOVA. (b) Multiple comparisons between Face conditions at Incong.
(PPTX)

S8 Table Reaction times for young adults. (a) Simple main effect test after the interaction of ANOVA. (b) Multiple comparisons between Face conditions at Cong.
(PPTX)

S9 Table All *p* values for the Steel-Dwass comparisons for each index between children and adults. (PPTX)

S1 File Raw CBQ data. Table 1.
(ZIP)

S2 File Raw EEG data at Fz, Cz, and Pz for preschoolers. Fig 2(A), Tables 2 and 7.
(ZIP)

S3 File Raw EEG data at Fz, Cz, and Pz for young adults. Fig 2(B), Tables 3 and 7.
(ZIP)

S4 File Wavelet data at Fz and Pz sites for preschoolers. Fig 3, Tables 4 and 7.
(ZIP)

S5 File Wavelet data at Fz and Pz sites for young adults. Fig 4, Tables 5 and 7.
(ZIP)

S6 File Behavioral data for preschoolers and young adults. Fig 5, Tables 6 and 7.
(ZIP)
