# Supplementary figures and images for: Effects of facial expression and gaze interaction on brain dynamics during a working memory task in preschool children
Source: PLoS One. 2022 Apr 28;17(4):e0266713. doi: 10.1371/journal.pone.0266713 (PMC9049575; doi:10.1371/journal.pone.0266713)

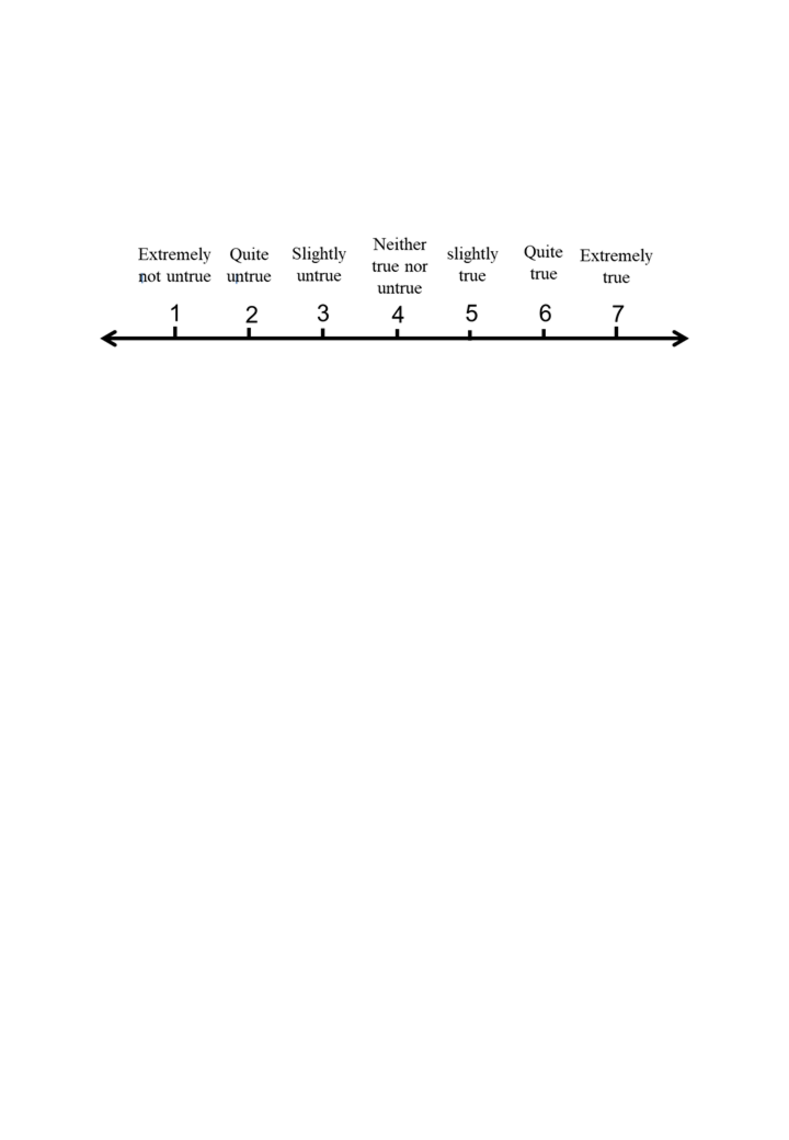

Supplement: S1 Fig — (TIF) [file pone.0266713.s001.tif]
