# Supplementary material for: Effects of facial expression and gaze interaction on brain dynamics during a working memory task in preschool children
Source: PLoS One. 2022 Apr 28;17(4):e0266713. doi: 10.1371/journal.pone.0266713 (PMC9049575; doi:10.1371/journal.pone.0266713)
Supplement: S1 Table — (a) Latency of N2 at Fz: Simple main effect test after the interaction of ANOVA. (b) Late response at Fz: Multiple comparisons between Face conditions. (c) P3 at Cz: Multiple comparisons between Face conditions. (d) P3 at Pz: Multiple comparisons between Face conditions. (PPTX) [file pone.0266713.s002.pptx]

## Slide 1
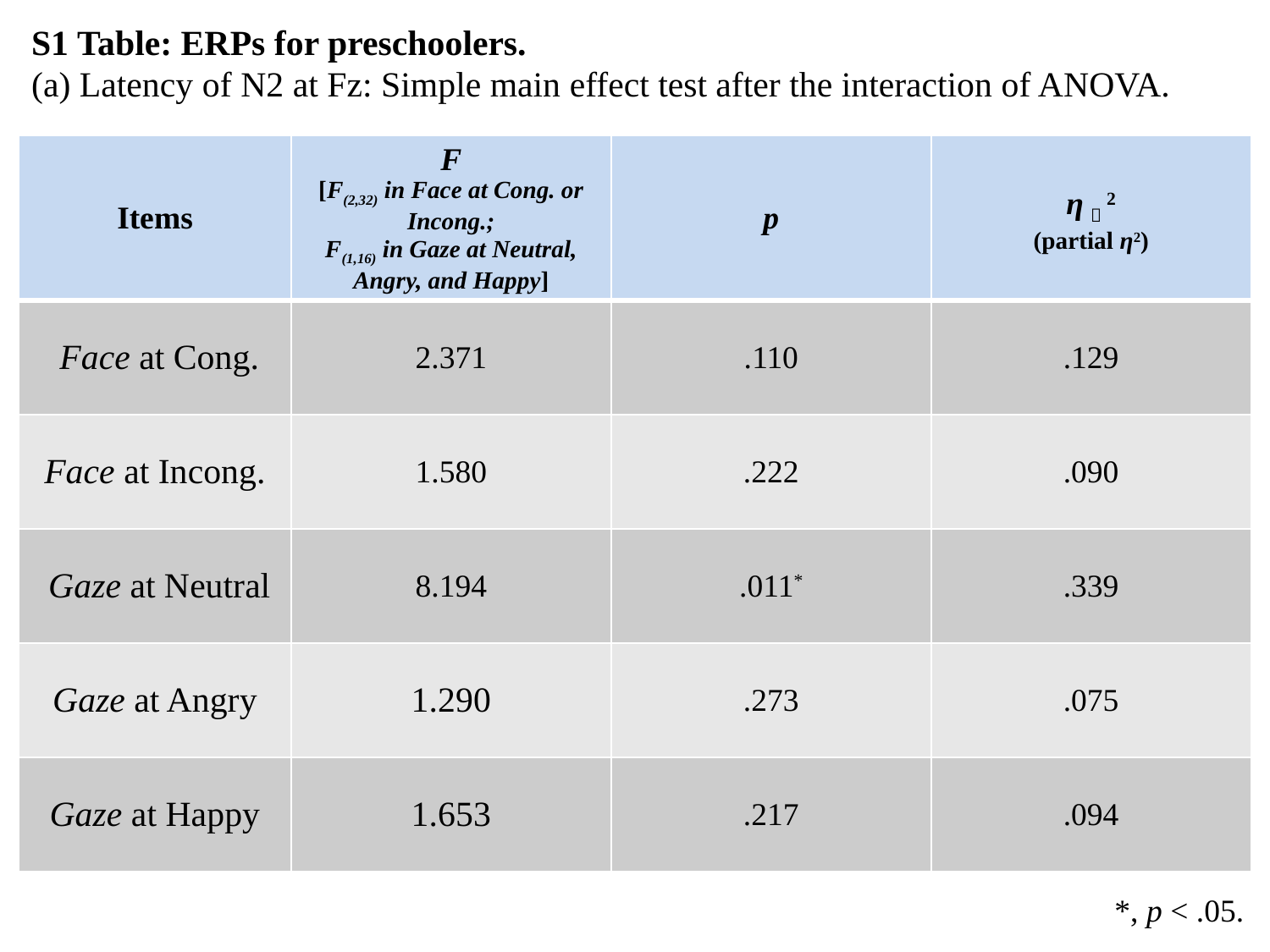

# S1 Table: ERPs for preschoolers. (a) Latency of N2 at Fz: Simple main effect test after the interaction of ANOVA.
| Items | F [F(2,32) in Face at Cong. or Incong.; F(1,16) in Gaze at Neutral, Angry, and Happy] | p | ηｐ2 (partial η2) |
| --- | --- | --- | --- |
| Face at Cong. | 2.371 | .110 | .129 |
| Face at Incong. | 1.580 | .222 | .090 |
| Gaze at Neutral | 8.194 | .011\* | .339 |
| Gaze at Angry | 1.290 | .273 | .075 |
| Gaze at Happy | 1.653 | .217 | .094 |
*, p < .05.

## Slide 2
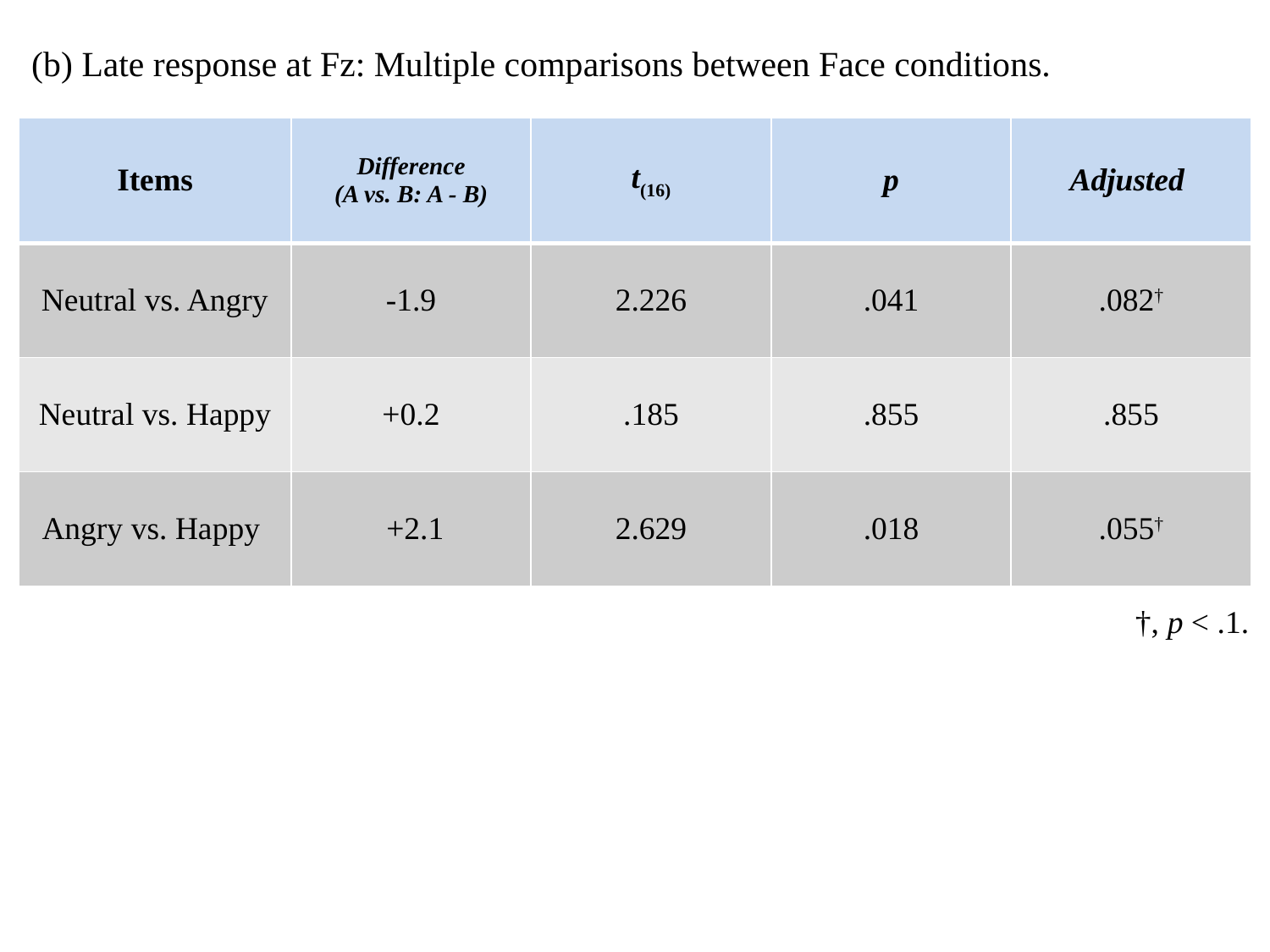

(b) Late response at Fz: Multiple comparisons between Face conditions.
†, p < .1.

## Slide 3
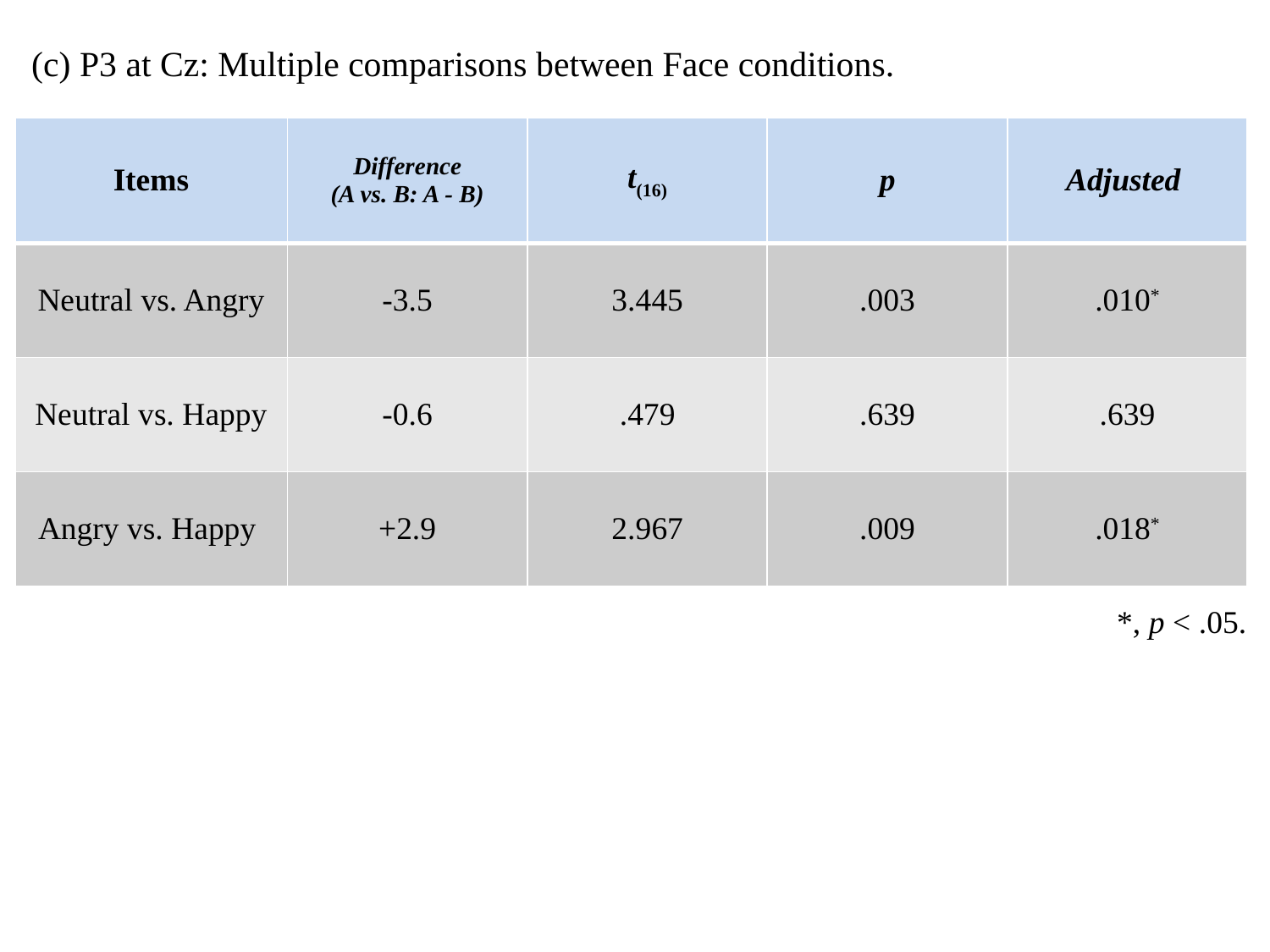

(c) P3 at Cz: Multiple comparisons between Face conditions.
*, p < .05.

## Slide 4
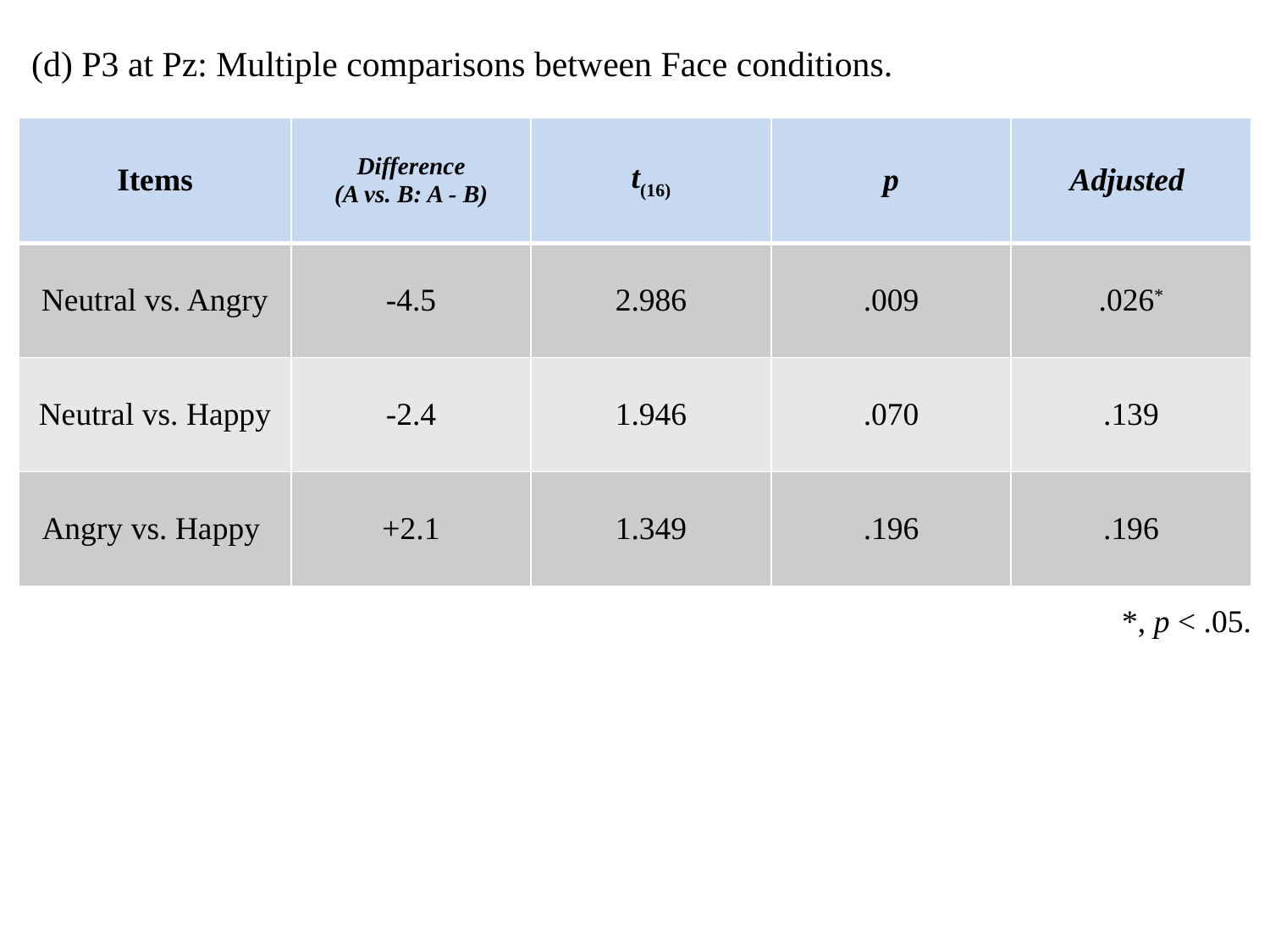

(d) P3 at Pz: Multiple comparisons between Face conditions.
*, p < .05.
