# Supplementary material for: Effects of facial expression and gaze interaction on brain dynamics during a working memory task in preschool children
Source: PLoS One. 2022 Apr 28;17(4):e0266713. doi: 10.1371/journal.pone.0266713 (PMC9049575; doi:10.1371/journal.pone.0266713)
Supplement: S2 Table — (a) N1 at Fz: Simple main effect test after the interaction of ANOVA. (b) Latency of P2 at Fz: Simple main effect test after the interaction of ANOVA. (c) Latency of P2 at Fz: Multiple comparisons between Face conditions at Cong. (d) N2 at Fz: Multiple comparisons between Face conditions. (e) P3 at Pz: Multiple comparisons between Face conditions. (f) P3 at Pz: Simple main effect test after the interaction of ANOVA. (g) P3 at Pz: Multiple comparisons between Face conditions at Cong. (h) P3 at Pz: Multiple comparisons between Face conditions at Incong. (PPTX) [file pone.0266713.s003.pptx]

## Slide 1
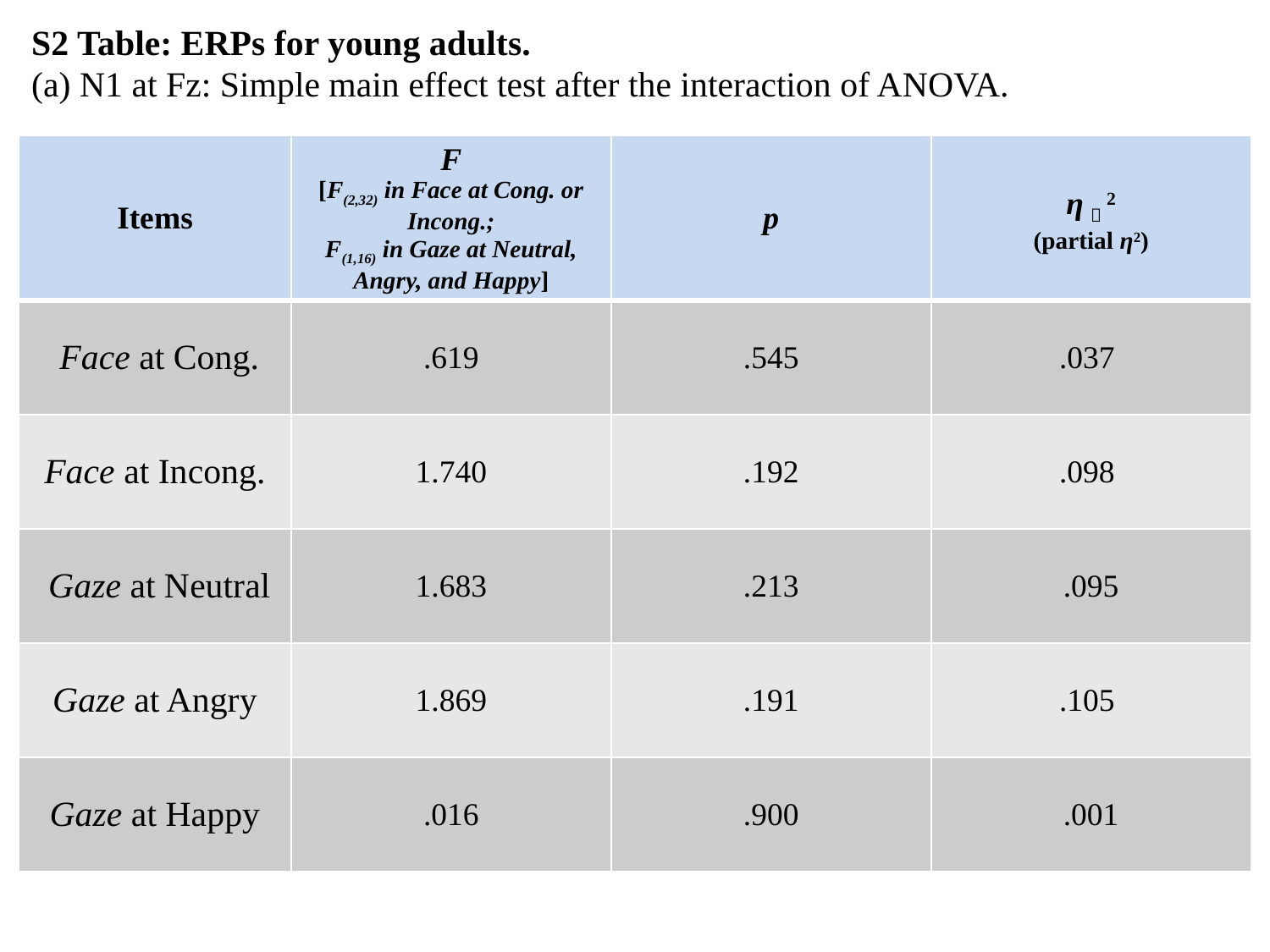

S2 Table: ERPs for young adults.
(a) N1 at Fz: Simple main effect test after the interaction of ANOVA.
| Items | F [F(2,32) in Face at Cong. or Incong.; F(1,16) in Gaze at Neutral, Angry, and Happy] | p | ηｐ2 (partial η2) |
| --- | --- | --- | --- |
| Face at Cong. | .619 | .545 | .037 |
| Face at Incong. | 1.740 | .192 | .098 |
| Gaze at Neutral | 1.683 | .213 | .095 |
| Gaze at Angry | 1.869 | .191 | .105 |
| Gaze at Happy | .016 | .900 | .001 |

## Slide 2
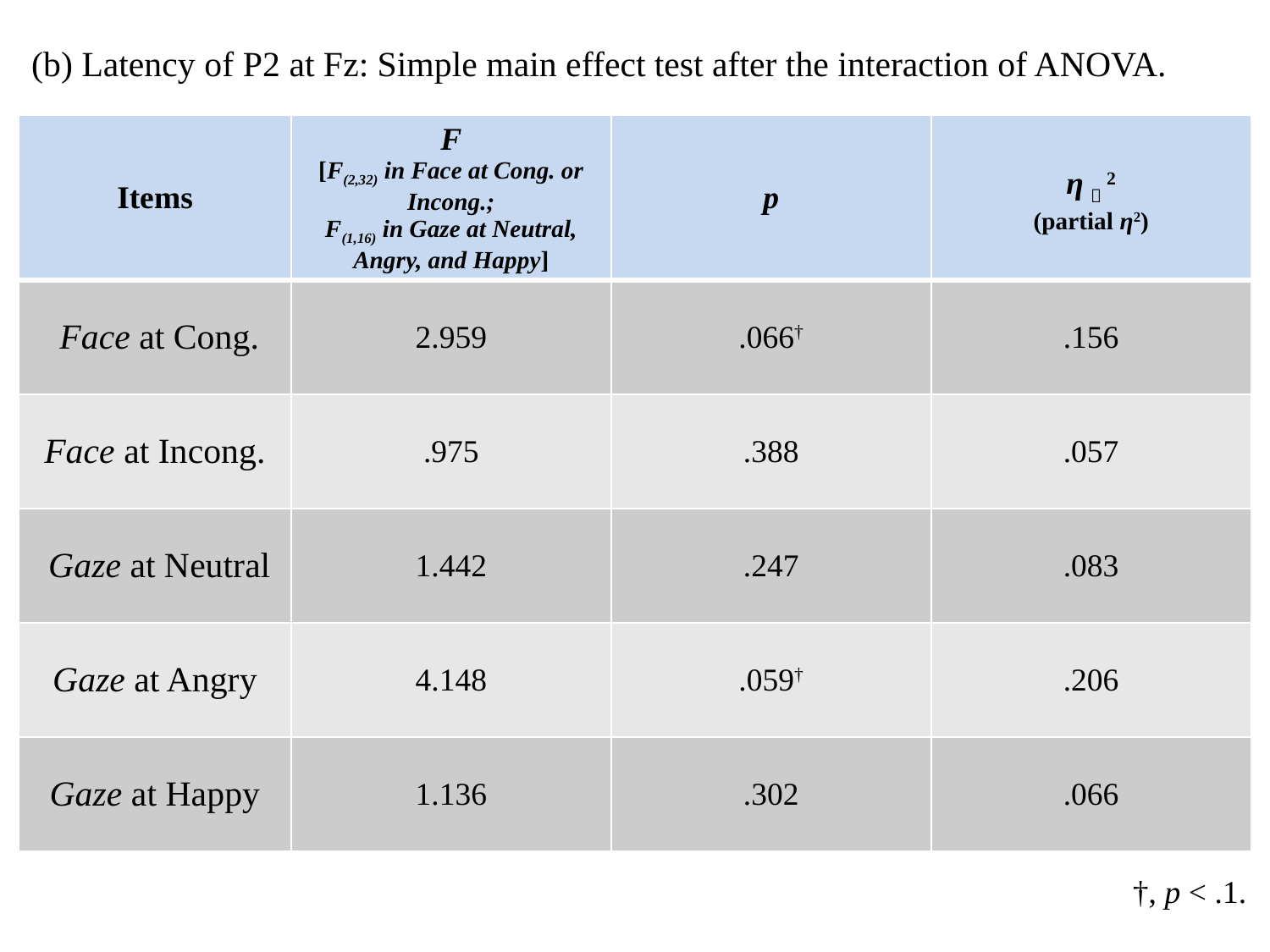

(b) Latency of P2 at Fz: Simple main effect test after the interaction of ANOVA.
| Items | F [F(2,32) in Face at Cong. or Incong.; F(1,16) in Gaze at Neutral, Angry, and Happy] | p | ηｐ2 (partial η2) |
| --- | --- | --- | --- |
| Face at Cong. | 2.959 | .066† | .156 |
| Face at Incong. | .975 | .388 | .057 |
| Gaze at Neutral | 1.442 | .247 | .083 |
| Gaze at Angry | 4.148 | .059† | .206 |
| Gaze at Happy | 1.136 | .302 | .066 |
†, p < .1.

## Slide 3
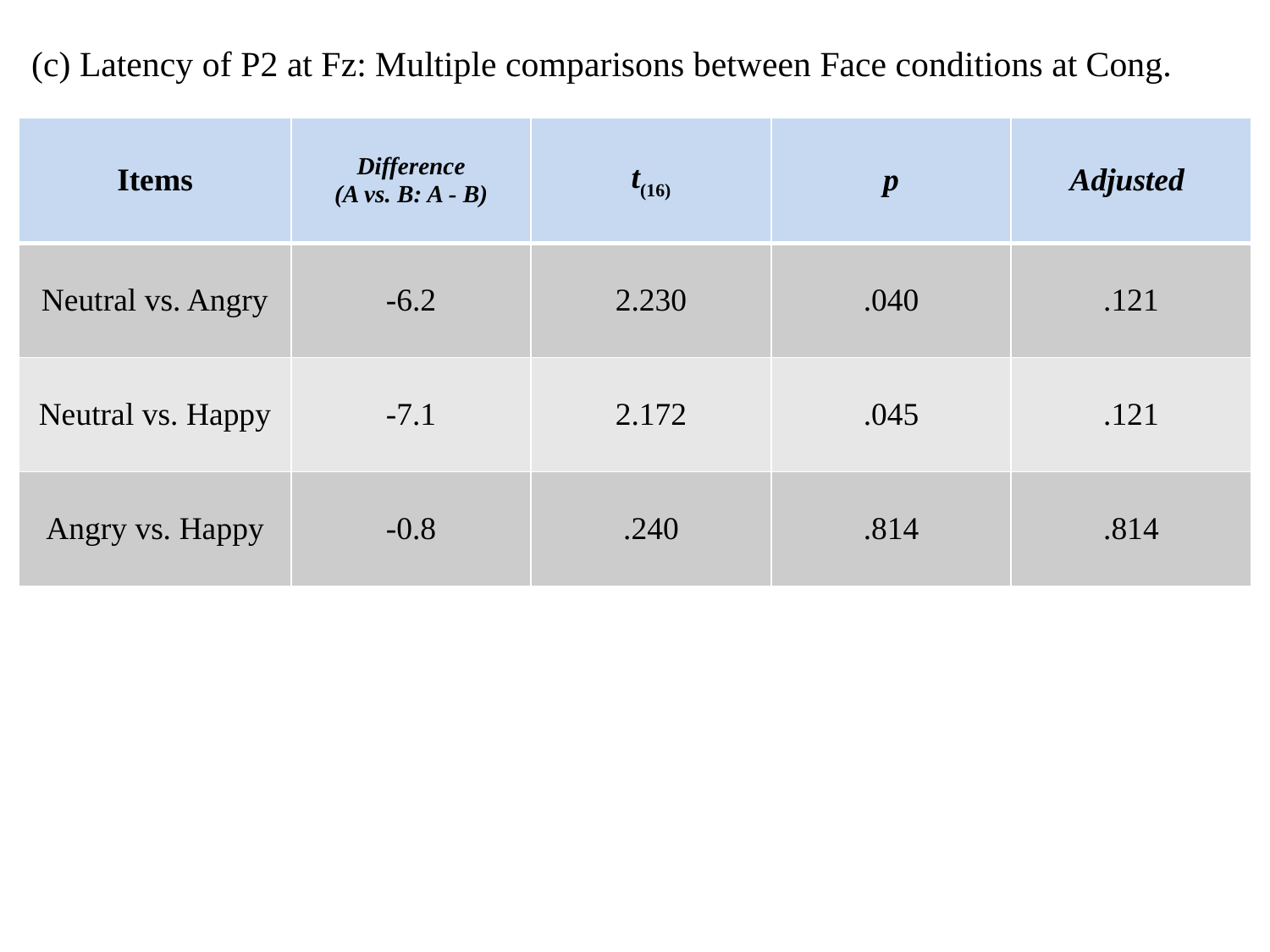

(c) Latency of P2 at Fz: Multiple comparisons between Face conditions at Cong.

## Slide 4
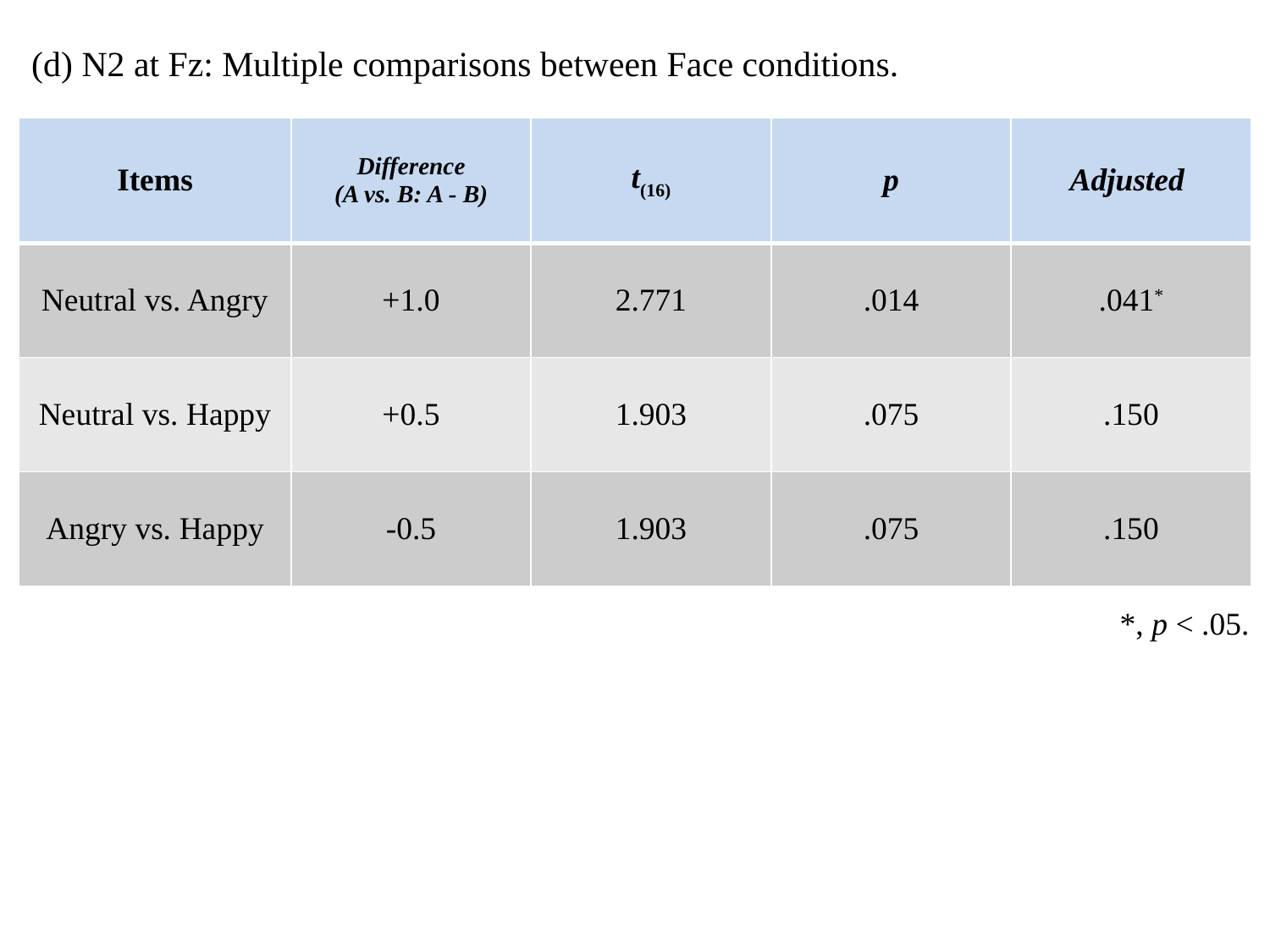

(d) N2 at Fz: Multiple comparisons between Face conditions.
*, p < .05.

## Slide 5
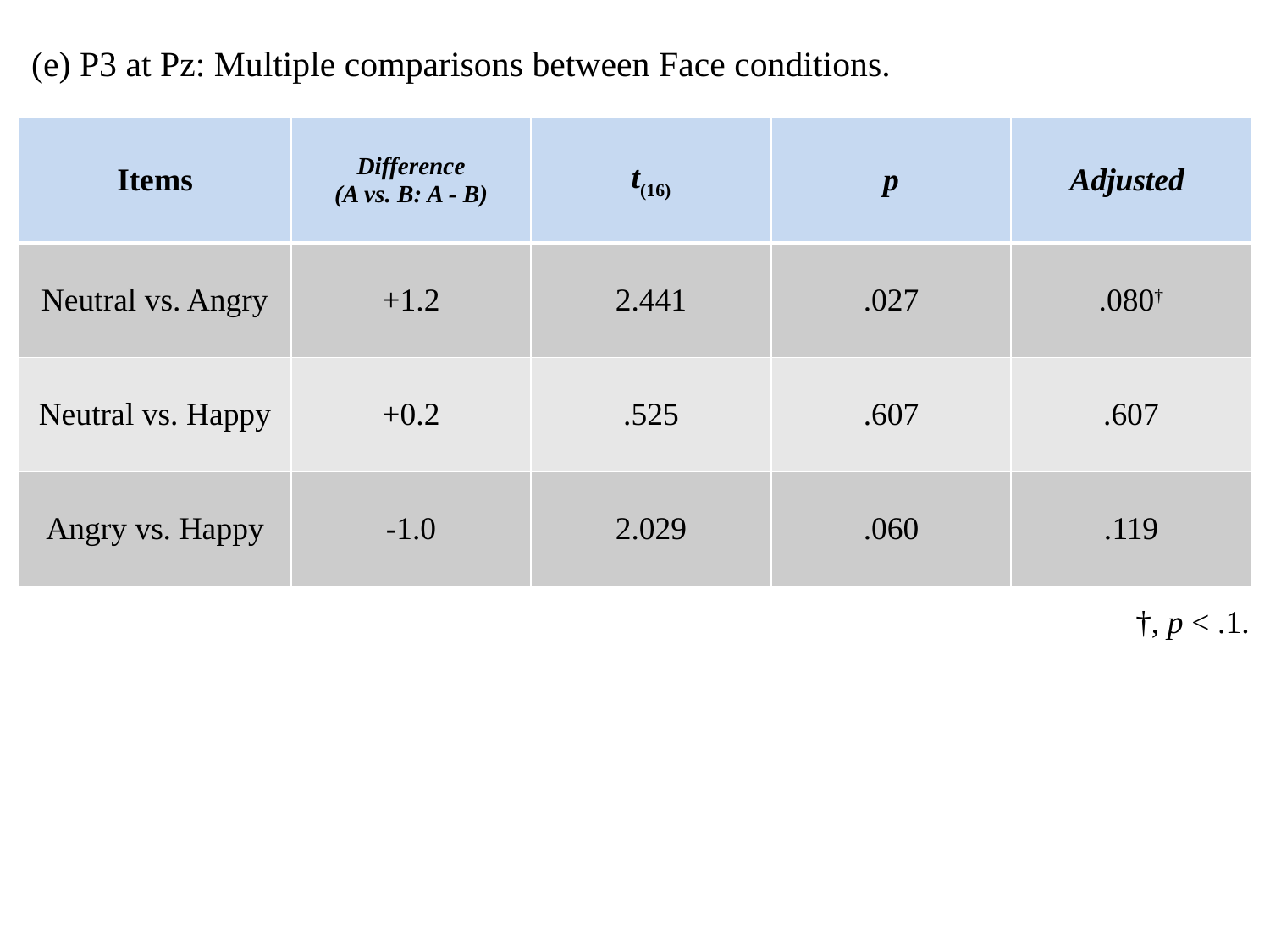

(e) P3 at Pz: Multiple comparisons between Face conditions.
†, p < .1.

## Slide 6
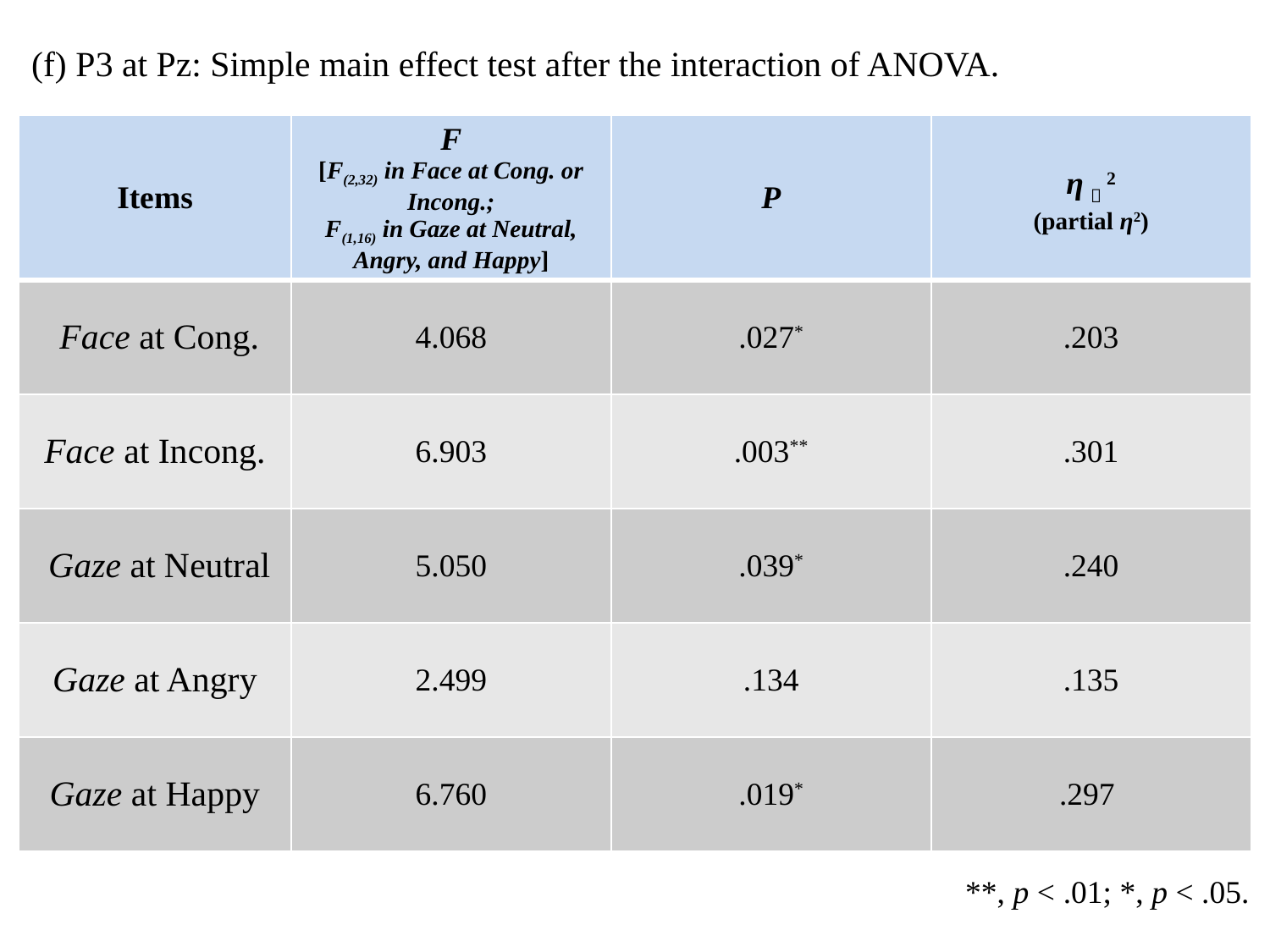

(f) P3 at Pz: Simple main effect test after the interaction of ANOVA.
| Items | F [F(2,32) in Face at Cong. or Incong.; F(1,16) in Gaze at Neutral, Angry, and Happy] | P | ηｐ2 (partial η2) |
| --- | --- | --- | --- |
| Face at Cong. | 4.068 | .027\* | .203 |
| Face at Incong. | 6.903 | .003\*\* | .301 |
| Gaze at Neutral | 5.050 | .039\* | .240 |
| Gaze at Angry | 2.499 | .134 | .135 |
| Gaze at Happy | 6.760 | .019\* | .297 |
**, p < .01; *, p < .05.

## Slide 7
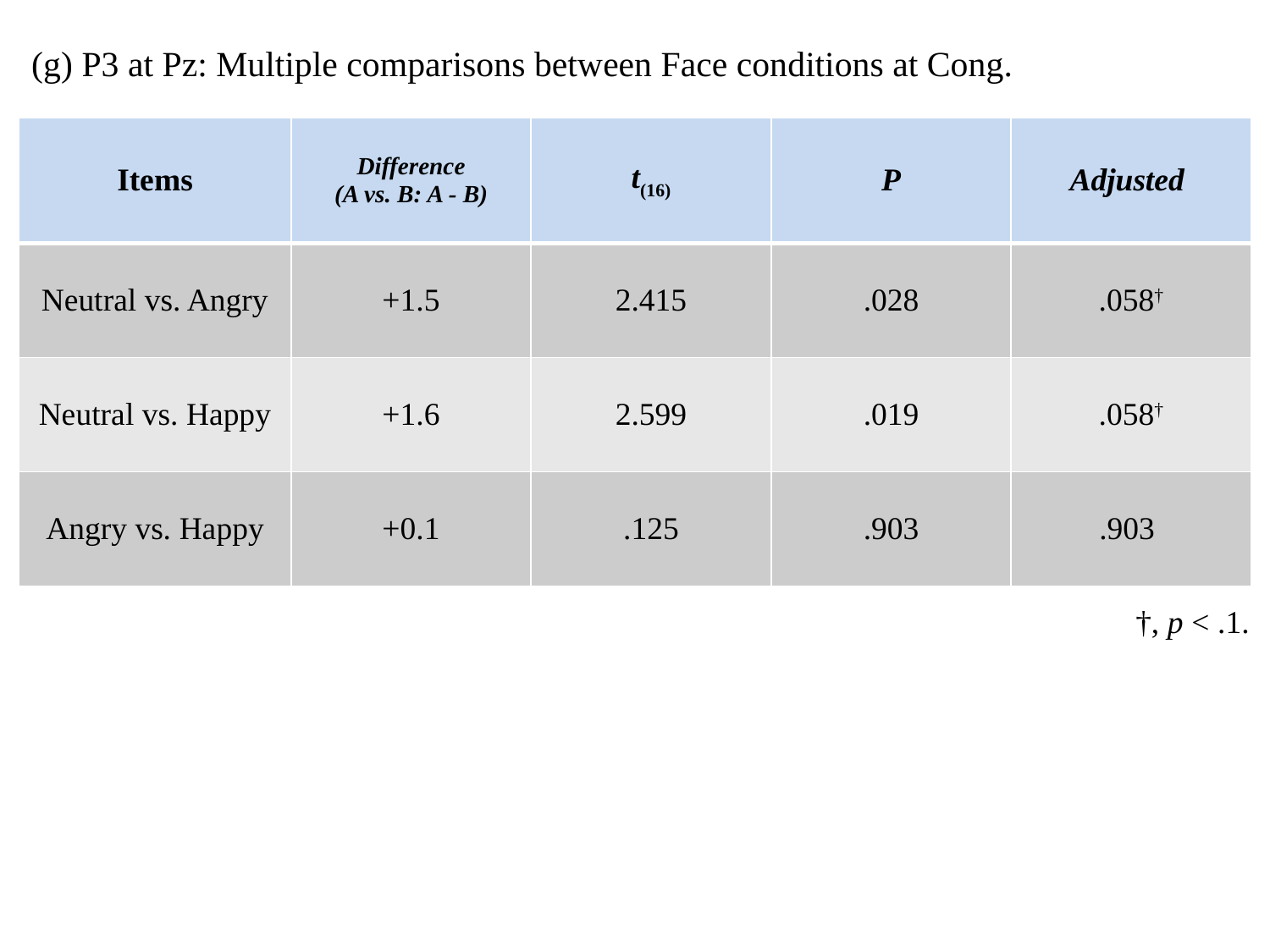

(g) P3 at Pz: Multiple comparisons between Face conditions at Cong.
†, p < .1.

## Slide 8
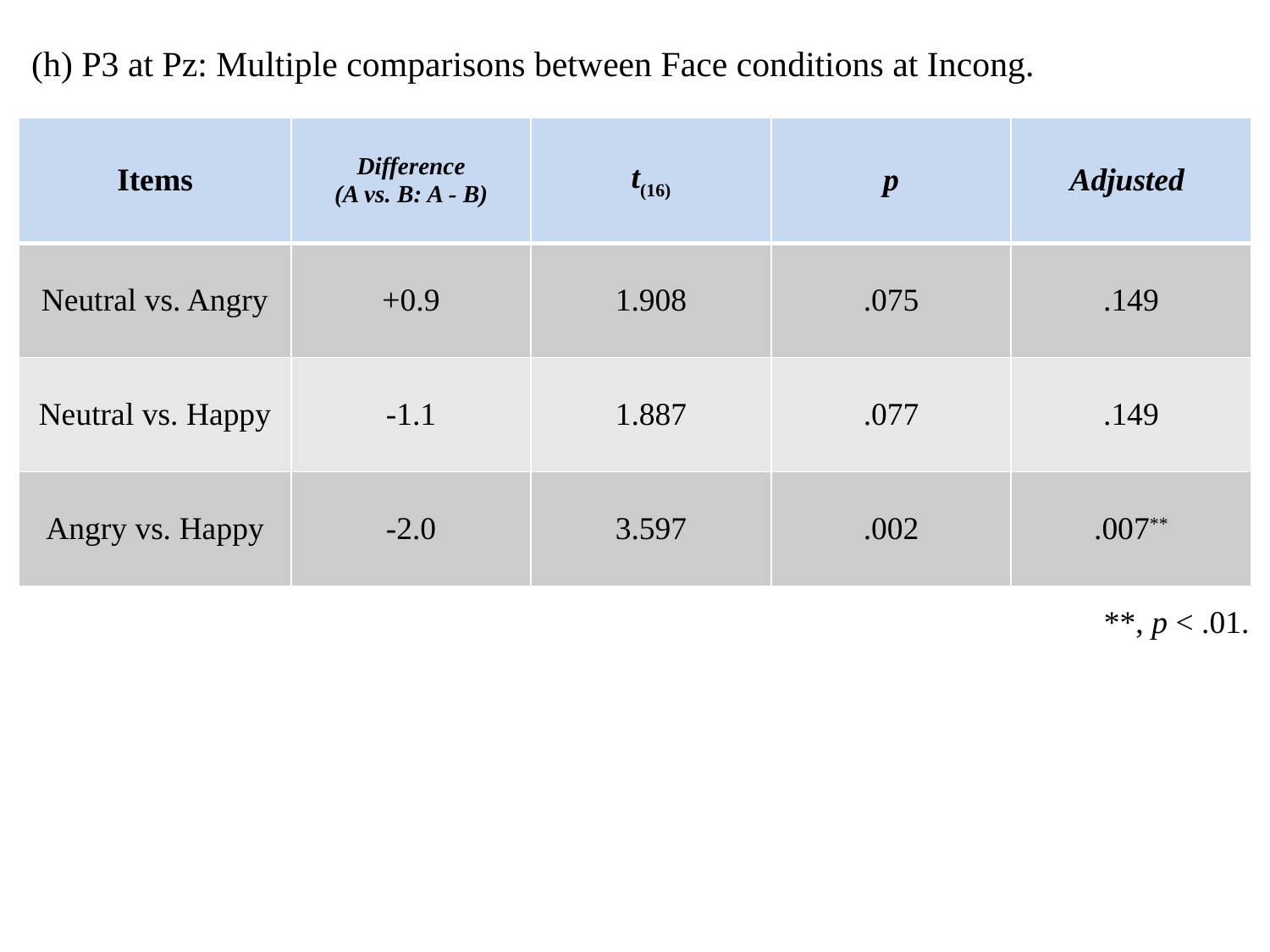

(h) P3 at Pz: Multiple comparisons between Face conditions at Incong.
**, p < .01.
