# Supplementary material for: Effects of facial expression and gaze interaction on brain dynamics during a working memory task in preschool children
Source: PLoS One. 2022 Apr 28;17(4):e0266713. doi: 10.1371/journal.pone.0266713 (PMC9049575; doi:10.1371/journal.pone.0266713)
Supplement: S4 Table — (a) The encoding period for Fmθ power: Simple main effect test after the interaction of ANOVA. (b) The encoding period for Pmα power: Multiple comparisons of Face conditions. (c) The latter retention period for Pmα power: Multiple comparisons between Face conditions. (PPTX) [file pone.0266713.s005.pptx]

## Slide 1
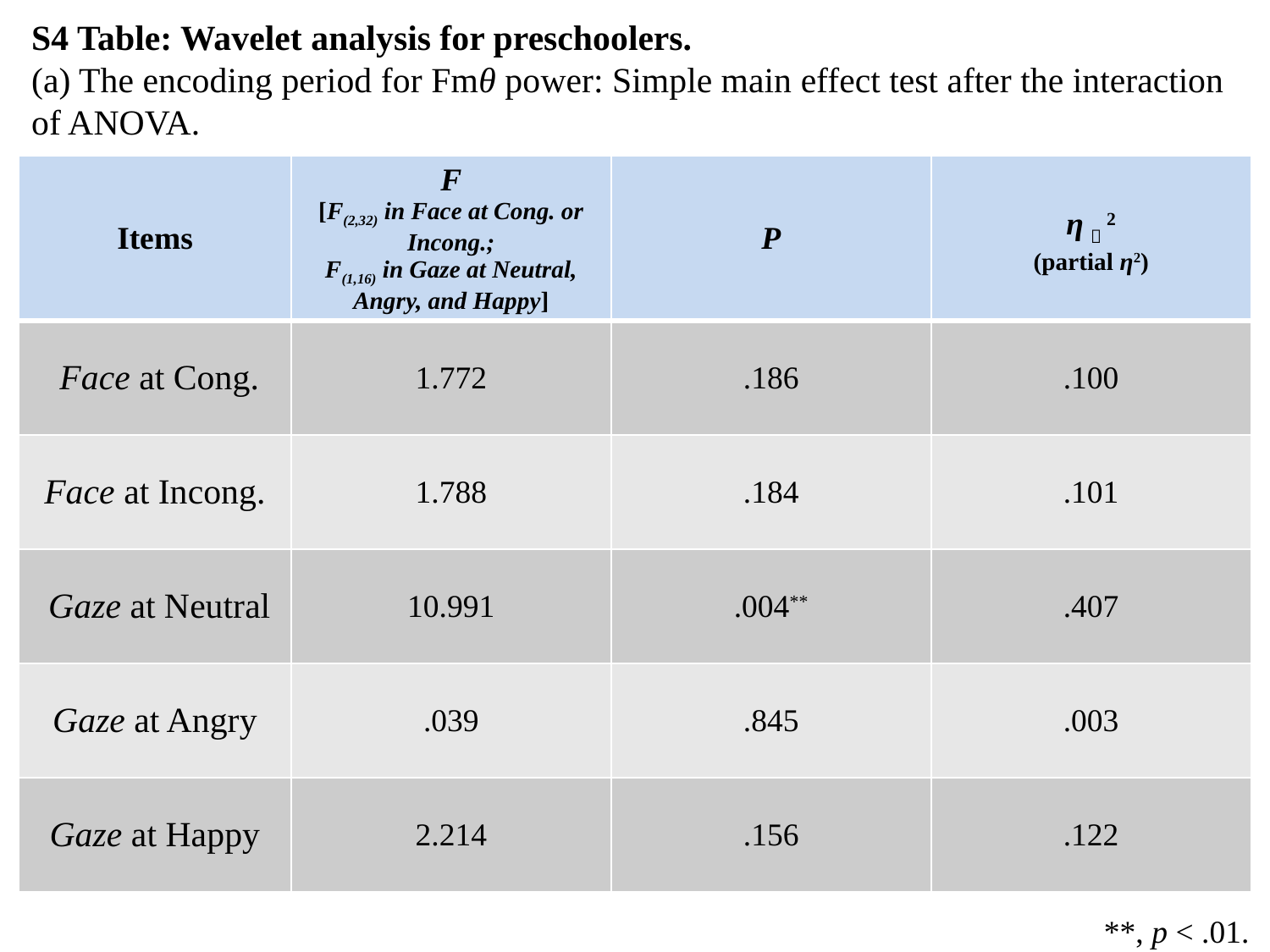

S4 Table: Wavelet analysis for preschoolers.
(a) The encoding period for Fmθ power: Simple main effect test after the interaction of ANOVA.
| Items | F [F(2,32) in Face at Cong. or Incong.; F(1,16) in Gaze at Neutral, Angry, and Happy] | P | ηｐ2 (partial η2) |
| --- | --- | --- | --- |
| Face at Cong. | 1.772 | .186 | .100 |
| Face at Incong. | 1.788 | .184 | .101 |
| Gaze at Neutral | 10.991 | .004\*\* | .407 |
| Gaze at Angry | .039 | .845 | .003 |
| Gaze at Happy | 2.214 | .156 | .122 |
**, p < .01.

## Slide 2
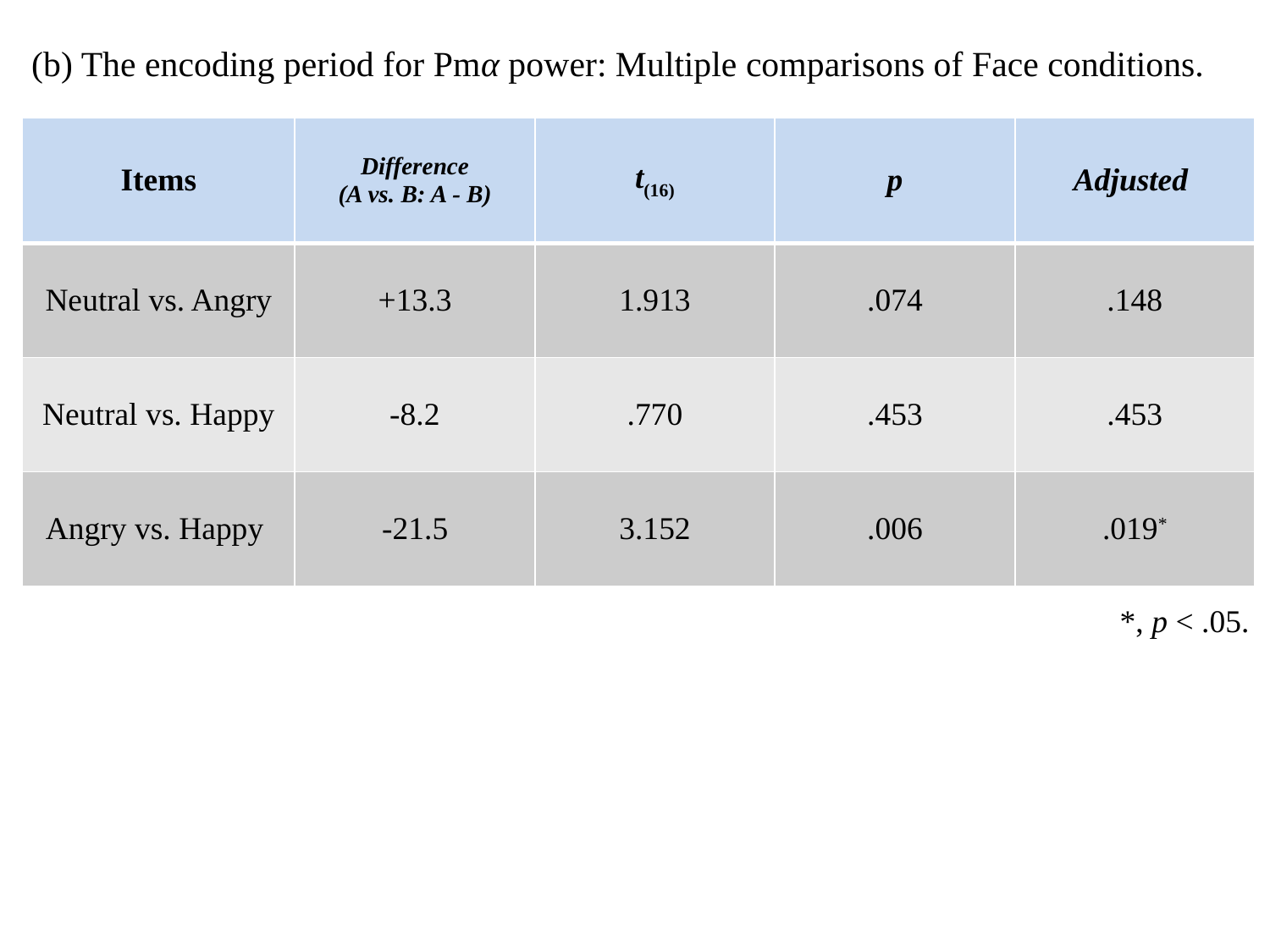

(b) The encoding period for Pmα power: Multiple comparisons of Face conditions.
*, p < .05.

## Slide 3
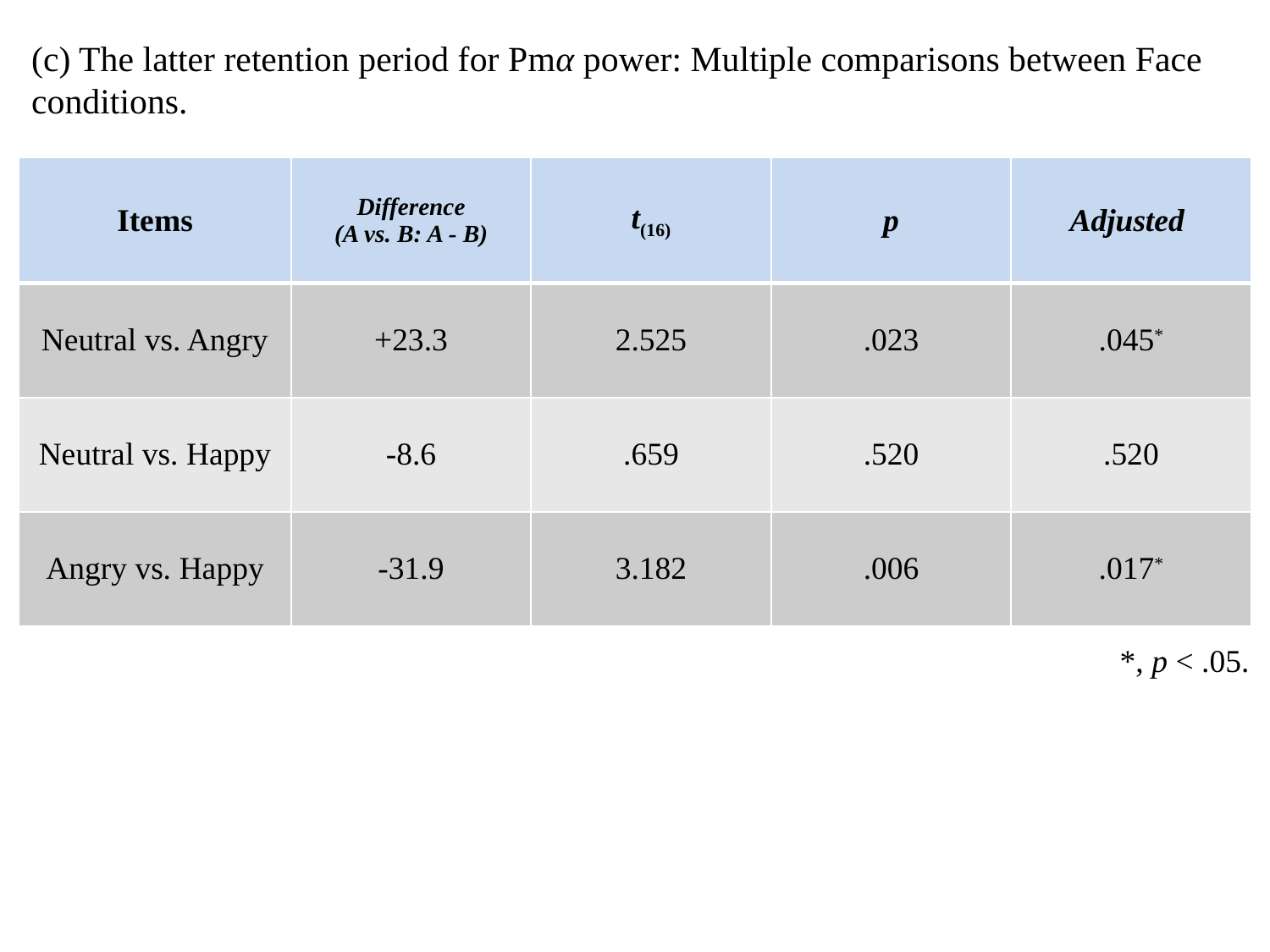

(c) The latter retention period for Pmα power: Multiple comparisons between Face conditions.
*, p < .05.
