# Supplementary material for: Effects of facial expression and gaze interaction on brain dynamics during a working memory task in preschool children
Source: PLoS One. 2022 Apr 28;17(4):e0266713. doi: 10.1371/journal.pone.0266713 (PMC9049575; doi:10.1371/journal.pone.0266713)
Supplement: S5 Table — (PPTX) [file pone.0266713.s006.pptx]

## Slide 1
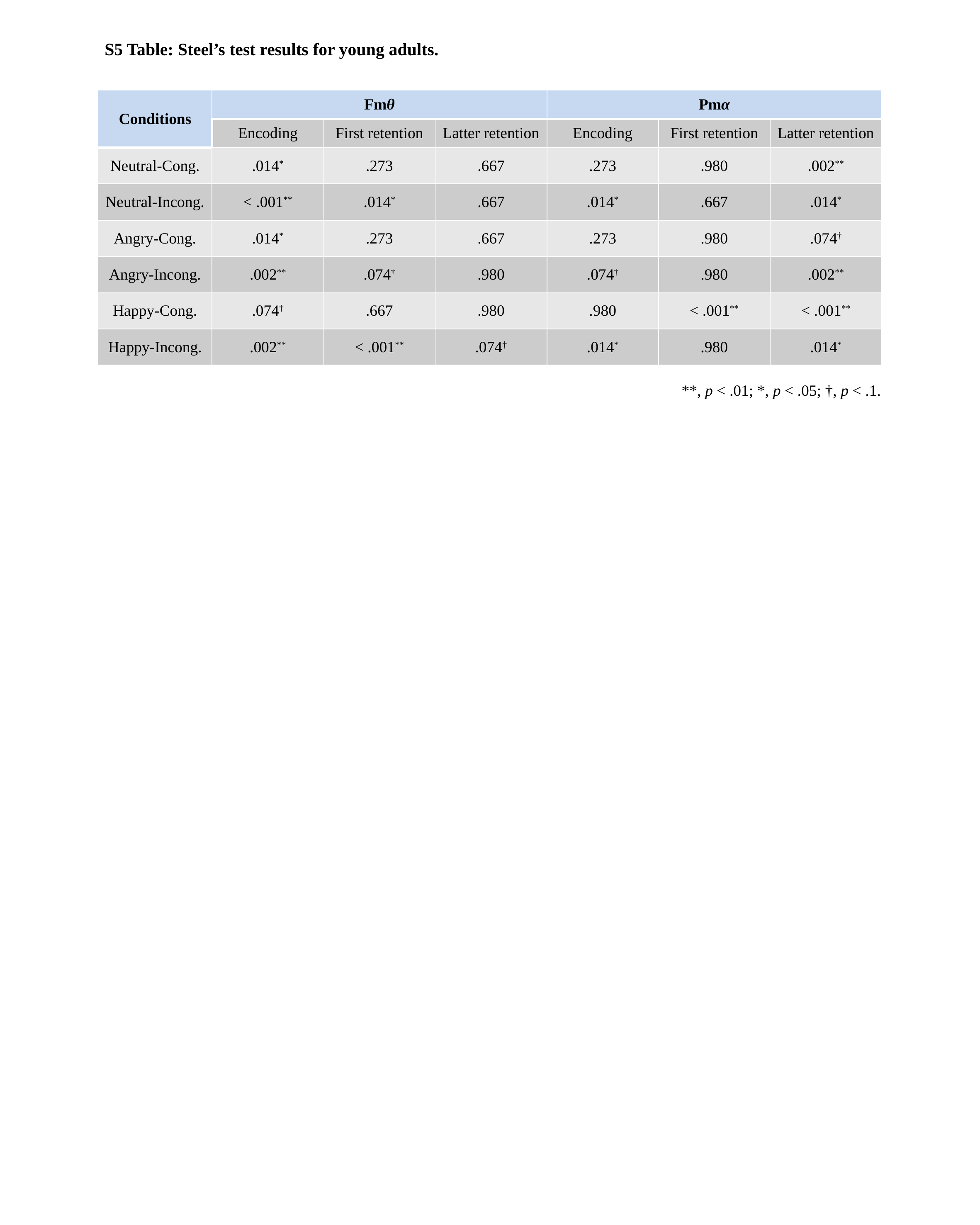

S5 Table: Steel’s test results for young adults.
| Conditions | Fmθ | | | Pmα | | |
| --- | --- | --- | --- | --- | --- | --- |
| | Encoding | First retention | Latter retention | Encoding | First retention | Latter retention |
| Neutral-Cong. | .014\* | .273 | .667 | .273 | .980 | .002\*\* |
| Neutral-Incong. | < .001\*\* | .014\* | .667 | .014\* | .667 | .014\* |
| Angry-Cong. | .014\* | .273 | .667 | .273 | .980 | .074† |
| Angry-Incong. | .002\*\* | .074† | .980 | .074† | .980 | .002\*\* |
| Happy-Cong. | .074† | .667 | .980 | .980 | < .001\*\* | < .001\*\* |
| Happy-Incong. | .002\*\* | < .001\*\* | .074† | .014\* | .980 | .014\* |
**, p < .01; *, p < .05; †, p < .1.
