# Supplementary material for: Effects of facial expression and gaze interaction on brain dynamics during a working memory task in preschool children
Source: PLoS One. 2022 Apr 28;17(4):e0266713. doi: 10.1371/journal.pone.0266713 (PMC9049575; doi:10.1371/journal.pone.0266713)
Supplement: S6 Table — (a) The first retention period for Fmθ power: Multiple comparisons between Face conditions. (b) The first retention period for Fmθ power: Simple main effect test after the interaction of ANOVA. (c) The first retention period for Fmθ power: Multiple comparisons between Face conditions at Incong. (d) The first retention period for Pmα power: Multiple comparisons between Face conditions. (PPTX) [file pone.0266713.s007.pptx]

## Slide 1
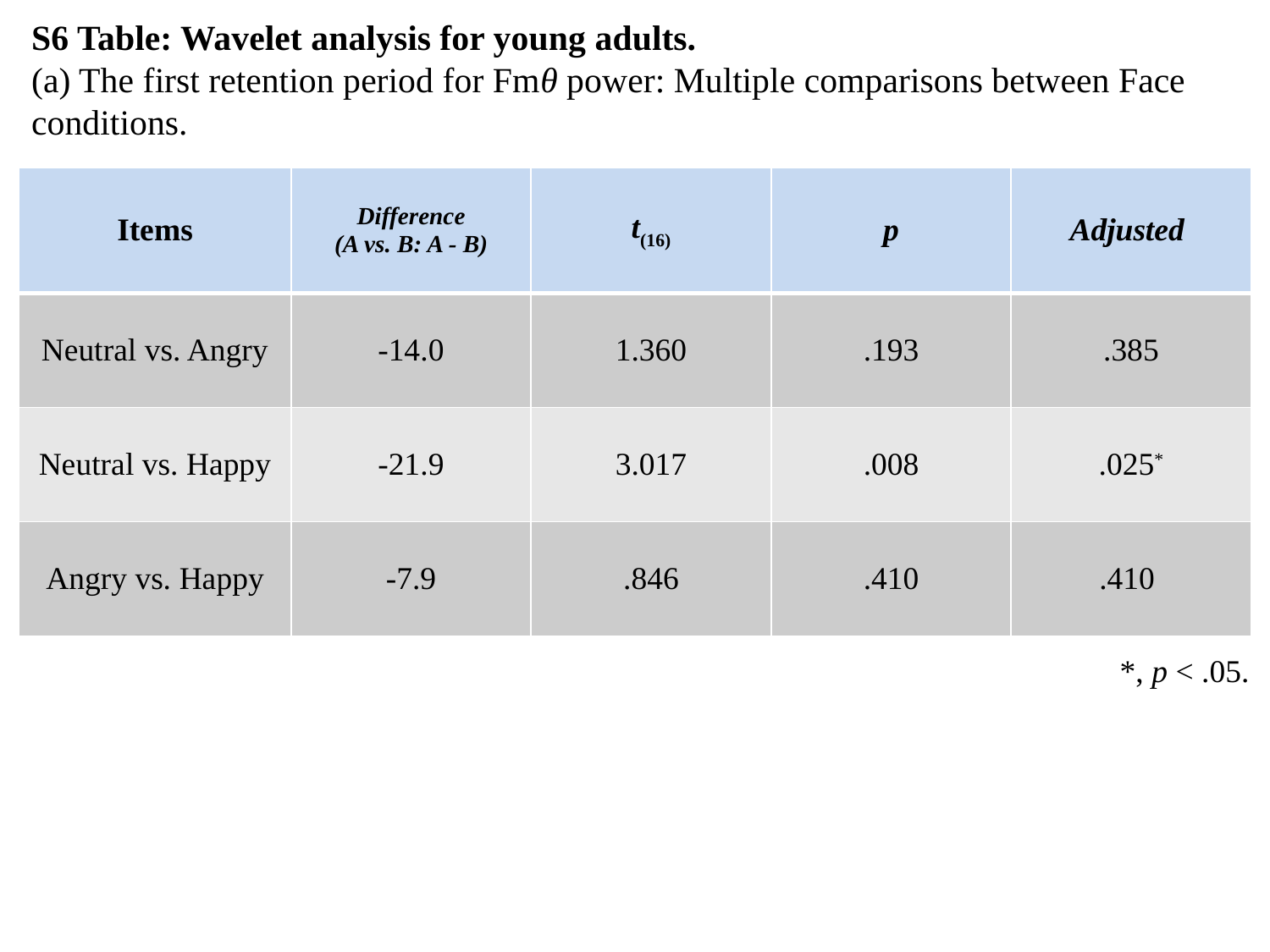

S6 Table: Wavelet analysis for young adults.
(a) The first retention period for Fmθ power: Multiple comparisons between Face conditions.
*, p < .05.

## Slide 2
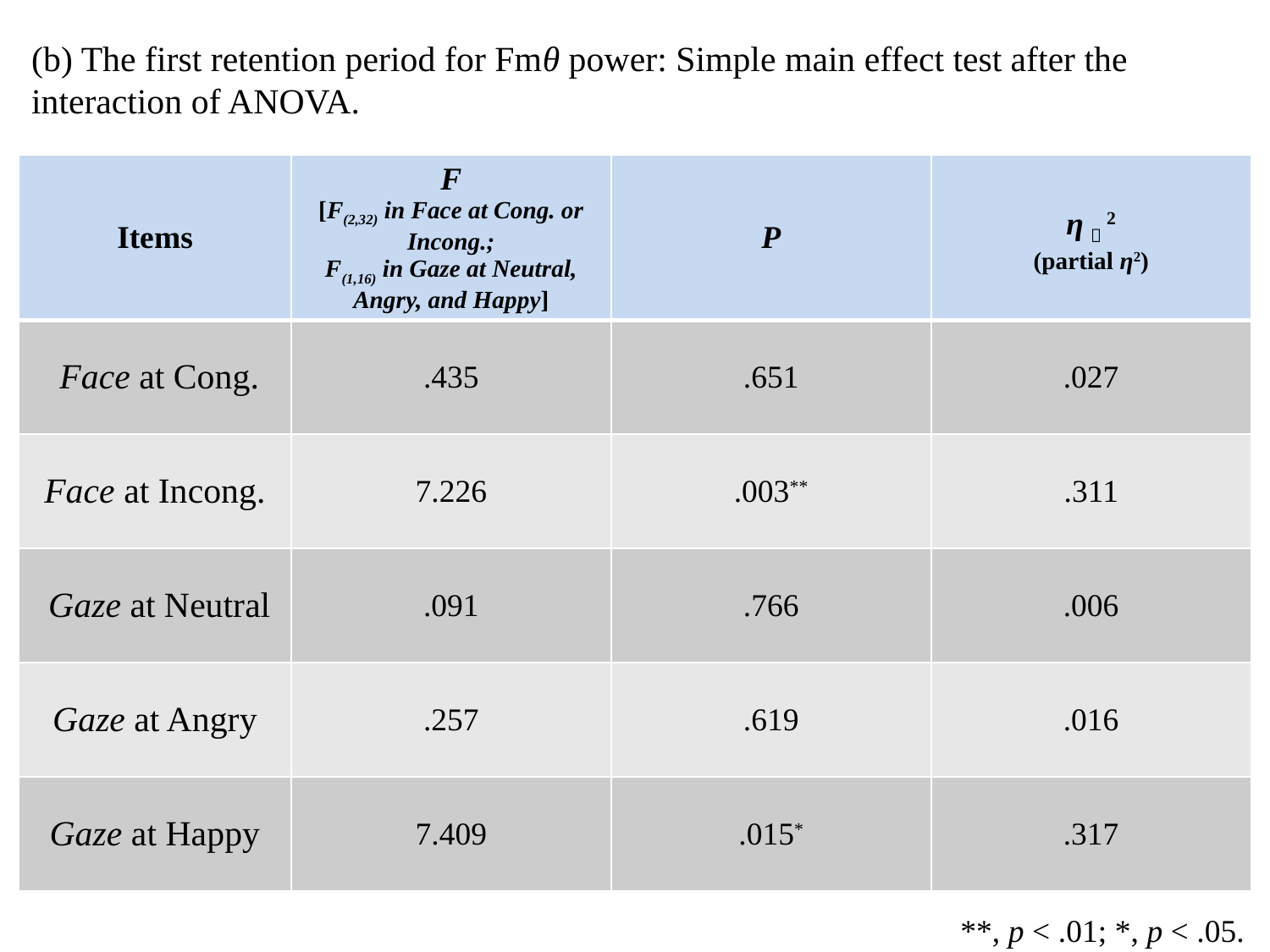

(b) The first retention period for Fmθ power: Simple main effect test after the interaction of ANOVA.
| Items | F [F(2,32) in Face at Cong. or Incong.; F(1,16) in Gaze at Neutral, Angry, and Happy] | P | ηｐ2 (partial η2) |
| --- | --- | --- | --- |
| Face at Cong. | .435 | .651 | .027 |
| Face at Incong. | 7.226 | .003\*\* | .311 |
| Gaze at Neutral | .091 | .766 | .006 |
| Gaze at Angry | .257 | .619 | .016 |
| Gaze at Happy | 7.409 | .015\* | .317 |
**, p < .01; *, p < .05.

## Slide 3
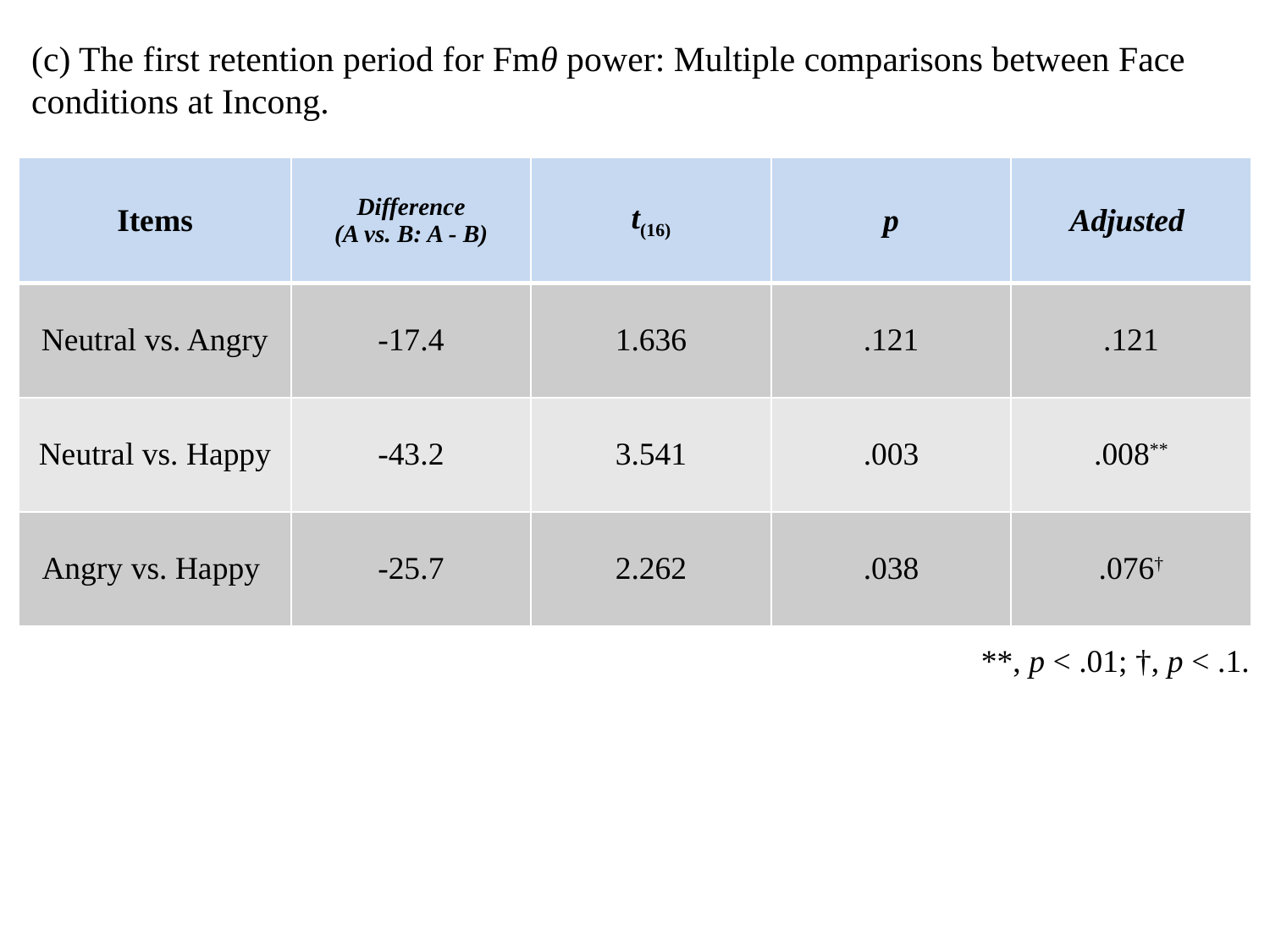

(c) The first retention period for Fmθ power: Multiple comparisons between Face conditions at Incong.
**, p < .01; †, p < .1.

## Slide 4
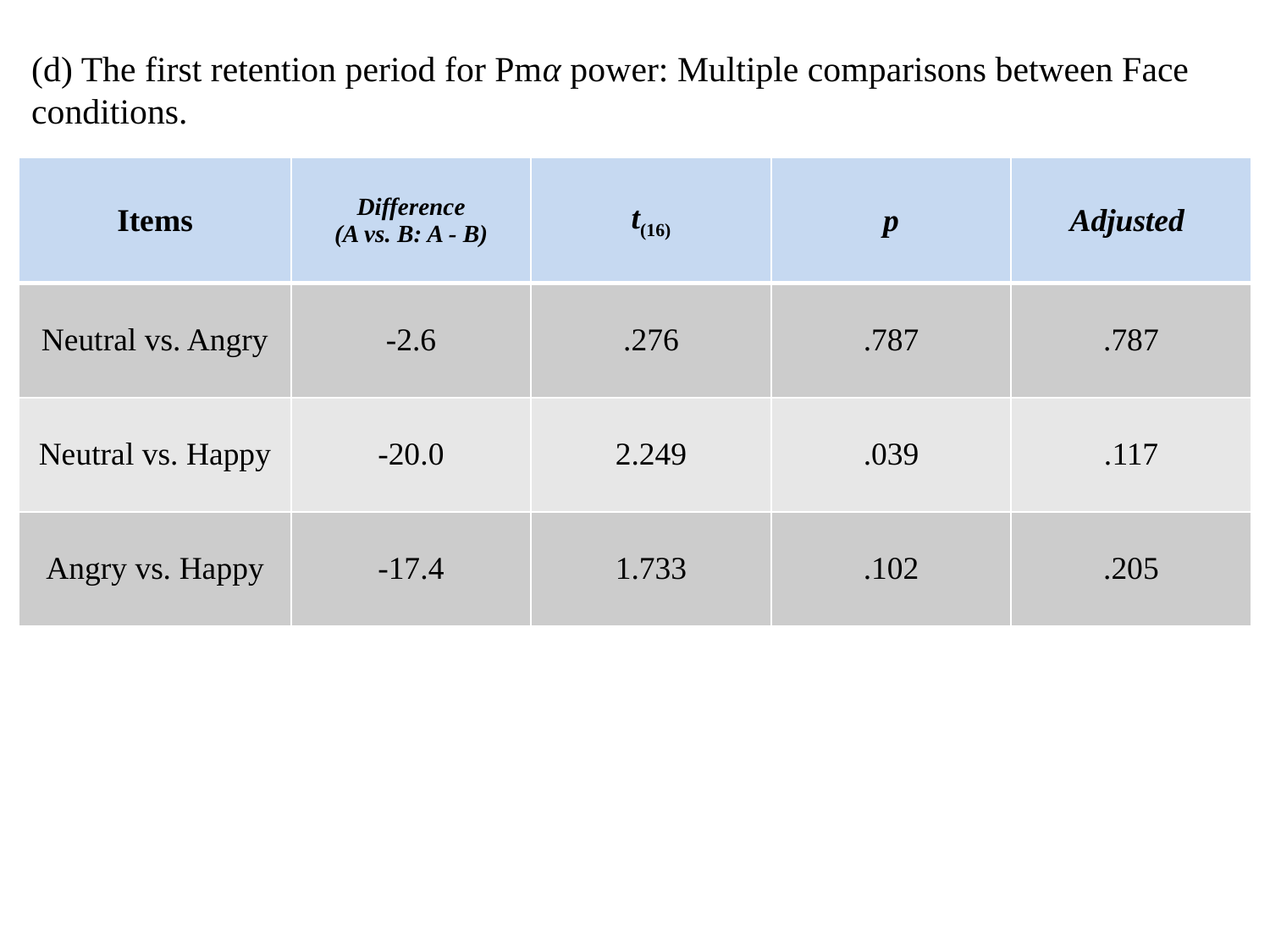

(d) The first retention period for Pmα power: Multiple comparisons between Face conditions.
