# Supplementary material for: Effects of facial expression and gaze interaction on brain dynamics during a working memory task in preschool children
Source: PLoS One. 2022 Apr 28;17(4):e0266713. doi: 10.1371/journal.pone.0266713 (PMC9049575; doi:10.1371/journal.pone.0266713)
Supplement: S7 Table — (a) Simple main effect test after the interaction of ANOVA. (b) Multiple comparisons between Face conditions at Incong. (PPTX) [file pone.0266713.s008.pptx]

## Slide 1
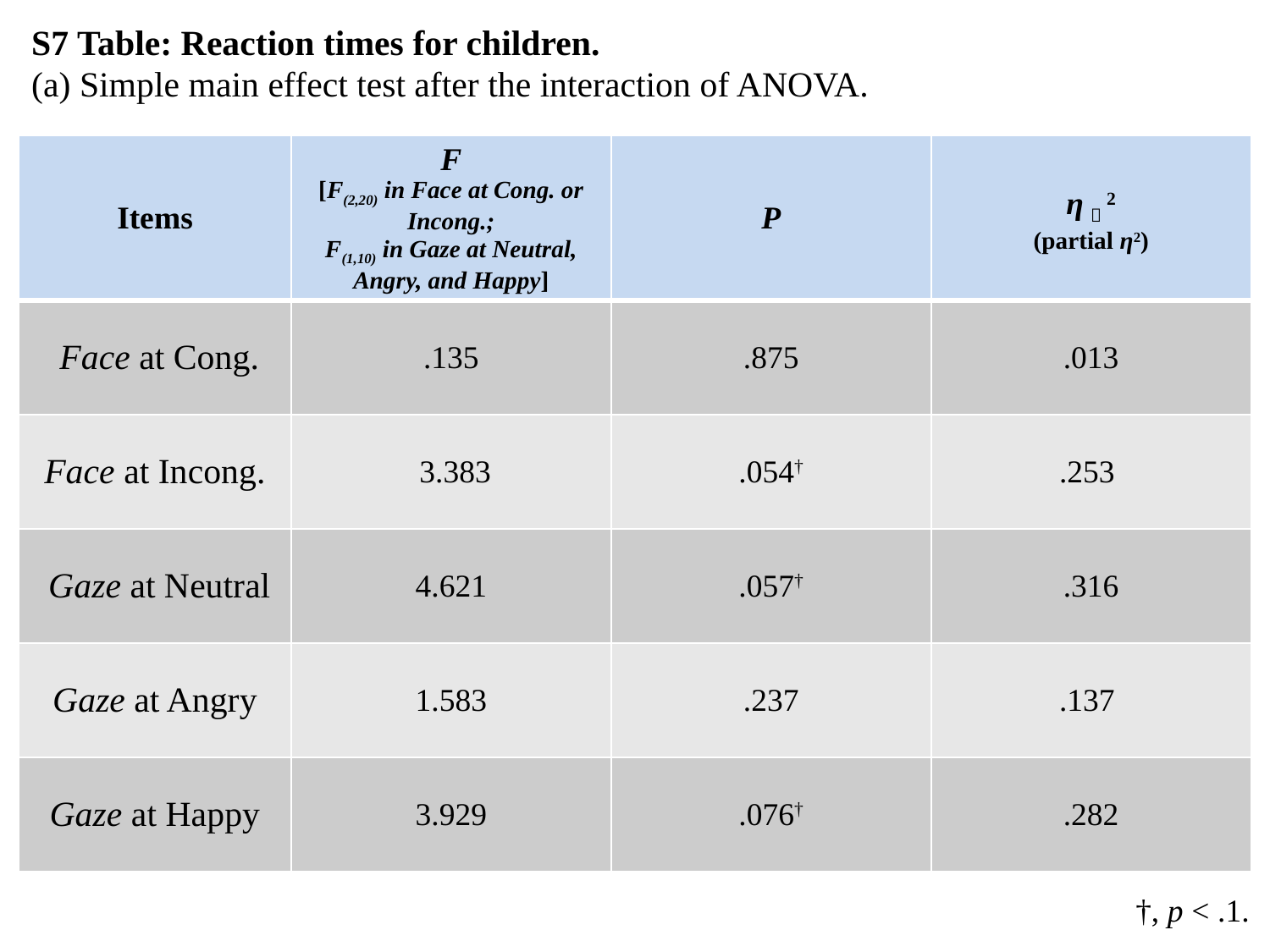

S7 Table: Reaction times for children.
(a) Simple main effect test after the interaction of ANOVA.
| Items | F [F(2,20) in Face at Cong. or Incong.; F(1,10) in Gaze at Neutral, Angry, and Happy] | P | ηｐ2 (partial η2) |
| --- | --- | --- | --- |
| Face at Cong. | .135 | .875 | .013 |
| Face at Incong. | 3.383 | .054† | .253 |
| Gaze at Neutral | 4.621 | .057† | .316 |
| Gaze at Angry | 1.583 | .237 | .137 |
| Gaze at Happy | 3.929 | .076† | .282 |
†, p < .1.

## Slide 2
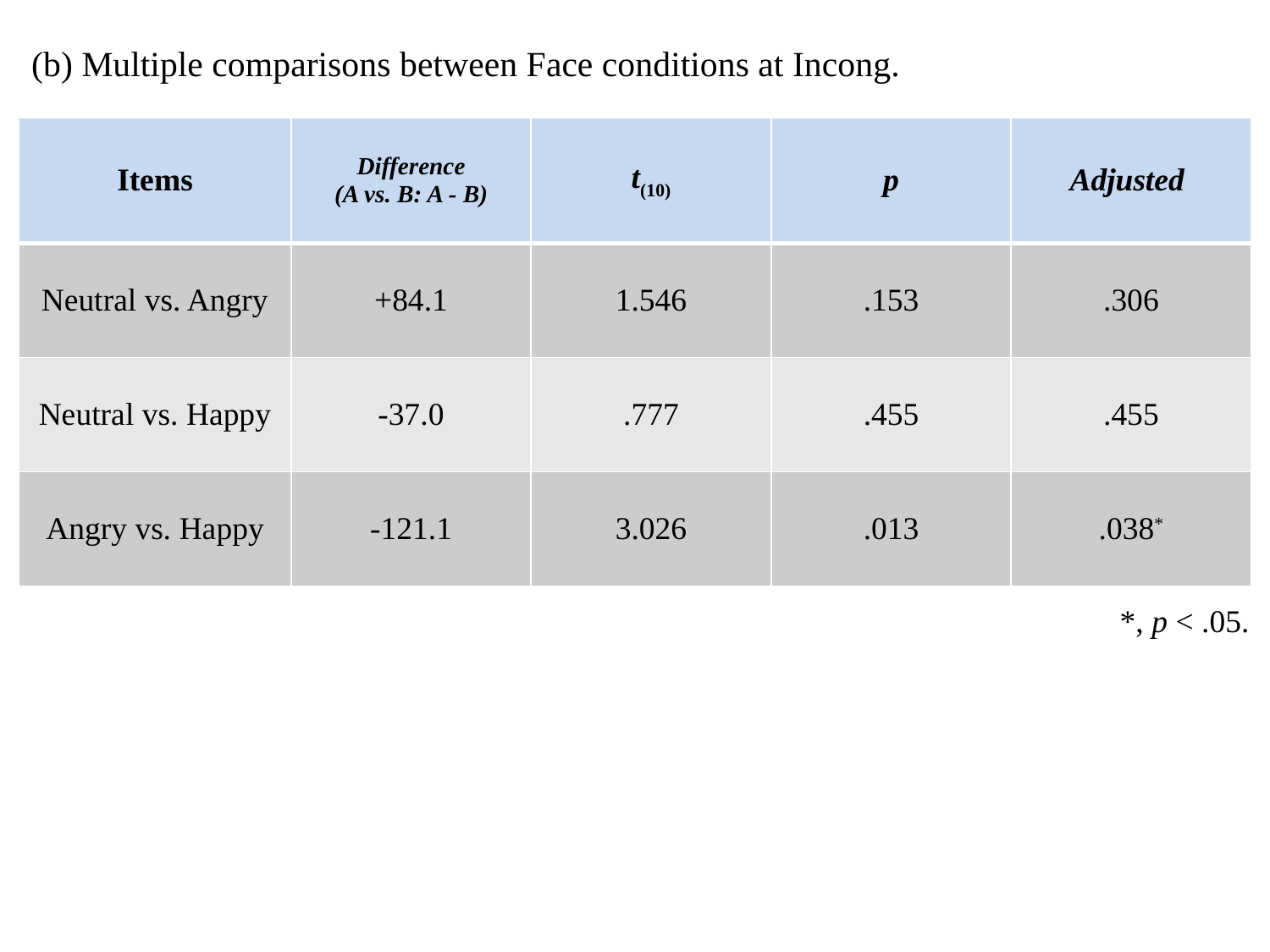

(b) Multiple comparisons between Face conditions at Incong.
*, p < .05.
