# Supplementary material for: Effects of facial expression and gaze interaction on brain dynamics during a working memory task in preschool children
Source: PLoS One. 2022 Apr 28;17(4):e0266713. doi: 10.1371/journal.pone.0266713 (PMC9049575; doi:10.1371/journal.pone.0266713)
Supplement: S8 Table — (a) Simple main effect test after the interaction of ANOVA. (b) Multiple comparisons between Face conditions at Cong. (PPTX) [file pone.0266713.s009.pptx]

## Slide 1
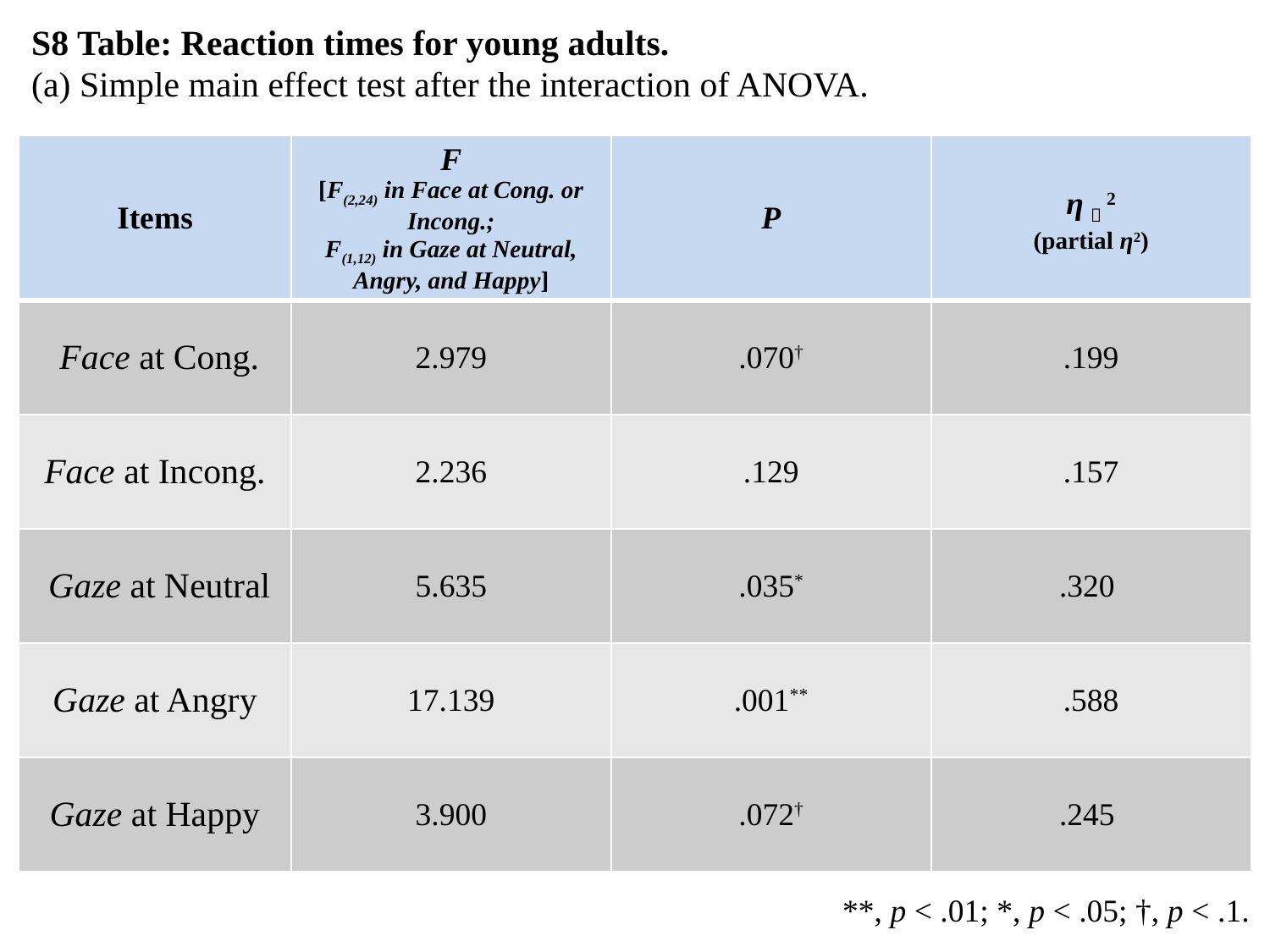

S8 Table: Reaction times for young adults.
(a) Simple main effect test after the interaction of ANOVA.
| Items | F [F(2,24) in Face at Cong. or Incong.; F(1,12) in Gaze at Neutral, Angry, and Happy] | P | ηｐ2 (partial η2) |
| --- | --- | --- | --- |
| Face at Cong. | 2.979 | .070† | .199 |
| Face at Incong. | 2.236 | .129 | .157 |
| Gaze at Neutral | 5.635 | .035\* | .320 |
| Gaze at Angry | 17.139 | .001\*\* | .588 |
| Gaze at Happy | 3.900 | .072† | .245 |
**, p < .01; *, p < .05; †, p < .1.

## Slide 2
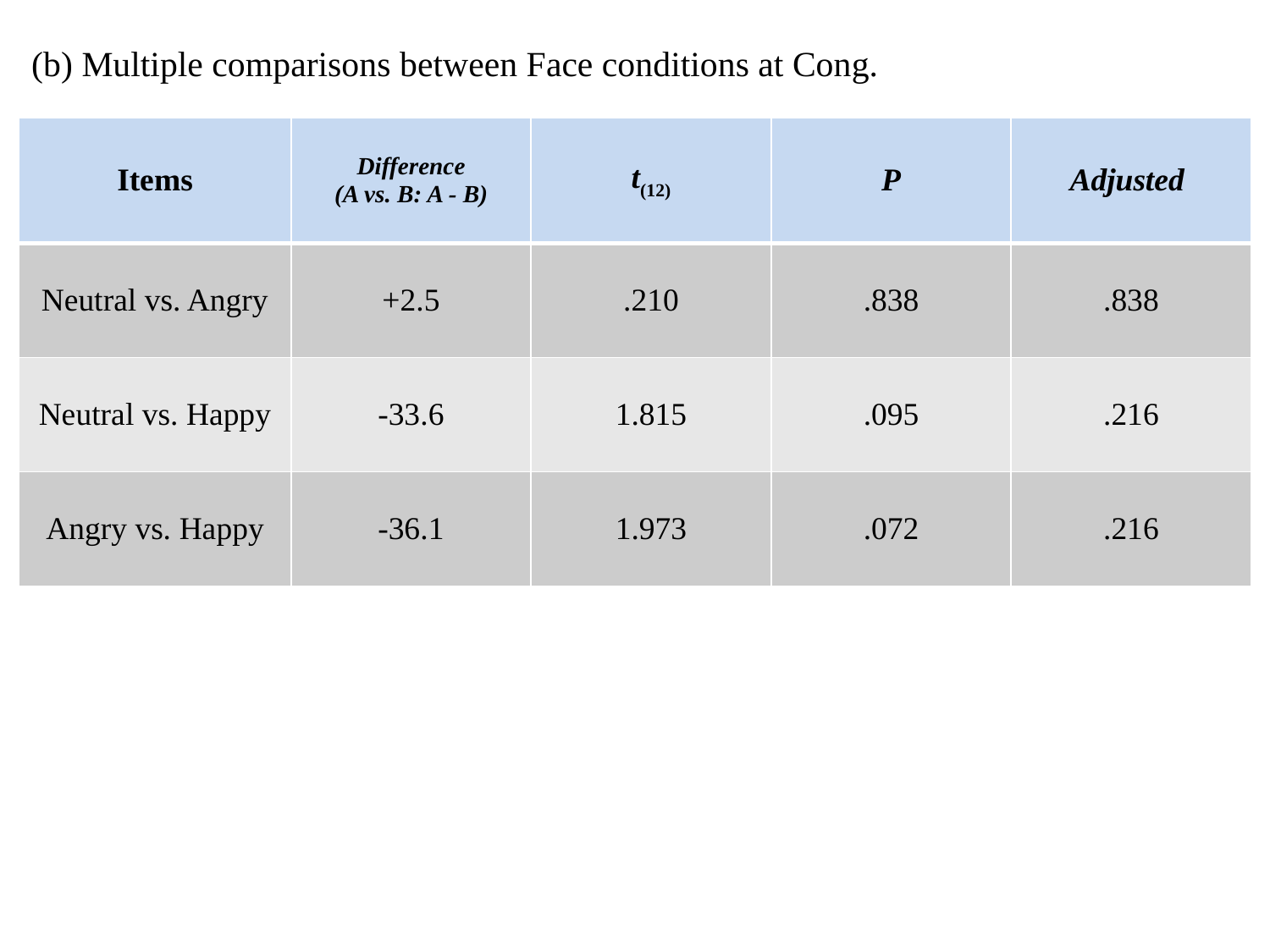

(b) Multiple comparisons between Face conditions at Cong.
