# Supplementary material for: Effects of facial expression and gaze interaction on brain dynamics during a working memory task in preschool children
Source: PLoS One. 2022 Apr 28;17(4):e0266713. doi: 10.1371/journal.pone.0266713 (PMC9049575; doi:10.1371/journal.pone.0266713)
Supplement: S9 Table — (PPTX) [file pone.0266713.s010.pptx]

## Slide 1
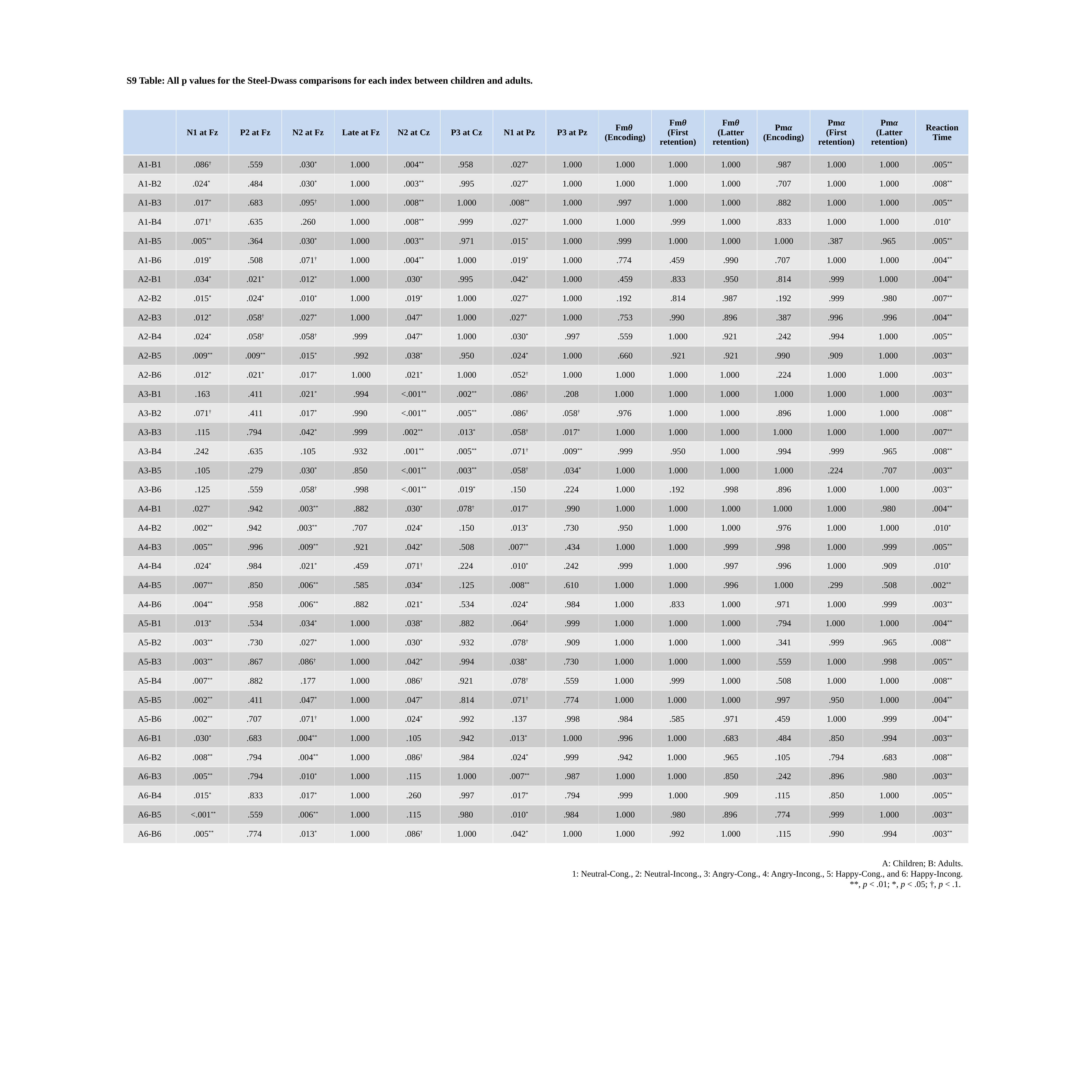

S9 Table: All p values for the Steel-Dwass comparisons for each index between children and adults.
| | N1 at Fz | P2 at Fz | N2 at Fz | Late at Fz | N2 at Cz | P3 at Cz | N1 at Pz | P3 at Pz | Fmθ (Encoding) | Fmθ (First retention) | Fmθ (Latter retention) | Pmα (Encoding) | Pmα (First retention) | Pmα (Latter retention) | Reaction Time |
| --- | --- | --- | --- | --- | --- | --- | --- | --- | --- | --- | --- | --- | --- | --- | --- |
| A1-B1 | .086† | .559 | .030\* | 1.000 | .004\*\* | .958 | .027\* | 1.000 | 1.000 | 1.000 | 1.000 | .987 | 1.000 | 1.000 | .005\*\* |
| A1-B2 | .024\* | .484 | .030\* | 1.000 | .003\*\* | .995 | .027\* | 1.000 | 1.000 | 1.000 | 1.000 | .707 | 1.000 | 1.000 | .008\*\* |
| A1-B3 | .017\* | .683 | .095† | 1.000 | .008\*\* | 1.000 | .008\*\* | 1.000 | .997 | 1.000 | 1.000 | .882 | 1.000 | 1.000 | .005\*\* |
| A1-B4 | .071† | .635 | .260 | 1.000 | .008\*\* | .999 | .027\* | 1.000 | 1.000 | .999 | 1.000 | .833 | 1.000 | 1.000 | .010\* |
| A1-B5 | .005\*\* | .364 | .030\* | 1.000 | .003\*\* | .971 | .015\* | 1.000 | .999 | 1.000 | 1.000 | 1.000 | .387 | .965 | .005\*\* |
| A1-B6 | .019\* | .508 | .071† | 1.000 | .004\*\* | 1.000 | .019\* | 1.000 | .774 | .459 | .990 | .707 | 1.000 | 1.000 | .004\*\* |
| A2-B1 | .034\* | .021\* | .012\* | 1.000 | .030\* | .995 | .042\* | 1.000 | .459 | .833 | .950 | .814 | .999 | 1.000 | .004\*\* |
| A2-B2 | .015\* | .024\* | .010\* | 1.000 | .019\* | 1.000 | .027\* | 1.000 | .192 | .814 | .987 | .192 | .999 | .980 | .007\*\* |
| A2-B3 | .012\* | .058† | .027\* | 1.000 | .047\* | 1.000 | .027\* | 1.000 | .753 | .990 | .896 | .387 | .996 | .996 | .004\*\* |
| A2-B4 | .024\* | .058† | .058† | .999 | .047\* | 1.000 | .030\* | .997 | .559 | 1.000 | .921 | .242 | .994 | 1.000 | .005\*\* |
| A2-B5 | .009\*\* | .009\*\* | .015\* | .992 | .038\* | .950 | .024\* | 1.000 | .660 | .921 | .921 | .990 | .909 | 1.000 | .003\*\* |
| A2-B6 | .012\* | .021\* | .017\* | 1.000 | .021\* | 1.000 | .052† | 1.000 | 1.000 | 1.000 | 1.000 | .224 | 1.000 | 1.000 | .003\*\* |
| A3-B1 | .163 | .411 | .021\* | .994 | <.001\*\* | .002\*\* | .086† | .208 | 1.000 | 1.000 | 1.000 | 1.000 | 1.000 | 1.000 | .003\*\* |
| A3-B2 | .071† | .411 | .017\* | .990 | <.001\*\* | .005\*\* | .086† | .058† | .976 | 1.000 | 1.000 | .896 | 1.000 | 1.000 | .008\*\* |
| A3-B3 | .115 | .794 | .042\* | .999 | .002\*\* | .013\* | .058† | .017\* | 1.000 | 1.000 | 1.000 | 1.000 | 1.000 | 1.000 | .007\*\* |
| A3-B4 | .242 | .635 | .105 | .932 | .001\*\* | .005\*\* | .071† | .009\*\* | .999 | .950 | 1.000 | .994 | .999 | .965 | .008\*\* |
| A3-B5 | .105 | .279 | .030\* | .850 | <.001\*\* | .003\*\* | .058† | .034\* | 1.000 | 1.000 | 1.000 | 1.000 | .224 | .707 | .003\*\* |
| A3-B6 | .125 | .559 | .058† | .998 | <.001\*\* | .019\* | .150 | .224 | 1.000 | .192 | .998 | .896 | 1.000 | 1.000 | .003\*\* |
| A4-B1 | .027\* | .942 | .003\*\* | .882 | .030\* | .078† | .017\* | .990 | 1.000 | 1.000 | 1.000 | 1.000 | 1.000 | .980 | .004\*\* |
| A4-B2 | .002\*\* | .942 | .003\*\* | .707 | .024\* | .150 | .013\* | .730 | .950 | 1.000 | 1.000 | .976 | 1.000 | 1.000 | .010\* |
| A4-B3 | .005\*\* | .996 | .009\*\* | .921 | .042\* | .508 | .007\*\* | .434 | 1.000 | 1.000 | .999 | .998 | 1.000 | .999 | .005\*\* |
| A4-B4 | .024\* | .984 | .021\* | .459 | .071† | .224 | .010\* | .242 | .999 | 1.000 | .997 | .996 | 1.000 | .909 | .010\* |
| A4-B5 | .007\*\* | .850 | .006\*\* | .585 | .034\* | .125 | .008\*\* | .610 | 1.000 | 1.000 | .996 | 1.000 | .299 | .508 | .002\*\* |
| A4-B6 | .004\*\* | .958 | .006\*\* | .882 | .021\* | .534 | .024\* | .984 | 1.000 | .833 | 1.000 | .971 | 1.000 | .999 | .003\*\* |
| A5-B1 | .013\* | .534 | .034\* | 1.000 | .038\* | .882 | .064† | .999 | 1.000 | 1.000 | 1.000 | .794 | 1.000 | 1.000 | .004\*\* |
| A5-B2 | .003\*\* | .730 | .027\* | 1.000 | .030\* | .932 | .078† | .909 | 1.000 | 1.000 | 1.000 | .341 | .999 | .965 | .008\*\* |
| A5-B3 | .003\*\* | .867 | .086† | 1.000 | .042\* | .994 | .038\* | .730 | 1.000 | 1.000 | 1.000 | .559 | 1.000 | .998 | .005\*\* |
| A5-B4 | .007\*\* | .882 | .177 | 1.000 | .086† | .921 | .078† | .559 | 1.000 | .999 | 1.000 | .508 | 1.000 | 1.000 | .008\*\* |
| A5-B5 | .002\*\* | .411 | .047\* | 1.000 | .047\* | .814 | .071† | .774 | 1.000 | 1.000 | 1.000 | .997 | .950 | 1.000 | .004\*\* |
| A5-B6 | .002\*\* | .707 | .071† | 1.000 | .024\* | .992 | .137 | .998 | .984 | .585 | .971 | .459 | 1.000 | .999 | .004\*\* |
| A6-B1 | .030\* | .683 | .004\*\* | 1.000 | .105 | .942 | .013\* | 1.000 | .996 | 1.000 | .683 | .484 | .850 | .994 | .003\*\* |
| A6-B2 | .008\*\* | .794 | .004\*\* | 1.000 | .086† | .984 | .024\* | .999 | .942 | 1.000 | .965 | .105 | .794 | .683 | .008\*\* |
| A6-B3 | .005\*\* | .794 | .010\* | 1.000 | .115 | 1.000 | .007\*\* | .987 | 1.000 | 1.000 | .850 | .242 | .896 | .980 | .003\*\* |
| A6-B4 | .015\* | .833 | .017\* | 1.000 | .260 | .997 | .017\* | .794 | .999 | 1.000 | .909 | .115 | .850 | 1.000 | .005\*\* |
| A6-B5 | <.001\*\* | .559 | .006\*\* | 1.000 | .115 | .980 | .010\* | .984 | 1.000 | .980 | .896 | .774 | .999 | 1.000 | .003\*\* |
| A6-B6 | .005\*\* | .774 | .013\* | 1.000 | .086† | 1.000 | .042\* | 1.000 | 1.000 | .992 | 1.000 | .115 | .990 | .994 | .003\*\* |
A: Children; B: Adults.
1: Neutral-Cong., 2: Neutral-Incong., 3: Angry-Cong., 4: Angry-Incong., 5: Happy-Cong., and 6: Happy-Incong.
**, p < .01; *, p < .05; †, p < .1.
